# Evaluation of a new clinical test protocol for pointing acuity - An initial validity study

**DOI:** 10.1371/journal.pone.0356388

**Published:** 2026-08-19

**Authors:** Benjamin Hägglund, Johan Jirlén, Christina Brogårdh, Elisabeth Ekstrand, Ulrik Röijezon

**Affiliations:** 1 Department of Clinical Sciences, Lund, Orthopedics, Lund University, Lund, Sweden; 2 Department of Neurosurgery and Pain Rehabilitation, Skåne University Hospital, Lund, Sweden; 3 Department of Health, Education, and Technology, Luleå University of Technology, Luleå, Sweden; 4 Department of Health Sciences, Lund University, Lund, Sweden; 5 Department of Neurology, Rehabilitation Medicine, Memory Disorders and Geriatrics, Skåne University Hospital, Malmö, Sweden; 6 Department of Hand Surgery, Skåne University Hospital, Malmö, Sweden; Niigata University of Health and Welfare: Niigata Iryo Fukushi Daigaku, JAPAN

## Abstract

Sensorimotor control of the arm and hand can be disturbed in various health conditions causing deviant and unfavourable movement patterns. This study aimed to evaluate a new clinical test protocol for pointing acuity using the Leap Motion Controller (LMC) and assess the concurrent validity against a standard laboratory motion capture system (Qualisys Motion Capture, QMC). Twenty participants (mean age 44.4 years) performed the pointing acuity tests with eyes open and closed, and with left and right hand. Outcome measure was the mean absolute error (AE) in millimeters (mm) of 10 trials. Pearson’s r or Spearman’s rho were used to analyse the correlations between LMC and QMC, and Bland-Altman were used to analyse the agreement. There were moderate to very strong correlations (r: 0.67 to 0.95) between LMC and QMC in tests with eyes closed. In contrast, tests with eyes open showed weak correlations; rho 0.27 for both left and right hand. Bland-Altman showed median bias of ≤ 1.4 mm (LoA 0.1 to 17.5 mm) between LMC and QMC with eyes open, and ≤ 5.1 mm (LoA −31.6 to 21.5 mm) with eyes closed. Assessing pointing acuity using LMC with a standardized test protocol has an acceptable concurrent validity when compared to a 3D-based multi camera system (QMC), supporting its potential use in clinical settings. Future research should evaluate its psychometric properties regarding discriminative validity, reliability and responsiveness in various populations.

## 1. Introduction

Good hand function allows powerful gripping for lifting and carrying heavy loads, quick dynamic actions like throwing or catching, and precision tasks such as buttoning or typing on a smartphone [1–3]. Accurate performance in these activities depends on optimal sensorimotor function, requiring well-coordinated motor commands and sensory information about hand position, movement, and force [4,5]. Manual tasks are largely guided by visual input, but with no visual input available, the sensorimotor control relies on proprioception [6,7]. Proprioception can be defined as conscious and unconscious processes and perceptions of the body’s positions and movements, as well as of weight, force and effort [8]. Various diseases, e.g., rheumatological, neurological, and musculoskeletal disorders with or without traumatic origin, can affect the hand and wrist proprioception and sensori-motor control causing deviant and unfavourable movement patterns associated with pain and disability [9–16].

Rehabilitation interventions have been developed to reduce pain and regain hand function where improving sensorimotor control and proprioception are key features [17–24]. The ability to quantify movement disturbances is therefore important to guide rehabilitation and evaluate intervention effects. Objective and accurate measures usually involve technically advanced equipment in movement science laboratories such as optoelectronic systems, including multiple cameras to track motions in three dimensions (3D) [25] with high measurement accuracy [26,27]. These multi camera systems are however expensive, technically complicated, and time-consuming to use, and consequently rarely applied in clinical settings. A cheaper and more accessible technology is the Leap Motion Controller (LMC) from Ultraleap TM, California, United States. LMC is a small marker-less sensor with optics, that can track hand and finger movements in 3 dimensions (3D), enabling objective measurements also outside the movement science laboratory [26]. This is particularly relevant for clinical assessment of sensorimotor impairments that might not be evident through conventional clinical tests. The LMC has been shown to provide valid measurements of finger, hand, and wrist movements [28–33], supporting its’ potential for clinical use.

Previous evaluations of the LMC’s validity and reliability in human participants have primarily focused on the accuracy of the LMC technology in various experimental setups [34,35], while using the sensor for assessment of hand sensori-motor control in clinical practice is scarce. One way to assess sensorimotor control and proprioception of the arm and hand is through goal directed movements, such as pointing acuity, with and without the aid of vision [36]. Pointing is a functional, measurable task that is simple, swift, and reproducible that can be used in the clinical setting, similar to the finger-nose test [37]. In a recent pilot study, we evaluated the pointing acuity test presented in this study, on a clinical group of participants with wrist disorder, with promising results regarding discriminant validity and test-retest reliability for the test performed with eyes closed [38]. However, before implementation in clinical settings, it is important to evaluate how exact the test protocol can measure what it is intended to measure, i.e., its’ concurrent validity.

The objective of this study was to evaluate the concurrent validity of a novel pointing acuity test, performed with eyes open and closed, using the LMC compared to a standard laboratory multi camera motion capture system.

## 2. Materials and methods

### 2.1. Study design

This study has a cross-sectional design, in which data from the LMC and the Qualisys Motion Capture (QMC, Qualisys Inc, Sweden) system were collected. All measurements were performed at the Human Health and Performance Lab – Movement Science at Luleå University of Technology (LTU), by a physiotherapist with experience in hand rehabilitation (BH). When reporting the data the STROBE guidelines were followed [39].

### 2.2. Participants

Twenty participants (10 men and 10 women) were recruited through LTU message boards, social media, and email. Inclusion criteria were individuals over 18 years, with or without musculoskeletal pain or hand impairments, in order to evaluate the test protocol in a heterogeneous group with varied movement behaviour. Exclusion criteria were considerably reduced range of motion or neuromuscular dysfunction of the hand or arm, or cognitive dysfunction that would hinder the participant’s ability to complete the task, and visual impairment not correctable with glasses or contact lenses. The study was conducted according to the Declaration of Helsinki and approved by the Swedish Ethics Review Authority (ref. No. 022-03946-01). Participant enrollment began November 27, 2022. Signed informed consent to participate was collected from all participants.

### 2.3. Background information

All participants responded to background questions before performing the pointing acuity test, such as age, weight, height, hand dexterity, pain levels, hand function, and impairments related to the wrists and hands. Pain intensity was measured using the Numerical Rating Scale (NRS), ranging from 0 (no pain) to 10 (worst imaginable pain) and impaired hand function through a binary (yes/no) response. Hand function was also assessed by asking whether participants regularly (daily or almost daily) performed activities requiring fine hand precision and movement control, such as playing a musical instrument or a racquet sport.

### 2.4. Equipment for the pointing acuity test

**Fig 1:**
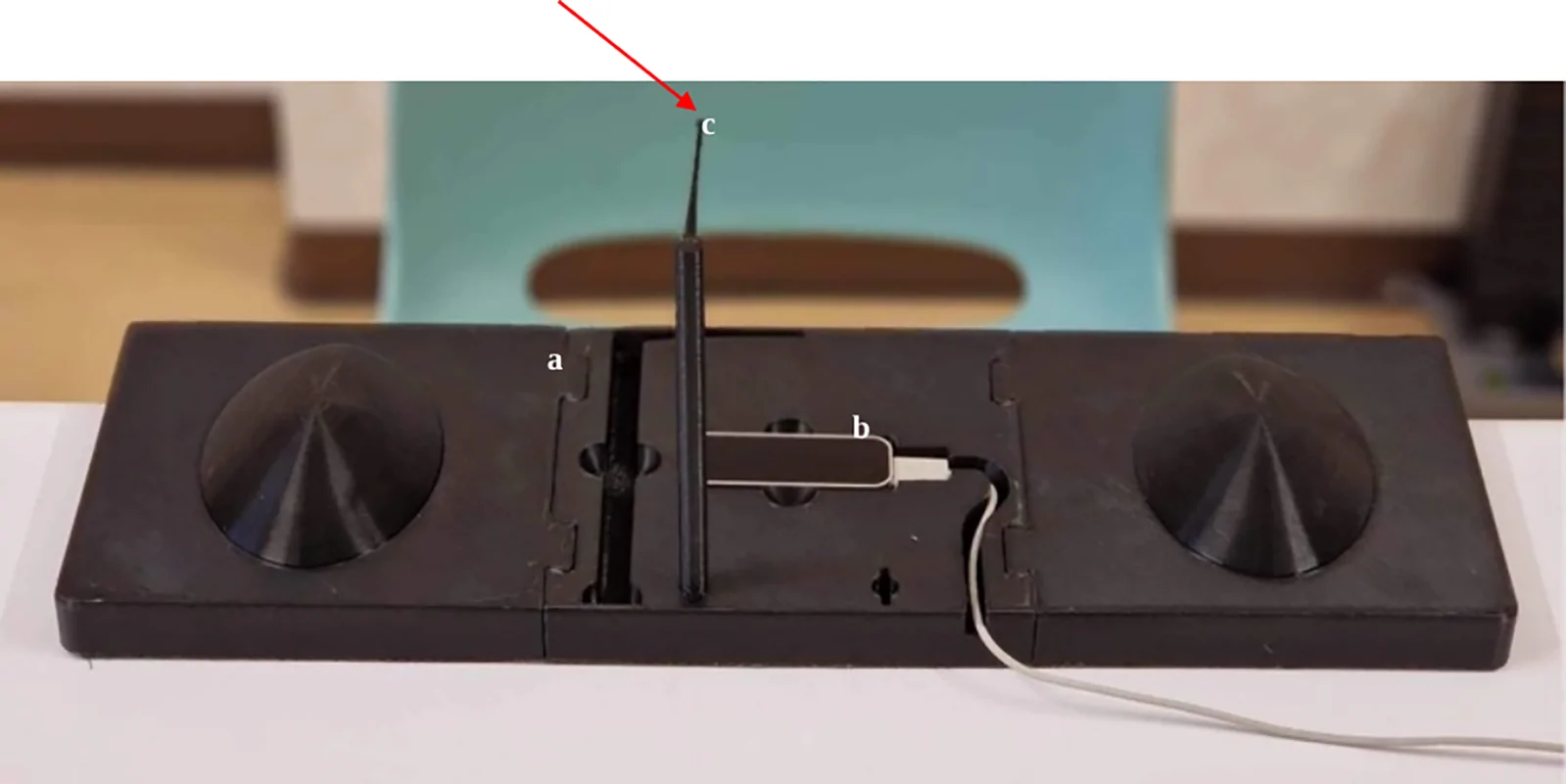
Experimental setup for recording targeted pointing movements with right and left hand with Leap Motion Controller. (A) prefabricated platform. (B) Leap Motion Controller. (C) Target position left hand (red arrow).

In this study the first generation of LMC sensor V2 (desktop) together with the Leap Motion V2 Software Development Kit (SDK) were used. The LMC was supplied at www.ultraleap.com. and used together with a custom-made software developed for tracking the index finger to obtain pointing acuity data. The test was standardized by a custom-made platform fabricated by a 3D printer (45-centimeter wide and 14-centimeter-deep) with a cavity for the LMC, a target (plastic stick) placed 20 cm above the sensor and a marked starting position 30 centimeters from the target (Fig 1).

**Fig 2:**
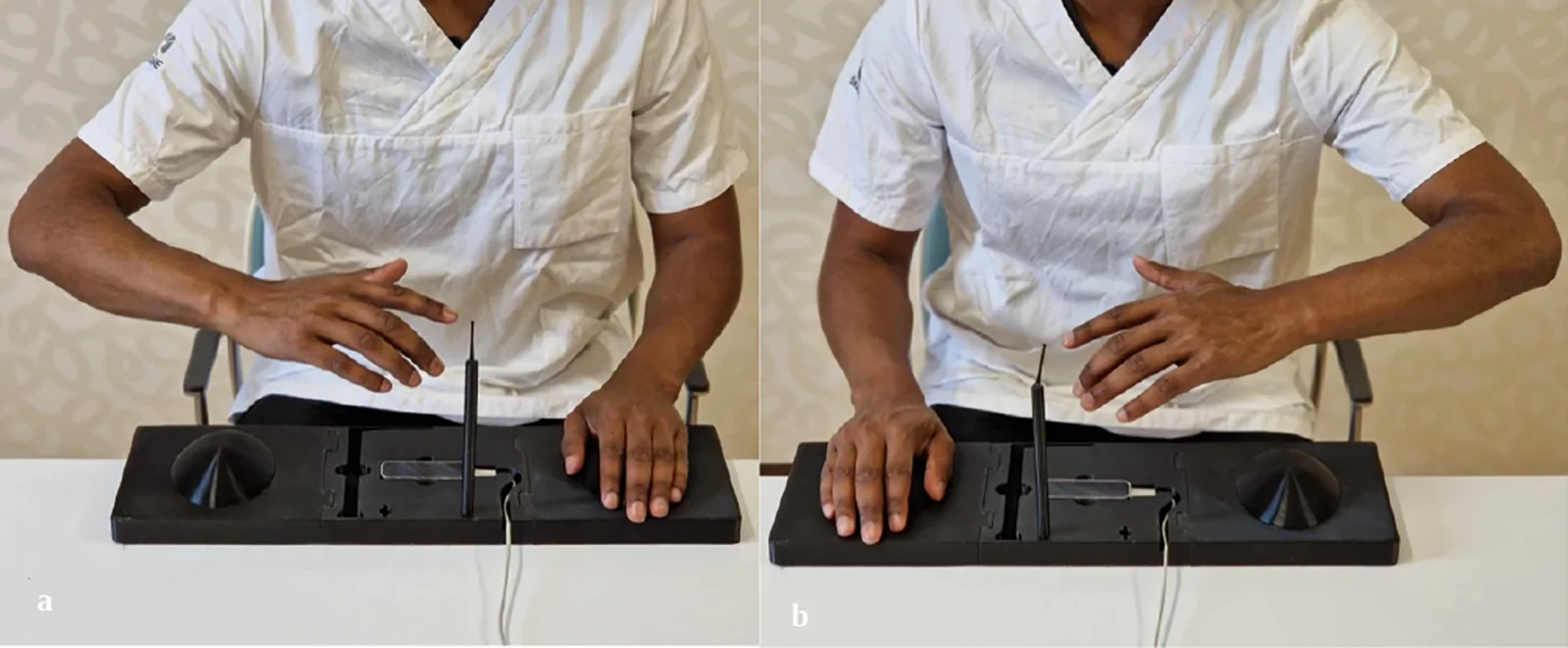
Illustration of the target position for a) right and b) left hand, respectively.

QMC is a standard laboratory system that accurately captures three-dimensional human movement [26,27], included an 8-camera setup (Qualisys TM AB, Sweden) with Qualisys Track Manager (QTM) software. A retro-reflective tape was placed around the tip of the participant’s index fingers as a reference point for the QMC system to track the hand movements. The position of the index finger at the target position, but without the reflective tape, is shown in Figs 2a and 2b.

### 2.5. Description of the test procedure

Data collection was conducted in a human movement science laboratory designed for optical 3D motion capture with white and grey colors and matte finishes on the walls and floor to avoid reflections. The measurement volume was free from visual obstacles and reflective surfaces that could interfere with accurate marker detection. Lighting conditions were carefully controlled, with external light sources and window coverings throughout all data collection sessions to ensure consistent camera performance. Prior to each session, the motion capture system was calibrated according to the manufacturer’s guidelines to ensure high measurement accuracy. The experimental environment remained unchanged throughout the study to minimize variability in recording conditions.

The standardized test began with participants seated at a table, feet on the floor and back against the chair, with the custom-made platform placed in front of them. Hands were positioned at the marked start positions: right on the right, left on the left of the target (Fig 2). All participants were tested within the same measurement area under standardized conditions, including identical equipment setup, marker placement, and test procedures

The pointing acuity test was performed both with the right and left hand, and with eyes opened and closed, starting with eyes opened. The task was to reach the target position with the index finger as accurately as possible, with a pre-determined pace of two seconds between the start and stop of each hand movement. The pace was set by a metronome within the custom-made software, giving a beep sound with two second intervals. To find the right pace, the participants were instructed to alternatively reach forward placing the index finger on the target and then return to the marked starting position just before the beep.

Each of the tests started with a familiarization and warm-up procedure consisting of guided trials with both visual and verbal instructions and feedback until the participant felt familiar with the task and the pace of the movement. Thereafter, a calibration of the target position of the index finger was performed by holding the index finger on the target and memorizing this exact position for 3 seconds, meanwhile, an initial measure was taken. This position was repeated as accurately as possible during the 10 trials. For each trial, a measure of the position of the index finger was taken automatically with the LMC-software when the beep sounded and the hand was at the target, but not if the hand was outside the view of the sensor, as was the case when the hand was at the starting resting position. The software automatically calculated the position of each trial in relation to the first measurement of the position of the index finger. The same method was used for QTM data, which was imported into MATLAB version R2023a, with Phased Array System Toolbox for calculation of the measurements variables. No filtering procedures were applied to the data from LMC or QTM.

To synchronize the time point of the measures between the QTM and the LMC a manual trigger button was used to set events in QTM when the beep sounded from the LMC software. Due to the delay related to reaction time pushing the trigger button after the beep sounded, we chose the index finger position 350 milliseconds (ms) before the event marked in the QTM software. The 350 ms was chosen after inspection of the raw to assure the most suitable time point.

**Fig 3:**
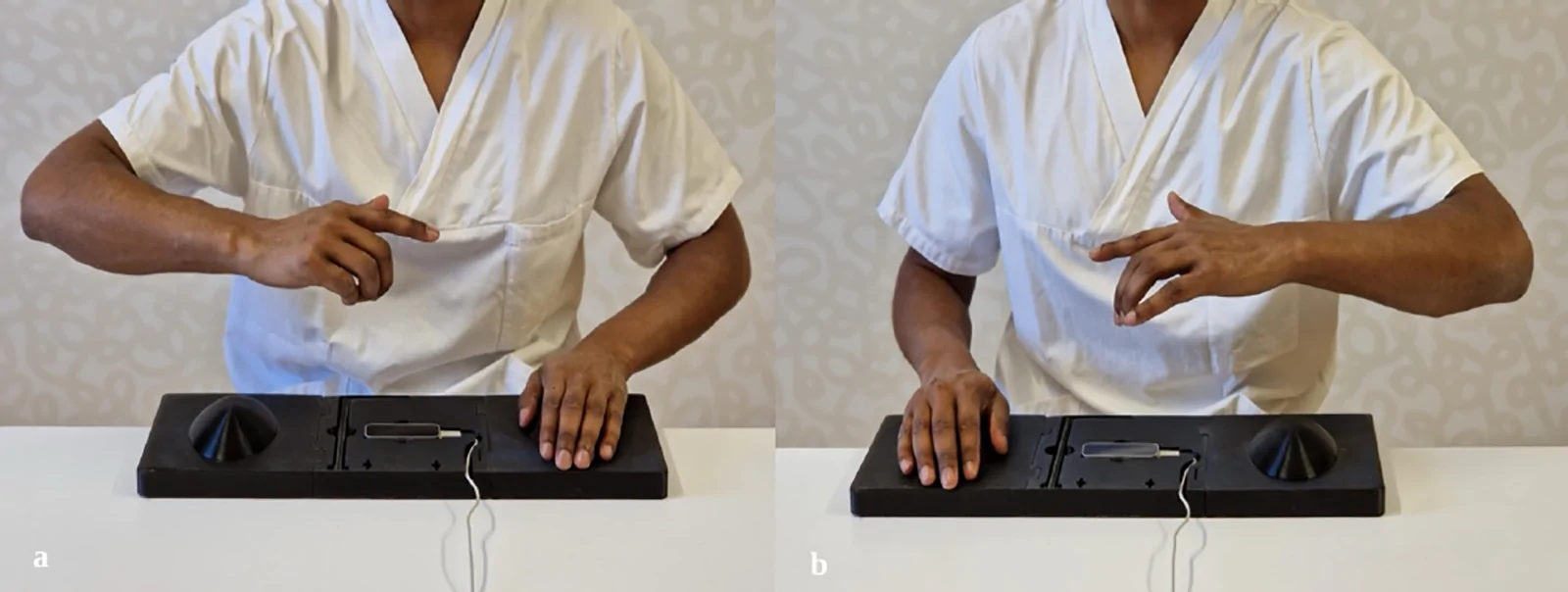
Illustration of the target position without fixed target for a) right and b) left hand, respectively.

During the test with eyes open, the target remained in the same position during the whole test, while the target was removed after the calibration measure when performing the test with eyes closed to avoid any tactile input (Fig 3). In total, the tests took 15–20 minutes to carry out with both hands, and with eyes open and eyes closed.

### 2.6. Outcome measures

The outcome measures were based on the distances between the participant’s individually calibrated target position and the repeated measurement points of the 10 trials in 3D. This was calculated as the mean absolute error (AE) in millimetres (mm) of the 10 trials. Absolute error is commonly used when calculating positions [40]. The AE is defined as the mean value of the distances between the target position, and all considered measured positions, that is:


$$\begin{array}{c} AE=\frac{\text{1}}{n}\sum \sqrt{{\left(x_{i}-x_{\text{0}}\right)}^{\text{2}}+{\left(y_{i}-y_{\text{0}}\right)}^{\text{2}}+{\left(z_{i}-z_{\text{0}}\right)}^{\text{2}}} \end{array}$$

where ($x_{\text{0}},\;y_{\text{0}},\;z_{\text{0}}$) is the target position, and ($x_{i},\;y_{i},\;z_{i}$) is the measurement position for measurement number i, and n is the number of considered measurements (10 in this case). This gives an absolute difference from the reference point regardless of direction and gives an indication of the accuracy without consideration of error direction (directional bias) values (the measurements) and the true value (the target position). The data collection and calculation were made automatically by the custom-made software for the LMC. Data from QMC system was exported from QTM to MatLab for calculation of the outcome variables.

### 2.7. Statistical analysis

Data was analysed with Statistical Package of Social Sciences (SPSS), IBM, version 29. Shapiro–Wilk test was used for the assessment of the normality of data, combined with a visual analysis of histograms, skewness, and kurtosis. Differences between the LMC and QMC-data were analysed with paired samples t-tests, or Wilcoxon Signed rank test if not normally distributed to evaluate any systematic bias between the two measurement systems. A p-value < 0.05 was considered significant.

Concurrent validity between LMC and QMC was evaluated by Pearson or Spearman correlation coefficients (rho) and by Bland & Altman (B&A) plot analysis of absolute measures. Pearson’s r and Spearman’s rho between 0.0–0.39 indicate a weak correlation, 0.4–0.69 moderate, 0.7–0.89 strong, and 0.9–1.00 very strong correlation [41,42]. The Bland-Altman plot analysis gives information about agreement between measures and gives an idea if the LMC and QMC assign similar position values within the same participant. The analysis shows the differences between the two systems (LMC minus QMC) plotted against the mean of both measures. The mean difference is presented together with 95% Limits of Agreement (LoA) which describes the mean difference ±1.96 x SD [43]. When the differences between LMC and QMC were not normally distributed a modified non-parametric Bland Altman analysis was used, including median bias and 10th and 90th percentile as LoA [44].

## 3. Results

### 3.1. Participants

In Table 1, background information of the 20 participants is presented. Five reported previous hand injuries from falls, infections, or lacerations, with a mean pain score of 2.8 (NRS 0–10); two had right-hand pain, two left-hand, and one pain in both hands. Four reported reduced strength and range of motion in the affected hand. No adverse effects, such as discomfort, pain, or fatigue were reported during or after the tests.

**Table 1:** Background information of the 20 participants.

Gender; men/women (n)	10/10
Age; mean (SD) years	44.4 (15.0)
Weight; mean (SD) kg	79.8 (12.6)
Height; mean (SD) cm	175.1 (9.5)
Dominant hand; right/left (n)	18/2
Demands on the hand; normal/high (n)	16/4
Pain in one or both hands (n)	5
Self-reported reduced hand function (n)	4

Values are presented as mean ± standard deviation (SD). n = number of participants.

### 3.2. Concurrent validity between LMC and QMC data

In Table 2 and 3, data are presented as the average AE measurements in the combined horizontal, vertical, and depth planes of the 10 trials in mm for each test. There was a statistically significant difference between LMC and QMC for tests with eyes open (right and left hand). The median AE for LMC was 4.1 mm for the right hand and 4.5 mm for the left hand, and slightly smaller for QMC, with AE of 2.7 mm and 3.2 mm for right and left hand, respectively (Table 2).

**Table 2:** Comparison of measurements between LMC and QMC with eyes open.

Eyes open	QMC Median (IQR) mm	LMC Median (IQR) mm	Median difference mm	Percentile mm		z-value	P -value
				10^th^	90^th^		
Right hand	2.7 (1.8 - 4.2)	4.1 (3.2 - 5.2)	1.4	−.4	3.5	−3.1	.002
Left hand	3.2 (2.0 - 3.6)	4.5 (3.7 - 6.2)	1.3	.1	17.5	−3.5	<.001

Results are presented as median, interquartile range (IQR) and median difference between Qualisys Motion Capture (QMC) and Leap Motion Controller (LMC) absolute error (AE) in millimeters (mm) test statistic from Wilcoxon signed-rank test (z-value) and significance (P -value).

**Table 3:** Comparison of measurements between LMC and QMC with eyes closed.

Eyes closed	QMC Mean (SD) mm	LMC Mean (SD) mm	Mean difference (mm)	Std. Error of Mean	95% Confidence Interval of the difference		t-value	P-value
					Lower	Upper		
**Right hand**	39.5 (17.0)	35.4 (17.5)	−4.1 (10.9)	2.3	−9.2	1.0	−1.7	.108
**Left hand**	36.7 (15.5)	31.6 (17.4)	−5.1 (13.6)	3.0	−11.4	1.3	−1.7	.111

Results are presented as mean, standard deviation (SD) and mean difference between Qualisys Motion Capture (QMC) and Leap motion controller (LMC) absolute error (AE) in millimeters (mm), test statistic from t-test(t-value), and probability (P -value). Negative values represent lower values for LMC, positive values represent higher values for LMC.

Data from the test performed with eyes closed were normally distributed. The AE’s were considerably and significantly larger (p < 0.001) with eyes closed compared to eyes open (S1 Table). For the LMC, the mean AE was 35.4 mm for the right hand and 31.6 mm for the left hand, this was slightly but not significantly larger for the QMC, with AE mean of 39.5 mm and 36.7 mm. (Table 3).

**Fig 4:**
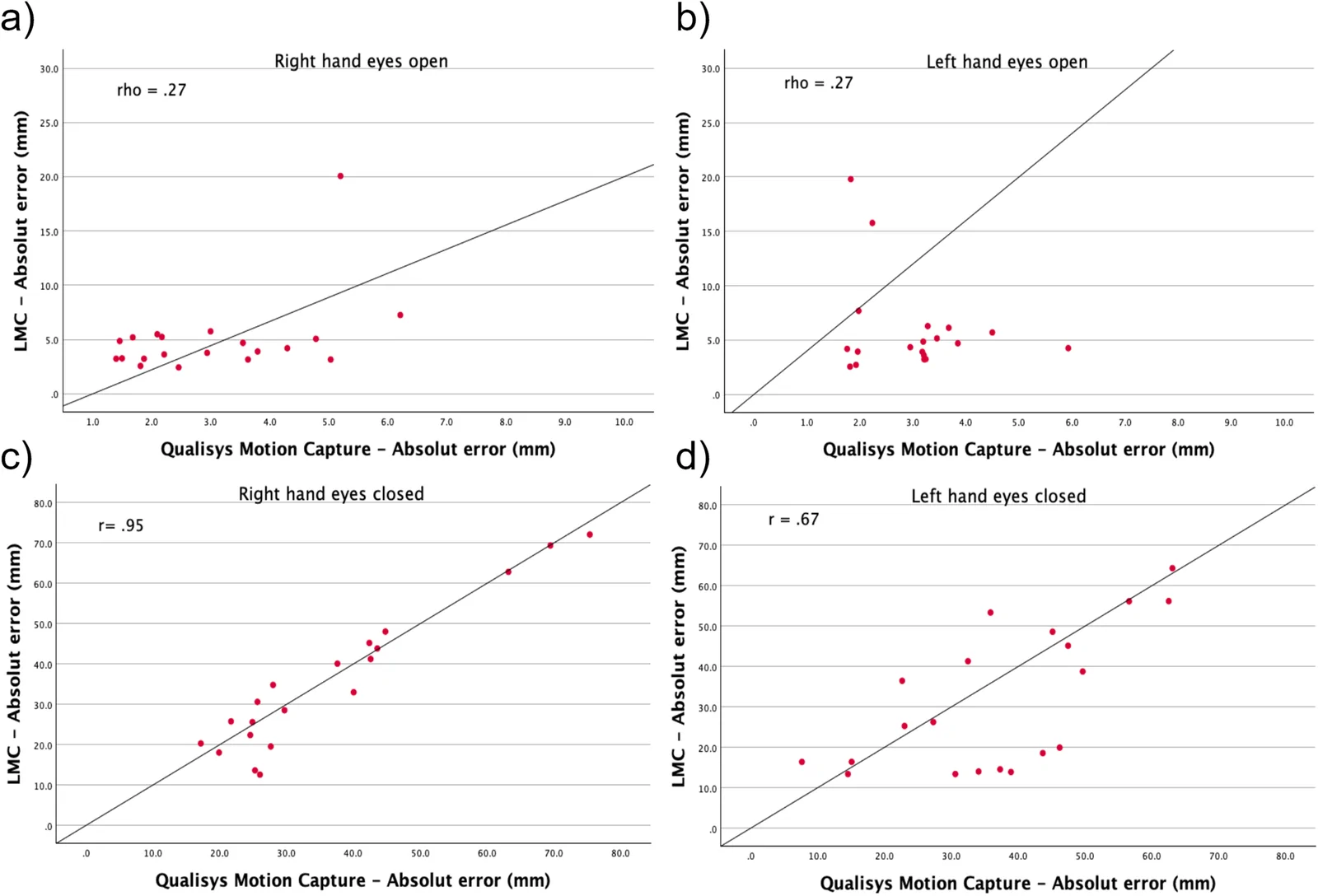
Scatterplot of the absolute error in millimeter (mm) for Leap motion controller (LMC) on the y-axis and Qualisys Motion Capture (QMC) on the x-axis. Each point represents a measurements variable. The linear regression line indicates the overall relationship and degree of correlation between the methods.

The correlations (rho) between the LMC and QMC measurements were 0.27 for both hands with eyes open. The correlations (r) between the LMC and QMC measurements were 0.67 for left hand and 0.95 for right hand with eyes closed (Table 4) and (Fig 4).

**Table 4:** Measurement correlations of right and left hand with eyes open and closed.

Eyes open	Spearman’s rho	95% CI
Right hand	.27	−.21 to.65
Left hand	.27	−.21 to.65
**Eyes closed**	**Pearson’s r**	
Right hand	.95***	.88 to.98
Left hand	.67***	.32 to.86

Correlation between Leap motion controller (LMC) average and Qualisys (QMC) average AE values based on Spearman correlation coefficients for eyes open and Pearson correlation coefficients for eyes closed. ***= p-value <0.001.

**Fig 5:**
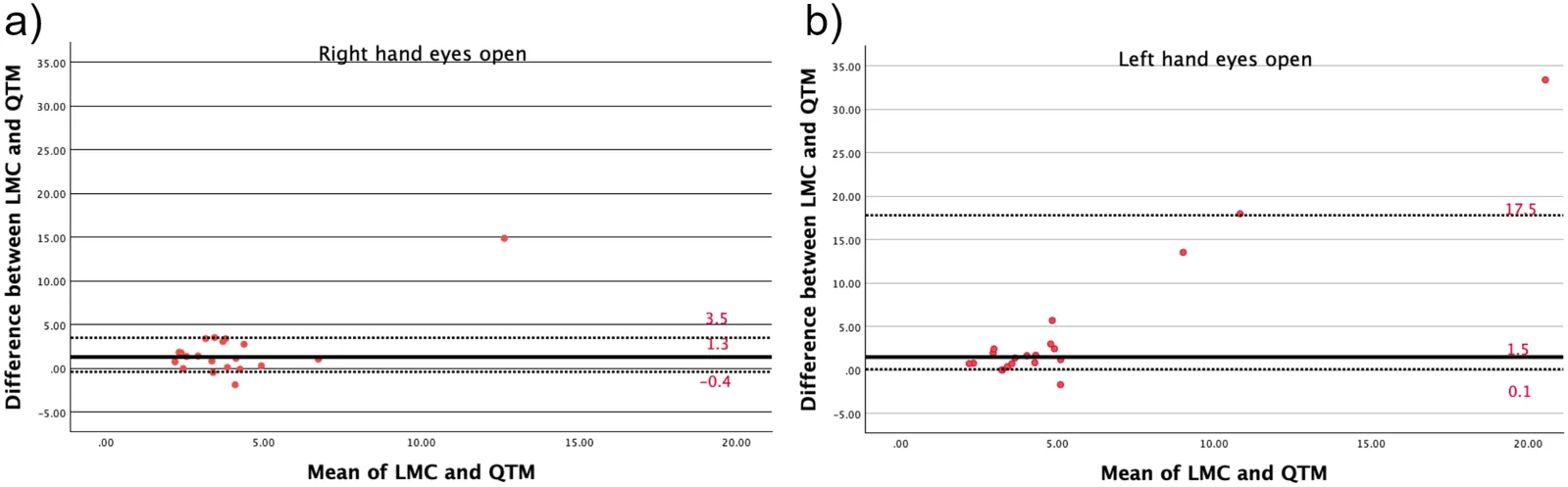
Bland Altman plots comparing Leap motion controller (LMC) and Qualisys Motion Capture (QMC) for tests with eyes open. The y-axis represents the differences between the two methods, and the x-axis the mean values of the two methods. The marked line represents the median difference (bias) between QMC and LMC). Dotted lines represent the Limits of agreement (LoA). The upper dotted line is the 90th percentiles, and the lower dotted line is the10th percentiles.

Bland Altman plots (Fig 5) illustrate the agreement between the LMC and QMC for the tests with eyes open. Median differences were 1.4 mm (LoA −0.4 to 3.5 mm) and 1.3 mm (LoA 0.1 to 17.5 mm) for the right and left hand, respectively (Table 5).

**Table 5:** Agreement between LMC and QMC precented as bias and LoA in millimeters (mm).

Eyes open	Median bias	LoA Percentiles	
		10^th^	90^th^
Right hand (mm)	1.4	−.4	3.5
Left hand (mm)	1.3	.1	17.5
**Eyes closed**	**Mean bias**	**95% LoA**	
		**Lower**	**Upper**
Right hand (mm)	−4.1	−25.4	17.2
Left hand (mm)	−5.1	−31.6	21.5

Mean and median bias with 95% limits of agreement (LoA) and 10th and 90th LoA for Bland Altman. Values are based on Leap motion controller (LMC) and Qualisys Motion Capture (QMC) average absolute error (AE) values.

**Fig 6:**
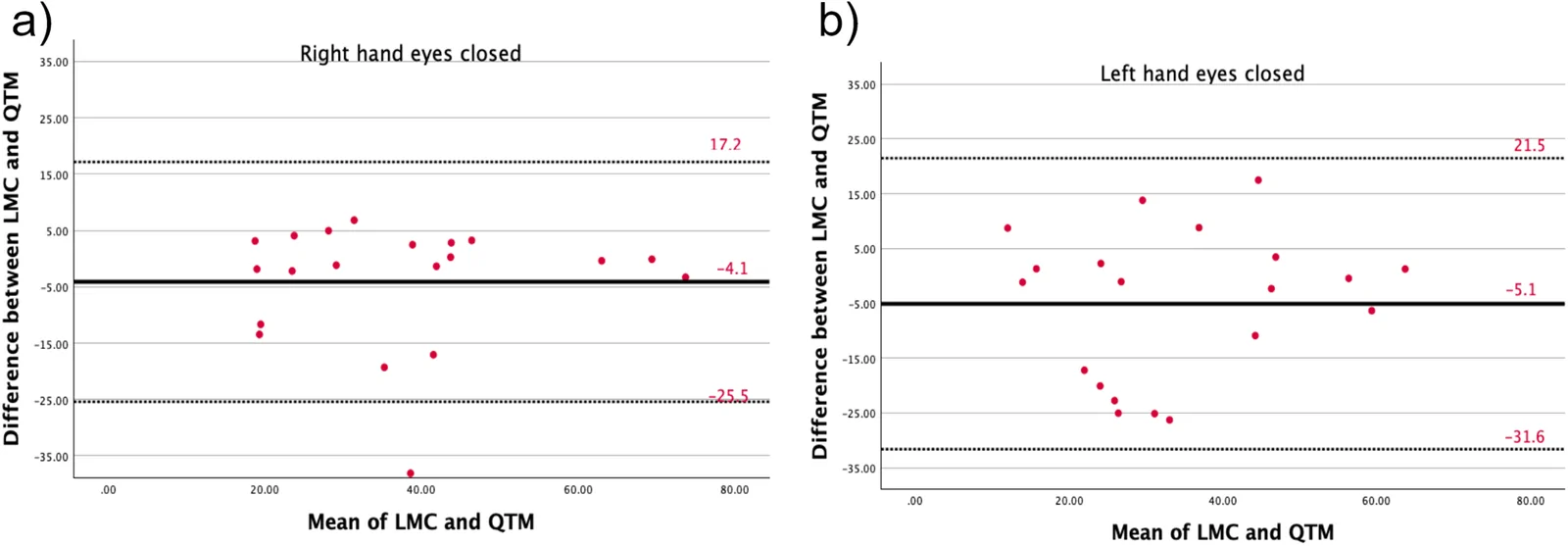
Bland Altman plots comparing Leap motion controller (LMC) and Qualisys Motion Capture (QMC) for tests with eyes closed. The y-axis represents the differences between the two methods, and the x-axis the mean values of the two methods. The marked line represents the mean difference(bias) between QMC and LMC. Dotted lines represent the upper and lower 95% Limits of agreement (LoA) with 1.96 standard deviations from the mean.

Bland Altman plots (Fig 6) illustrate the agreement between mean AE measurements between LMC and QMC for the tests with eyes closed. The mean difference was −4.1 mm (LoA −25.5 to 17.2 mm) and −5,1 mm (−31.6 to 21.5 mm), for the right and left hand, respectively (Table 5).

## 4. Discussion

The objective of this study was to evaluate a new clinical test protocol for pointing acuity using the LMC and assess its concurrent validity with a standard laboratory motion capture system. Results showed that the agreement (according to the Bland-Altman plots) was good for tests with eyes open, but that there were slightly larger bias for tests with eyes closed. Conversely, we found moderate to very strong correlations between LMC and QMC for tests with closed eyes, and weak correlations with eyes open.

In the eyes open tests we found small but statistically significant median biases between LMC and QMC of ≤1.4 mm and LoA ranging from 0.4 to 17.5 mm (Table 5). The LMC tended to consistently produce slightly higher values compared to QMC. However, both the median bias and LoA with eyes open were smaller in our study, i.e., showed better agreement, compared to the studies by Tung et al. [34] and Niechwiej-Szwedo et al [35]. They evaluated the accuracy of LMC in an eye-hand coordination test and compared it with a similar camera system as we used. Tung et al. [34] found a mean measurements deviation between LMC and the multi-camera system of 17.3 mm (SD 9.6 mm). Niechwiej-Szwedo et al. [35] showed a mean bias of 4.5 mm (SD 19.9), and LoA for each participant ranging between ±16.8 mm to ±33.3 mm.

In the eyes-closed tests, we found larger differences than in the eyes-open tests, with mean bias ≤ 5.1 mm (LoA −31.6 to 21.5 mm). There was a tendency that LMC showed smaller AE compared to QMC (Table 2) but this was not statistically significant. Our findings for eyes closed tests are comparable with the studies by Tung et al. [34] and by Niechwiej-Szwedo et al. [35]. Both studies used the LMC compared with an advanced optical motion-capture system. Data acquisition was initiated simultaneously in both systems, and the task requirements were similar, consisting of a pointing movement toward a target position. Taken together, our results regarding agreement are favourable (eyes open tests) or consistent (eyes closed tests) with previous research [34,35]. However, the studies of Tung et al. (2015) and Niechwiej-Szwedo et al. (2018) used a more general pointing task that was visually guided, whereas our study employed a standardized test protocol performed both with eyes open and eyes closed. Furthermore, differences in test setup, procedural standardization, analytical methods, and participant characteristics somewhat limit the comparability of their findings with ours.

In the present study agreement was good with eyes open, but limited with eyes closed, i.e., the bias and LoA were larger when AEs were larger. This may be related to the position of the hand and index finger above the sensor during the measurements. With eyes open the tip of the index finger was always close to the LMC’s optimal tracking zone and target, which was positioned 20 cm vertically above the sensor, while during eyes closed the fingertip could deviate several cm in the three movement planes, which is associated with reduced measurement precision. Previous research has also reported various accuracy due to hand position within the measurement area of the LMC [36]. However from a clinical perspective, the limits of agreement (LoA) should be interpreted in relation to the intended application, and the variability does not preclude clinical relevance. The wider LoA observed in the eyes-closed may limit the precision of the measure for individual-level decision-making, but may be more appropriate for assessing broader trends, such as group-level differences or changes over time, rather than for precise individual evaluation. These findings highlight the importance of considering both the context of use and the inherent variability of the measurement when interpreting agreement metrics in clinical settings.

Moreover, we found poor correlations (rho = 0.27) between the LMC and QMC for tests with eyes open (see Table 4). Our results differ with the study by Tung et al. [34] who found strong correlations (r = 0.95–0.99) when comparing LMC to a standard laboratory camera system. A possible reasons for the weak correlations in our study may be the small variability in AE between the participants, which reduces the sensitivity of correlation analyses. In contrast, for eyes closed (Table 4) the correlations were moderate to very strong (r = 0.67 and 0.95) which might be due to the larger variability between participants. As we have not found other studies evaluating the LMC during manual tasks without visual feedback our findings are difficult to compare with previous research.

In summary, the analyses of agreement and correlations provide a comprehensive view of concurrent validity of the LMC in this novel pointing acuity test [45]. Although the correlation for the eyes open test was poor, probably because of the small measurement variability, bias was negligible. The correlation for the eyes closed test was moderate to very strong, but bias was greater, probably due to reduced precision of LMC in the peripheral regions of its tracking zone. Thus, the concurrent validity of LMC in this initial study seems acceptable, but should be interpreted in relation to its intended application.

### 4.1. Methodological considerations

Several methodological aspects could have influenced our results. The infrared light from the QMC system could potentially have affected the LMC measurements as previous studies have reported LMC to be sensitive to lighting conditions [46]. The timing of movement posed challenges for some of the participants, which potentially could have affected the results and may have caused the outliers found in the Bland-Altman plots (Figs 5 and 6). Measurements were recorded with the LMC only when the index fingertip was at the target position, guided by the metronome. The position of the index finger was not measured if the hand was outside the device’s field of view when positioned at the starting position. Difficulty in accurately tracking fingertip trajectories, particularly if hands rotate or are outside the device’s field of view has been reported in previous studies [29,46]. The ongoing technological development of cheaper and user-friendly sensors is expected to continue, yielding improvements in both measurement volume and precision.Therefore, several sources of error identified in this study are likely to be less influential in the future.

Furthermore, the QMC measurement was manually timed with a custom software metronome and then adjusted to match LMC measurement, potentially contributing to synchronization issues between LMC and QMC. This could be a possible explanation for the biases and outliers that were found for tests (Figs 5 and 6). Optimally, signals from the two measurement systems should be digitally synchronized during the test to assure more accurate comparisons between systems.

### 4.2. Strengths and limitations

A strength was the inclusion of participants of both sexes, with a wide age span and both with and without hand pain or disability. This allowed the concurrent validity to be evaluated with a variety of movement strategies. This is important since, in a clinical setting, movement behaviour of the wrist and hand can vary both between and within individuals. Another strength is that both correlations and agreement between measurements were used in the comparison of the measurement systems [43].

A limitation is the small sample size, which may have contributed to the non-normally distributed data in the eyes-open tests and increased uncertainty in the results. The number of participants was based on estimations from a similar previously published study [47].

Another limitation of the present study is that all participants performed the eyes-open trials prior to the eyes-closed. This may have introduced a potential learning effect, that could have influenced performance in the eyes-closed condition. As a result, performance of the eyes-closed test may reflect remembered task-related cues rather than purely proprioceptive processing. The participants were instructed to practice until they felt confident performing the task before each test. This procedure was intended to ensure stable performance prior to formal testing, although it can never fully exclude learning effects.

Future studies should preferably be based on sample size calculations to strengthen the evidence of the findings. Furthermore, it should be noted that pointing acuity is not affected only by eye-hand coordination and finger-hand proprioception in isolation but also involves proprioceptive processing and motor strategies involving more proximal joints including the neck, shoulder and elbow. Finally, learning effects across repeated trials is an additional factor that also should be considered a potential limitation of the study.

### 4.3. Clinical implications

Valid assessments of proprioception are essential for guiding individualized treatment strategies and evaluating intervention effects. Although this initial evaluation of LMC shows some limitations in measurement accuracy compared to standard laboratory equipment, the findings support its potential role as a complementary measure that can capture clinically relevant aspects of proprioceptive function that are not readily assessed by current methods used in clinical practice.

The LMC sensor is a low-cost, portable, and marker-less system, which could facilitate fast and feasible collection of kinematic data outside of the movement science laboratory. The tests conducted with eyes open could be regarded as assessments of eye-hand coordination, while performing the test with eyes closed puts a higher demand on the proprioceptive system during the pointing acuity task. The clinical applicability of the tests should be further investigated on clinical populations with sensorimotor difficulties of the upper extremity, for example patients with neurological diseases, to mimic the finger-to-nose test, and other health conditions, e.g., rheumatological, orthopaedic and pain conditions that can affect hand function.

### 4.4. Conclusion

Assessing pointing acuity using LMC with a standardized test protocol seems to have acceptable concurrent validity when compared to a standard laboratory system. Future research should evaluate its psychometric properties regarding reliability, discriminative validity, and responsiveness in various populations.

## Supporting information

S1 Table Comparison of mean absolute error measurements between eyes closed and eyes open. Results are presented in mean with, standard deviation (SD), minimum and maximum absolute error measurements. Wilcoxon Signed rank test for comparison of mean between eyes open and closed by Qualisys Motion Capture and Leap Motion Controller measurements.
(DOCX)

S1 File Data. (PDF)
