# Supplementary material for: Evaluation of a new clinical test protocol for pointing acuity - An initial validity study
Source: PLoS One. 2026 Aug 19;21(8):e0356388. doi: 10.1371/journal.pone.0356388 (PMC13489416; doi:10.1371/journal.pone.0356388)
Supplement: Supplementary file 1 [file pone.0356388.s002.pdf]

**Hand/axis**	REO_leap_x	REO_leap_y	REO_leap_z	REC_leap_x	REC_leap_y
	-0.149477938	-0.453338594	-0.800493148	-15.99717941	23.58097077
	-1.795127502	-1.261024407	-2.690143559	-39.3295671	13.17349229
	-0.440730229	0.213211096	-3.470576091	-38.56335818	23.35219572
	-0.662515505	-3.782699629	-1.922244909	-37.5613561	26.18223571
	-0.674530646	-2.39771267	-0.913983679	-31.31736569	22.00087731
	-0.121774983	-3.982261581	-0.54891286	-22.23987789	32.39534767
	0.914485483	0.003585873	-3.659438338	-28.33379785	25.73342139
	-0.615374463	-2.029296833	-3.336632054	-24.09509465	28.84997556
	-5.221621911	0.060195922	-3.158114779	-25.19770847	23.44453411
	0.149477938	0.453338594	0.800493148	15.99717941	23.58097077
	1.795127502	1.261024407	2.690143559	39.3295671	13.17349229
	0.440730229	0.213211096	3.470576091	38.56335818	23.35219572
	0.662515505	3.782699629	1.922244909	37.5613561	26.18223571
	0.674530646	2.39771267	0.913983679	31.31736569	22.00087731
	0.121774983	3.982261581	0.54891286	22.23987789	32.39534767
	0.914485483	0.003585873	3.659438338	28.33379785	25.73342139
	0.615374463	2.029296833	3.336632054	24.09509465	28.84997556
	5.221621911	0.060195922	3.158114779	25.19770847	23.44453411
**Mean**	1.177293185	1.575925179	2.277837713	29.18170059	24.30145006

REC_leap_z	LEO_leap_x	LEO_leap_y	LEO_leap_z	LEC_leap_x	LEC_leap_y
-2.685891938	-0.337780077	-4.259017983	3.134166781	13.38839117	20.44254282
-5.043881847	-1.277852487	-5.508911077	-3.605221905	7.928734161	-11.83650152
-0.3233221	-2.677645942	-2.033050511	0.304270894	-5.453150708	13.93829336
-14.64512852	0.167657128	2.68742371	0.437884165	3.403567559	7.859123066
-17.03577897	2.554650529	2.79883572	-0.036932508	5.010731894	-4.17258494
-16.53992996	-0.512415104	-2.852348181	-3.230522323	0.280975045	8.402443424
-21.10796838	-2.552343707	-0.330032539	4.27609165	14.27648846	-1.0770644
-14.39429874	-0.856015356	2.19601447	-4.00242007	-12.02640546	-5.457092374
-18.30900236	-0.140564131	1.533752322	1.870150742	-1.436989634	-4.900047782
2.685891938	0.337780077	4.259017983	3.134166781	13.38839117	20.44254282
5.043881847	1.277852487	5.508911077	3.605221905	7.928734161	11.83650152
0.3233221	2.677645942	2.033050511	0.304270894	5.453150708	13.93829336
14.64512852	0.167657128	2.68742371	0.437884165	3.403567559	7.859123066
17.03577897	2.554650529	2.79883572	0.036932508	5.010731894	4.17258494
16.53992996	0.512415104	2.852348181	3.230522323	0.280975045	8.402443424
21.10796838	2.552343707	0.330032539	4.27609165	14.27648846	1.0770644
14.39429874	0.856015356	2.19601447	4.00242007	12.02640546	5.457092374
18.30900236	0.140564131	1.533752322	1.870150742	1.436989634	4.900047782
12.2316892	1.230769385	2.688820724	2.321962338	7.022826009	8.676188188

LEC_leap_z	REO_qtm_x	REO_qtm_y	REO_qtm_z	REC_qtm_x	REC_qtm_y
8.474957679	-2.352263738	0.816216081	1.51694904	-17.41455551	1.36755761
-5.993347833	-2.087387584	1.97610352	0.805841722	-40.07410082	0.005976071
-4.02342009	-1.224962838	1.179486973	2.510575667	-40.70883353	1.139282916
2.656410543	-0.470502849	1.671637943	-1.797759258	-40.73190068	14.76520405
9.983848788	-0.680979823	0.614971711	-0.021403031	-32.93406622	17.41425981
-8.775208678	0.039968607	0.57940751	-0.631268915	-25.09991559	18.54576874
-6.392107602	0.655775815	2.017161074	2.029002442	-30.69682307	22.43224087
-0.741540817	-0.151239708	2.611445106	0.719909452	-25.16396188	15.98563126
-5.243409416	-0.730215716	0.75179273	-0.195702182	-27.66489914	19.28344856
8.474957679	2.352263738	0.816216081	1.51694904	17.41455551	1.36755761
5.993347833	2.087387584	1.97610352	0.805841722	40.07410082	0.005976071
4.02342009	1.224962838	1.179486973	2.510575667	40.70883353	1.139282916
2.656410543	0.470502849	1.671637943	1.797759258	40.73190068	14.76520405
9.983848788	0.680979823	0.614971711	0.021403031	32.93406622	17.41425981
8.775208678	0.039968607	0.57940751	0.631268915	25.09991559	18.54576874
6.392107602	0.655775815	2.017161074	2.029002442	30.69682307	22.43224087
0.741540817	0.151239708	2.611445106	0.719909452	25.16396188	15.98563126
5.243409416	0.730215716	0.75179273	0.195702182	27.66489914	19.28344856
5.809361272	0.93258852	1.357580294	1.13649019	31.16545071	12.32659665

REC_qtm_z	LEO_qtm_x	LEO_qtm_y	LEO_qtm_z	LEC_qtm_x	LEC_qtm_y	LEC_qtm_z
23.77869215	-1.02527503	-2.856074013	3.505236463	3.601451821	3.69766718	3.793882538
14.2757927	-2.766760265	1.313070349	-1.446463437	-1.15955352	-0.872643603	-0.58573369
23.04319413	-3.66580245	5.374464696	1.340782891	1.63848827	1.936193648	2.233899027
25.62846941	-5.531444959	0.892385626	3.207874156	3.634403877	4.060933598	4.487463319
25.09552192	-5.650069736	0.576731452	4.144881618	4.607319507	5.069757396	5.532195285
32.48339245	-6.688334185	0.68891677	4.637562281	5.089133115	5.540703949	5.992274782
25.19183607	-5.17762016	-0.986265122	4.776107588	5.273180755	5.770253922	6.267327089
24.31617333	-4.732732387	1.616501221	3.40266405	3.851830876	4.300997701	4.750164527
21.40207685	-5.607299933	0.412891675	2.719023948	3.116321662	3.513619376	3.91091709
23.77869215	1.02527503	2.856074013	3.505236463	3.601451821	3.69766718	3.793882538
14.2757927	2.766760265	1.313070349	1.446463437	1.15955352	0.872643603	0.585733685
23.04319413	3.66580245	5.374464696	1.340782891	1.63848827	1.936193648	2.233899027
25.62846941	5.531444959	0.892385626	3.207874156	3.634403877	4.060933598	4.487463319
25.09552192	5.650069736	0.576731452	4.144881618	4.607319507	5.069757396	5.532195285
32.48339245	6.688334185	0.68891677	4.637562281	5.089133115	5.540703949	5.992274782
25.19183607	5.17762016	0.986265122	4.776107588	5.273180755	5.770253922	6.267327089
24.31617333	4.732732387	1.616501221	3.40266405	3.851830876	4.300997701	4.750164527
21.40207685	5.607299933	0.412891675	2.719023948	3.116321662	3.513619376	3.91091709
23.91279433	4.538371012	1.635255658	3.242288493	3.552409267	3.862530041	4.172650816

Hand/axis

	REO_leap_x	REO_leap_y	REO_leap_z	REC_leap_x	REC_leap_y	REC_leap_z
	1.331734669	0.393882663	0.336432557	-11.89933461	13.56497952	-3.34330233
	-3.854902905	3.922409137	0.539060526	0.367287521	-2.691482464	-5.351346882
	1.309305978	1.926025366	1.599829133	2.431870639	-4.574707176	-1.266902039
	0.848293521	-3.806358306	-2.810142316	10.56379691	22.33401488	-1.838155756
	-2.230861752	2.368530221	-0.122545349	3.933720467	-2.683334341	2.659347227
	-0.863552277	-3.288131718	-3.804851253	5.178849024	-5.166808731	-3.653371918
	4.622504283	4.200180095	4.937434049	-22.05673975	-5.869148388	-1.814570801
	-5.413610523	-3.710174573	-1.131133582	19.61968341	5.450103378	-9.44086685
	2.104655556	2.661743245	3.298124824	-1.478570805	6.194427699	6.575312022
	1.331734669	0.393882663	0.336432557	11.89933461	13.56497952	3.34330233
	3.854902905	3.922409137	0.539060526	0.367287521	2.691482464	5.351346882
	1.309305978	1.926025366	1.599829133	2.431870639	4.574707176	1.266902039
	0.848293521	3.806358306	2.810142316	10.56379691	22.33401488	1.838155756
	2.230861752	2.368530221	0.122545349	3.933720467	2.683334341	2.659347227
	0.863552277	3.288131718	3.804851253	5.178849024	5.166808731	3.653371918
	4.622504283	4.200180095	4.937434049	22.05673975	5.869148388	1.814570801
	5.413610523	3.710174573	1.131133582	19.61968341	5.450103378	9.44086685
	2.104655556	2.661743245	3.298124824	1.478570805	6.194427699	6.575312022
**Mean**	2.508824607	2.919715036	2.064394843	8.614428126	7.614334063	3.993686203

LEO_leap_x	LEO_leap_y	LEO_leap_z	LEC_leap_x	LEC_leap_y	LEC_leap_z
0.648717441	-2.142700188	1.081213488	-7.326104822	-3.526695373	-15.07362598
0.955540816	-1.991454947	0.73135422	-1.229494666	9.11617274	-0.580816746
-43.42236394	9.348136817	-66.25521095	-14.72406371	8.153747765	-2.639923408
42.40414518	-4.565338124	65.916923	-6.862109606	-0.163803297	6.632050863
-0.210457298	-2.305328444	1.238658765	8.545066212	-15.32161702	-15.04284794
1.410440384	-0.063545217	0.743526785	-2.793719173	12.78032692	-4.895385787
-3.398443186	-2.308624216	-1.795917657	3.940321088	-9.009269775	-7.312758526
3.347798046	0.546562171	0.176764838	-0.347653645	8.65846261	5.330275768
-2.681029524	-0.480674724	1.224139376	1.851674538	-17.79574607	-4.876275347
0.648717441	2.142700188	1.081213488	7.326104822	3.526695373	15.07362598
0.955540816	1.991454947	0.73135422	1.229494666	9.11617274	0.580816746
43.42236394	9.348136817	66.25521095	14.72406371	8.153747765	2.639923408
42.40414518	4.565338124	65.916923	6.862109606	0.163803297	6.632050863
0.210457298	2.305328444	1.238658765	8.545066212	15.32161702	15.04284794
1.410440384	0.063545217	0.743526785	2.793719173	12.78032692	4.895385787
3.398443186	2.308624216	1.795917657	3.940321088	9.009269775	7.312758526
3.347798046	0.546562171	0.176764838	0.347653645	8.65846261	5.330275768
2.681029524	0.480674724	1.224139376	1.851674538	17.79574607	4.876275347
10.94210398	2.63915165	15.46263434	5.291134162	9.391760174	6.931551152

REO_qtm_x	REO_qtm_y	REO_qtm_z	REC_qtm_x	REC_qtm_y	REC_qtm_z
0.420935648	-1.651905109	-0.975889233	-12.32971937	2.913254761	12.52012848
-0.494509076	-2.941034788	-1.776385649	-9.862588871	7.292580987	10.19433394
-2.622478965	-3.470811622	-1.066449148	-7.667039215	10.97937233	7.463165965
-1.487828795	-2.212670621	-2.182429455	-2.123295291	13.74475368	28.06984892
-1.188670663	-2.40250961	-1.566550152	6.257476325	7.910945369	27.65817528
-0.837974227	0.60505827	-3.416061125	7.587794401	10.53053686	25.00166098
-0.856237479	-2.142054618	-1.340212844	-15.50708972	13.58321495	17.03632685
-3.604180938	-1.626692386	-0.878048594	3.242275929	23.2214088	18.79326308
-2.293708748	-4.577206771	-0.728816578	9.403359992	18.77683518	24.94312076
0.420935648	1.651905109	0.975889233	12.32971937	2.913254761	12.52012848
0.494509076	2.941034788	1.776385649	9.862588871	7.292580987	10.19433394
2.622478965	3.470811622	1.066449148	7.667039215	10.97937233	7.463165965
1.487828795	2.212670621	2.182429455	2.123295291	13.74475368	28.06984892
1.188670663	2.40250961	1.566550152	6.257476325	7.910945369	27.65817528
0.837974227	0.60505827	3.416061125	7.587794401	10.53053686	25.00166098
0.856237479	2.142054618	1.340212844	15.50708972	13.58321495	17.03632685
3.604180938	1.626692386	0.878048594	3.242275929	23.2214088	18.79326308
2.293708748	4.577206771	0.728816578	9.403359992	18.77683518	24.94312076
1.534058282	2.403327088	1.54787142	8.220071013	12.1058781	19.07555825

LEO_qtm_x	LEO_qtm_y	LEO_qtm_z	LEC_qtm_x	LEC_qtm_y	LEC_qtm_z
0.325376159	-0.836511741	0.689151624	-8.248283896	16.762119	-2.468742518
0.704605263	-1.335196515	-0.449222398	-11.90555857	17.77710917	7.831515557
0.214070662	-0.44635515	-0.53201747	-26.78972784	21.03534214	9.971277775
1.424578953	-1.524989082	2.187632098	-26.69942237	12.70566885	15.05662165
1.017301991	-1.098359779	0.689761346	-25.18184894	30.15525771	-2.275257579
0.546022532	-3.458949359	0.897614998	-23.33309714	35.06476926	12.18479076
0.405743638	-0.4054479	0.112803147	-21.17633821	42.42052665	3.810127777
1.774206117	-1.73955711	0.77928909	-24.61043946	38.13536903	8.061513794
-0.270020431	-1.454137751	-0.401979995	-22.11962364	42.45377333	-7.64278786
0.325376159	0.836511741	0.689151624	8.248283896	16.762119	2.468742518
0.704605263	1.335196515	0.449222398	11.90555857	17.77710917	7.831515557
0.214070662	0.44635515	0.53201747	26.78972784	21.03534214	9.971277775
1.424578953	1.524989082	2.187632098	26.69942237	12.70566885	15.05662165
1.017301991	1.098359779	0.689761346	25.18184894	30.15525771	2.275257579
0.546022532	3.458949359	0.897614998	23.33309714	35.06476926	12.18479076
0.405743638	0.4054479	0.112803147	21.17633821	42.42052665	3.810127777
1.774206117	1.73955711	0.77928909	24.61043946	38.13536903	8.061513794
0.270020431	1.454137751	0.401979995	22.11962364	42.45377333	7.64278786
0.742436194	1.366611598	0.748830241	21.11826001	28.50110391	7.700292808

REO_leap_x	REO_leap_y	REO_leap_z	REC_leap_x	REC_leap_y	REC_leap_z
4.295486921	1.07283783	1.790941066	-69.16921134	21.6583786	56.18700519
11.0827566	-2.628982539	-9.066981172	12.8752686	4.895271301	-17.77279728
-7.485767478	-2.702400214	11.92105892	-5.762691091	-10.86944584	2.777761683
15.0713454	-5.315887451	-6.077566289	-0.19855151	-7.660232496	10.88314442
-16.58488967	-4.803810039	-0.99645336	14.66026151	-3.450615075	-4.169843754
31.84563171	-13.09170559	-8.0131368	-6.781093641	7.285835276	-4.993535795
-11.60559803	11.22634905	12.52065195	34.10161949	-10.66160579	-9.407784345
-5.064794429	3.09463517	-4.05899968	-68.21785707	25.02610806	27.58030717
33.26937064	-33.33479317	-15.50786876	4.324599953	-9.707046729	11.09666904
4.295486921	1.07283783	1.790941066	69.16921134	21.6583786	56.18700519
11.0827566	2.628982539	9.066981172	12.8752686	4.895271301	17.77279728
7.485767478	2.702400214	11.92105892	5.762691091	10.86944584	2.777761683
15.0713454	5.315887451	6.077566289	0.19855151	7.660232496	10.88314442
16.58488967	4.803810039	0.99645336	14.66026151	3.450615075	4.169843754
31.84563171	13.09170559	8.0131368	6.781093641	7.285835276	4.993535795
11.60559803	11.22634905	12.52065195	34.10161949	10.66160579	9.407784345
5.064794429	3.09463517	4.05899968	68.21785707	25.02610806	27.58030717
33.26937064	33.33479317	15.50786876	4.324599953	9.707046729	11.09666904
15.14507121	8.585711227	7.772628666	24.01012824	11.24605991	16.09653874

Hand/axis	LEO_leap_x	LEO_leap_y	LEO_leap_z	LEC_leap_x	LEC_leap_y
	3.566778246	3.314025917	-3.013042781	11.53762876	14.93887318
	-4.145680129	0.418434454	3.413856063	11.10199769	13.17726143
	2.28366506	4.429771119	-2.430283263	-20.04302402	-8.687606938
	0.708069906	-4.774177955	-1.254691535	14.66300861	12.32809441
	-7.000414163	7.925232125	4.101824905	-2.866765794	6.622726543
	3.470826452	-6.762244927	-2.390037748	12.06201459	16.36592852
	-1.76373757	3.240127556	0.736134635	-5.426109264	-8.263229389
	-2.383736492	3.600265664	0.629904892	-0.106150902	13.51863863
	0.981794752	-3.791771289	-6.38964576	7.994008101	-26.55606831
	3.566778246	3.314025917	3.013042781	11.53762876	14.93887318
	4.145680129	0.418434454	3.413856063	11.10199769	13.17726143
	2.28366506	4.429771119	2.430283263	20.04302402	8.687606938
	0.708069906	4.774177955	1.254691535	14.66300861	12.32809441
	7.000414163	7.925232125	4.101824905	2.866765794	6.622726543
	3.470826452	6.762244927	2.390037748	12.06201459	16.36592852
	1.76373757	3.240127556	0.736134635	5.426109264	8.263229389
	2.383736492	3.600265664	0.629904892	0.106150902	13.51863863
	0.981794752	3.791771289	6.38964576	7.994008101	26.55606831
**Mean**	2.922744752	4.250672334	2.706602398	9.53341197	13.38426971

LEC_leap_z	REO_qtm_x	REO_qtm_y	REO_qtm_z	REC_qtm_x	REC_qtm_y
9.898419276	0.777659071	-1.229178888	0.884037865	-35.36152843	-23.2107324
4.409078332	-1.406487382	-0.633095166	2.818559793	-37.65978006	-16.20286549
-2.748452031	1.188672576	-1.500664086	-0.25186839	-26.13617489	-15.44001582
7.329744713	3.004902794	-3.369888149	-0.102506479	-42.1326214	-24.95661533
5.902550276	0.83676855	-3.987703792	-1.538566167	-32.87780474	-23.25584859
4.122937972	6.488423916	-2.230547578	-5.351899382	-47.97065202	-38.86524756
-8.74421419	3.295476213	-2.566243471	-3.745575258	-42.94663558	-28.21850664
-3.409168496	1.368292179	-1.212937447	-5.466505363	-49.44047428	-31.66701529
-5.429304182	9.35658534	2.485095737	-5.216916841	-51.42851487	-37.26441574
9.898419276	0.777659071	1.229178888	0.884037865	35.36152843	23.2107324
4.409078332	1.406487382	0.633095166	2.818559793	37.65978006	16.20286549
2.748452031	1.188672576	1.500664086	0.25186839	26.13617489	15.44001582
7.329744713	3.004902794	3.369888149	0.102506479	42.1326214	24.95661533
5.902550276	0.83676855	3.987703792	1.538566167	32.87780474	23.25584859
4.122937972	6.488423916	2.230547578	5.351899382	47.97065202	38.86524756
8.74421419	3.295476213	2.566243471	3.745575258	42.94663558	28.21850664
3.409168496	1.368292179	1.212937447	5.466505363	49.44047428	31.66701529
5.429304182	9.35658534	2.485095737	5.216916841	51.42851487	37.26441574
5.777096608	3.080363113	2.135039368	2.819603949	40.66157625	26.56458476

REC_qtm_z	LEO_qtm_x	LEO_qtm_y	LEO_qtm_z	LEC_qtm_x	LEC_qtm_y
13.56049201	0.036766498	-1.513955241	0.82326636	11.28937711	-7.722194353
12.08858089	2.784692865	-0.206674052	2.393126748	26.67210822	-12.96810616
10.22823098	0.095736979	-0.094274907	2.44203423	10.09939485	-10.20961718
-3.080674697	0.026482748	-1.033103264	0.650332546	19.35430698	-16.14399557
1.456737857	1.13568008	-1.022937889	3.755530878	29.35970856	-24.26380098
17.24503293	0.037969828	-1.287817312	2.268227915	33.75893216	-34.09222041
9.398915034	0.127456281	-0.538399421	2.720295682	22.71308897	-19.18653545
19.49528008	3.365111031	0.548407416	5.444760405	37.61674533	-18.52076501
2.046369992	-3.276546242	2.44421168	2.060551355	28.20819961	-9.491307341
13.56049201	0.036766498	1.513955241	0.82326636	11.28937711	7.722194353
12.08858089	2.784692865	0.206674052	2.393126748	26.67210822	12.96810616
10.22823098	0.095736979	0.094274907	2.44203423	10.09939485	10.20961718
3.080674697	0.026482748	1.033103264	0.650332546	19.35430698	16.14399557
1.456737857	1.13568008	1.022937889	3.755530878	29.35970856	24.26380098
17.24503293	0.037969828	1.287817312	2.268227915	33.75893216	34.09222041
9.398915034	0.127456281	0.538399421	2.720295682	22.71308897	19.18653545
19.49528008	3.365111031	0.548407416	5.444760405	37.61674533	18.52076501
2.046369992	3.276546242	2.44421168	2.060551355	28.20819961	9.491307341
9.844479386	1.209604728	0.965531242	2.506458458	24.34131798	16.95539361

LEC_qtm_z
11.24508129
20.79618379
17.2922102
24.224059
38.19736335
52.07011969
41.83323007
49.64122671
27.77678337
11.24508129
20.79618379
17.2922102
24.224059
38.19736335
52.07011969
41.83323007
49.64122671
27.77678337
31.4529175

**Hand/axis**	REO_leap_x	REO_leap_y	REO_leap_z	REC_leap_x	REC_leap_y
	0.845454931	-1.433845578	-0.254699242	3.757442164	19.77935789
	-0.428615458	2.179206821	1.560979624	0.569573614	20.39457708
	-11.18146892	-2.380157448	-4.753481184	11.00156203	-1.383422941
	9.375770963	3.017028905	4.712139307	-38.94397916	3.315269424
	1.80385687	0.312354969	-1.457648372	12.54588551	9.400169593
	2.658336765	-1.052368148	-0.989864446	5.078460258	25.6471862
	-4.577008589	-0.085556018	-2.257142832	7.812322935	-5.904098529
	4.961331186	5.153396586	2.176475902	0.705763486	2.019355535
	-2.134895894	2.2761537	-2.183659182	-15.49849995	-2.663390877
	0.845454931	1.433845578	0.254699242	3.757442164	19.77935789
	0.428615458	2.179206821	1.560979624	0.569573614	20.39457708
	11.18146892	2.380157448	4.753481184	11.00156203	1.383422941
	9.375770963	3.017028905	4.712139307	38.94397916	3.315269424
	1.80385687	0.312354969	1.457648372	12.54588551	9.400169593
	2.658336765	1.052368148	0.989864446	5.078460258	25.6471862
	4.577008589	0.085556018	2.257142832	7.812322935	5.904098529
	4.961331186	5.153396586	2.176475902	0.705763486	2.019355535
	2.134895894	2.2761537	2.183659182	15.49849995	2.663390877
**Mean**	4.218526619	1.987785353	2.260676677	10.65705434	10.05631423

REC_leap_z	LEO_leap_x	LEO_leap_y	LEO_leap_z	LEC_leap_x	LEC_leap_y
5.642456281	-0.834622292	1.168998609	-0.47795472	15.91584608	25.93509674
8.889388071	5.184238132	-0.194999716	-3.603930595	1.271087214	18.32538605
-4.087728407	-3.60120264	-0.298538173	-3.629912927	7.435984188	-12.85311891
0.527143827	-0.763466625	2.048927329	8.906735065	-8.494542662	6.145881609
2.792532558	1.673077496	-3.505592389	-6.685441749	-0.890853202	-1.494727924
1.007275307	-1.658285989	1.192024313	4.288491044	-13.52511754	-7.305381889
-14.06857719	-0.765826455	-1.325302154	0.298613277	-17.08141568	3.291496271
1.836441626	1.308963107	-0.667472859	-3.611694318	22.99753377	0.214462285
11.97049365	0.034845559	1.396827667	3.335869532	-8.394410059	19.94297026
5.642456281	0.834622292	1.168998609	0.47795472	15.91584608	25.93509674
8.889388071	5.184238132	0.194999716	3.603930595	1.271087214	18.32538605
4.087728407	3.60120264	0.298538173	3.629912927	7.435984188	12.85311891
0.527143827	0.763466625	2.048927329	8.906735065	8.494542662	6.145881609
2.792532558	1.673077496	3.505592389	6.685441749	0.890853202	1.494727924
1.007275307	1.658285989	1.192024313	4.288491044	13.52511754	7.305381889
14.06857719	0.765826455	1.325302154	0.298613277	17.08141568	3.291496271
1.836441626	1.308963107	0.667472859	3.611694318	22.99753377	0.214462285
11.97049365	0.034845559	1.396827667	3.335869532	8.394410059	19.94297026
5.646892991	1.758280922	1.310964801	3.870960359	10.66742115	10.61205799

LEC_leap_z	REO_qtm_x	REO_qtm_y	REO_qtm_z	REC_qtm_x	REC_qtm_y
3.722804892	-0.071715509	-1.36355664	-0.790684505	-2.962915829	-6.87697767
22.34342924	-0.079338965	0.898620225	-1.666071332	-12.66164515	-19.62441378
-4.738117076	-1.403891983	-1.392995157	1.063943243	-18.03296825	-19.06230001
-3.356782037	-2.99776297	-0.80597842	1.202182314	-23.49755657	-18.54035825
4.819142441	-2.06681689	-0.745118663	1.425524788	-26.21688659	-18.61213207
-13.03490788	-0.062122093	-0.029393324	0.338987103	-35.69622339	-25.94759079
-1.219442266	-1.499680595	2.661479429	0.034374628	-23.77289446	-9.717919529
-0.655783109	-0.050344898	0.144316216	1.493579349	-17.09105118	-13.28950913
17.01603015	0.211301816	-0.766901687	2.159340989	-25.24784409	-17.46195091
3.722804892	0.071715509	1.36355664	0.790684505	2.962915829	6.87697767
22.34342924	0.079338965	0.898620225	1.666071332	12.66164515	19.62441378
4.738117076	1.403891983	1.392995157	1.063943243	18.03296825	19.06230001
3.356782037	2.99776297	0.80597842	1.202182314	23.49755657	18.54035825
4.819142441	2.06681689	0.745118663	1.425524788	26.21688659	18.61213207
13.03490788	0.062122093	0.029393324	0.338987103	35.69622339	25.94759079
1.219442266	1.499680595	2.661479429	0.034374628	23.77289446	9.717919529
0.655783109	0.050344898	0.144316216	1.493579349	17.09105118	13.28950913
17.01603015	0.211301816	0.766901687	2.159340989	25.24784409	17.46195091
7.878493232	0.938108413	0.97870664	1.130520917	20.57555395	16.57035024

REC_qtm_z	LEO_qtm_x	LEO_qtm_y	LEO_qtm_z	LEC_qtm_x	LEC_qtm_y
17.92241196	-0.368146429	0.607816503	-1.191754295	19.75081868	-6.556908047
36.77047279	-1.986013579	1.774492898	0.608477233	30.02976284	-31.51008445
39.83445771	1.826875756	3.234679266	-0.623214869	31.10595028	-26.10223803
40.51603954	0.585631616	-0.585100488	2.260210572	20.50490259	-23.87654009
46.76291689	2.06935814	5.425152426	-1.851573856	31.49689183	-29.5668647
74.97002705	1.486272154	1.35251941	-0.329004464	16.45605104	-16.55706832
62.9706971	2.417519512	2.373671655	-0.706945739	-1.225804143	-16.75718577
68.73527114	0.817539778	3.693262038	0.105927807	17.61486384	-23.44879584
67.07889154	-0.586126217	2.777059899	1.170177554	17.05567005	-38.70245758
17.92241196	0.368146429	0.607816503	1.191754295	19.75081868	6.556908047
36.77047279	1.986013579	1.774492898	0.608477233	30.02976284	31.51008445
39.83445771	1.826875756	3.234679266	0.623214869	31.10595028	26.10223803
40.51603954	0.585631616	0.585100488	2.260210572	20.50490259	23.87654009
46.76291689	2.06935814	5.425152426	1.851573856	31.49689183	29.5668647
74.97002705	1.486272154	1.35251941	0.329004464	16.45605104	16.55706832
62.9706971	2.417519512	2.373671655	0.706945739	1.225804143	16.75718577
68.73527114	0.817539778	3.693262038	0.105927807	17.61486384	23.44879584
67.07889154	0.586126217	2.777059899	1.170177554	17.05567005	38.70245758
50.61790952	1.349275909	2.42486162	0.983031821	20.5823017	23.6753492

LEC_qtm_z

24.29168349
34.61274336
27.76802876
34.22973785
28.94908469
27.55132203
31.13363134
28.59364544
53.33973554

24.29168349
34.61274336
27.76802876
34.22973785
28.94908469
27.55132203
31.13363134
28.59364544
53.33973554

32.27440139

Hand/axis	REO_leap_x	REO_leap_y	REO_leap_z	REC_leap_x	REC_leap_y
	-2.63839778	4.325027358	2.063145135	-4.358515356	31.46434026
	4.817829742	4.508499261	0.470189541	-0.932557183	-0.526687611
	-2.385726722	-2.798271214	-1.898811571	-1.511083861	1.600791819
	-0.109953624	-3.125526407	1.48041719	-0.799545035	12.23363508
	-1.375686661	9.118766556	-1.873430676	11.70810602	-4.925025958
	1.570540427	-0.903998817	3.091721337	1.41939704	-9.837089628
	-1.336111948	-4.652206548	-0.655915527	-6.244997622	-2.557678298
	2.764219173	1.541580246	0.358221048	6.876281766	4.731888114
	-0.911296202	-4.198517071	2.418020177	-8.007346852	1.80491614
	2.63839778	4.325027358	2.063145135	4.358515356	31.46434026
	4.817829742	4.508499261	0.470189541	0.932557183	0.526687611
	2.385726722	2.798271214	1.898811571	1.511083861	1.600791819
	0.109953624	3.125526407	1.48041719	0.799545035	12.23363508
	1.375686661	9.118766556	1.873430676	11.70810602	4.925025958
	1.570540427	0.903998817	3.091721337	1.41939704	9.837089628
	1.336111948	4.652206548	0.655915527	6.244997622	2.557678298
	2.764219173	1.541580246	0.358221048	6.876281766	4.731888114
	0.911296202	4.198517071	2.418020177	8.007346852	1.80491614
**Mean**	1.989973587	3.90804372	1.5899858	4.650870081	7.742450323

REC_leap_z	LEO_leap_x	LEO_leap_y	LEO_leap_z	LEC_leap_x	LEC_leap_y
3.886976609	-4.23594358	5.026908972	-0.458995821	-7.054839754	25.72135924
-3.356838523	2.47929541	-4.158408964	0.600695619	9.193231601	15.69853957
4.993677488	-1.095086845	0.879554687	-0.048521043	-6.204535485	-3.272536934
3.049200551	-0.026941418	2.058593695	1.292668004	-10.28376031	1.243850443
-11.58222105	-1.709831438	0.21860506	-1.016199832	9.127169317	10.35533154
-0.396842609	-1.667217123	-1.564292848	-0.916862429	-12.18312312	-10.59302517
8.898932163	2.921498906	0.9235154	0.024158968	-1.608967611	9.994453505
-7.380346755	-0.311469927	-1.789306973	-2.183914193	3.978633416	-5.769401749
-5.735605955	-2.022653491	2.628326723	1.213085395	1.354966168	2.49715417
3.886976609	4.23594358	5.026908972	0.458995821	7.054839754	25.72135924
3.356838523	2.47929541	4.158408964	0.600695619	9.193231601	15.69853957
4.993677488	1.095086845	0.879554687	0.048521043	6.204535485	3.272536934
3.049200551	0.026941418	2.058593695	1.292668004	10.28376031	1.243850443
11.58222105	1.709831438	0.21860506	1.016199832	9.127169317	10.35533154
0.396842609	1.667217123	1.564292848	0.916862429	12.18312312	10.59302517
8.898932163	2.921498906	0.9235154	0.024158968	1.608967611	9.994453505
7.380346755	0.311469927	1.789306973	2.183914193	3.978633416	5.769401749
5.735605955	2.022653491	2.628326723	1.213085395	1.354966168	2.49715417
5.475626856	1.829993126	2.138612591	0.861677923	6.776580753	9.460628036

LEC_leap_z	REO_qtm_x	REO_qtm_y	REO_qtm_z	REC_qtm_x	REC_qtm_y
3.888601303	-0.237417952	-2.970480656	3.497491218	-3.156766361	-1.244179112
12.85161927	0.624561769	-4.509465336	2.364357943	-6.824527825	-0.662041858
-4.428223608	1.528570131	-1.94333923	5.473438477	-7.815385805	-4.090316355
-11.3955779	4.335690505	-3.373961715	1.975048539	-3.776114443	-4.63720442
-2.926255211	1.464871754	-2.917914318	4.64971405	7.727106284	10.49646097
-2.909039289	0.126294875	-3.966743265	3.092408252	13.15380339	3.639939424
4.075771135	3.027716282	-3.262917379	-0.816685195	0.77506507	-8.815237116
-3.557418388	1.315049108	-1.629729108	-1.049335883	7.04114555	5.554316869
-0.143018731	1.363221188	-3.145921151	-1.851381832	-2.972816739	6.388345275
3.888601303	0.237417952	2.970480656	3.497491218	3.156766361	1.244179112
12.85161927	0.624561769	4.509465336	2.364357943	6.824527825	0.662041858
4.428223608	1.528570131	1.94333923	5.473438477	7.815385805	4.090316355
11.3955779	4.335690505	3.373961715	1.975048539	3.776114443	4.63720442
2.926255211	1.464871754	2.917914318	4.64971405	7.727106284	10.49646097
2.909039289	0.126294875	3.966743265	3.092408252	13.15380339	3.639939424
4.075771135	3.027716282	3.262917379	0.816685195	0.77506507	8.815237116
3.557418388	1.315049108	1.629729108	1.049335883	7.04114555	5.554316869
0.143018731	1.363221188	3.145921151	1.851381832	2.972816739	6.388345275
5.130613871	1.55815484	3.080052462	2.752206821	5.915859052	5.058671266

REC_qtm_z	LEO_qtm_x	LEO_qtm_y	LEO_qtm_z	LEC_qtm_x	LEC_qtm_y
23.80306518	-3.852963918	2.761592992	1.807812661	-7.354430524	-6.392263117
25.40144309	-1.743268312	2.03518578	1.238707267	4.038906757	-18.81945037
25.81177305	-1.689871263	1.694550806	-2.768179461	-0.930373423	-14.81681114
34.42524453	-1.491985124	0.579147365	1.817008771	-7.413465649	-0.868025453
29.46802645	-2.304222975	1.634351439	2.247464635	-4.342656053	3.793114432
20.86823142	-3.172244382	1.841758307	0.260971694	-21.4354916	4.358362409
16.36608732	-1.534845086	0.683513299	0.98552056	-13.86821557	0.574109987
22.3065299	-1.968735745	2.092696966	-1.336479111	-14.46230664	8.204100492
20.52366555	-1.953020198	1.7291799	0.456318469	-11.04174522	5.991090242
23.80306518	3.852963918	2.761592992	1.807812661	7.354430524	6.392263117
25.40144309	1.743268312	2.03518578	1.238707267	4.038906757	18.81945037
25.81177305	1.689871263	1.694550806	2.768179461	0.930373423	14.81681114
34.42524453	1.491985124	0.579147365	1.817008771	7.413465649	0.868025453
29.46802645	2.304222975	1.634351439	2.247464635	4.342656053	3.793114432
20.86823142	3.172244382	1.841758307	0.260971694	21.4354916	4.358362409
16.36608732	1.534845086	0.683513299	0.98552056	13.86821557	0.574109987
22.3065299	1.968735745	2.092696966	1.336479111	14.46230664	8.204100492
20.52366555	1.953020198	1.7291799	0.456318469	11.04174522	5.991090242
24.33045183	2.190128556	1.672441873	1.435384736	9.431954604	7.090814183

LEC_qtm_z
20.10055781
35.80907128
35.74957485
36.6615779
42.47554875
35.24882277
45.14507354
38.49075551
35.51350402
20.10055781
35.80907128
35.74957485
36.6615779
42.47554875
35.24882277
45.14507354
38.49075551
35.51350402
36.13272072

Hand/axis	REO_leap_x	REO_leap_y	REO_leap_z	REC_leap_x	REC_leap_y
	-1.646156253	-6.425232209	-3.359864665	-29.60098708	9.17828365
	-3.417797167	-3.769844216	-2.308265326	4.457605723	1.461387543
	-4.890664077	-5.433639552	-1.066008485	41.93207237	-30.92089857
	-13.06898535	-5.155159394	-3.617131284	-39.75112775	21.31970254
	-2.101455007	-4.692077766	-1.226873921	-3.161028346	9.173080212
	-4.433819019	-3.903350973	-0.895371956	-0.992193082	9.656532388
	-3.339860353	-0.141410832	-0.054092634	2.231268239	-9.858879109
	-7.588710997	-5.638908746	-3.574768677	-9.78418172	-4.190407023
	-1.230762726	-5.449417277	-1.384272652	77.39946106	-25.62880707
	1.646156253	6.425232209	3.359864665	29.60098708	9.17828365
	3.417797167	3.769844216	2.308265326	4.457605723	1.461387543
	4.890664077	5.433639552	1.066008485	41.93207237	30.92089857
	13.06898535	5.155159394	3.617131284	39.75112775	21.31970254
	2.101455007	4.692077766	1.226873921	3.161028346	9.173080212
	4.433819019	3.903350973	0.895371956	0.992193082	9.656532388
	3.339860353	0.141410832	0.054092634	2.231268239	9.858879109
	7.588710997	5.638908746	3.574768677	9.78418172	4.190407023
	1.230762726	5.449417277	1.384272652	77.39946106	25.62880707
**Mean**	4.635356772	4.512115663	1.942961067	23.25665837	13.48755312

REC_leap_z	LEO_leap_x	LEO_leap_y	LEO_leap_z	LEC_leap_x	LEC_leap_y
-5.360938139	-9.775021694	1.344787593	0.469744435	15.03608347	8.9478378
-4.069807011	-1.67308083	2.22041299	-0.1712947	-9.036665246	-4.350349465
-52.39438256	-11.57217254	-34.95217122	-52.58497332	-0.901733404	0.508491375
50.45932427	26.43018839	27.5015948	52.53880937	12.74060228	-5.313506998
3.345147202	-38.32168046	-34.04737482	-41.36735011	-11.16575612	15.25363128
0.294070955	16.57931819	-8.432186262	38.96257298	9.132824487	-6.303901292
-2.171384072	-2.129602289	6.028930752	0.739211538	-6.035755294	9.732948144
-7.695363147	26.41518962	34.55744961	-1.315230659	15.02365264	-11.70709225
-39.40704239	-27.17840895	-20.60754443	2.785685353	-6.591216964	2.721229715
5.360938139	9.775021694	1.344787593	0.469744435	15.03608347	8.9478378
4.069807011	1.67308083	2.22041299	0.1712947	9.036665246	4.350349465
52.39438256	11.57217254	34.95217122	52.58497332	0.901733404	0.508491375
50.45932427	26.43018839	27.5015948	52.53880937	12.74060228	5.313506998
3.345147202	38.32168046	34.04737482	41.36735011	11.16575612	15.25363128
0.294070955	16.57931819	8.432186262	38.96257298	9.132824487	6.303901292
2.171384072	2.129602289	6.028930752	0.739211538	6.035755294	9.732948144
7.695363147	26.41518962	34.55744961	1.315230659	15.02365264	11.70709225
39.40704239	27.17840895	20.60754443	2.785685353	6.591216964	2.721229715
18.35527331	17.78607366	18.85471694	21.21498583	9.518254433	7.204332035

LEC_leap_z	REO_qtm_x	REO_qtm_y	REO_qtm_z	REC_qtm_x	REC_qtm_y	REC_qtm_z
-6.136874011	-1.441464866	2.051880216	-4.958110267	-24.59880469	4.653987628	11.71834596
-4.234189487	-0.872061399	2.659222963	-4.949911794	-24.23943364	8.06988555	11.3340159
-2.551969564	-3.753051083	4.78713412	-4.428107977	-26.0158793	7.650867739	8.605931865
2.070274869	-2.876222265	1.775420123	-3.578721139	-22.12341193	11.42370226	3.583941036
-1.024067916	-1.983135954	4.146865582	-6.856558362	-23.67844658	6.415727628	13.18494179
0.837528719	-2.714918273	2.673157273	-3.930198069	-21.37136892	5.099022089	22.29338419
-8.126037137	-2.02515965	2.406001179	-4.006518214	-21.12377745	8.850535933	11.63009626
3.932425947	-2.411574647	2.006653316	-4.255275114	-21.79643928	11.99265869	13.12682652
-8.217947427	-1.615634265	5.604116101	-5.621596524	-23.90448496	8.039976585	15.42302891
6.136874011	1.441464866	2.051880216	4.958110267	24.59880469	4.653987628	11.71834596
4.234189487	0.872061399	2.659222963	4.949911794	24.23943364	8.06988555	11.3340159
2.551969564	3.753051083	4.78713412	4.428107977	26.0158793	7.650867739	8.605931865
2.070274869	2.876222265	1.775420123	3.578721139	22.12341193	11.42370226	3.583941036
1.024067916	1.983135954	4.146865582	6.856558362	23.67844658	6.415727628	13.18494179
0.837528719	2.714918273	2.673157273	3.930198069	21.37136892	5.099022089	22.29338419
8.126037137	2.02515965	2.406001179	4.006518214	21.12377745	8.850535933	11.63009626
3.932425947	2.411574647	2.006653316	4.255275114	21.79643928	11.99265869	13.12682652
8.217947427	1.615634265	5.604116101	5.621596524	23.90448496	8.039976585	15.42302891
4.125701675	2.188135823	3.12338343	4.731666384	23.20578297	8.021818233	12.32227916

LEO_qtm_x	LEO_qtm_y	LEO_qtm_z	LEC_qtm_x	LEC_qtm_y	LEC_qtm_z
0.248154037	-1.03493286	0.827661548	11.29653058	10.37451149	12.26719196
1.600971918	-0.705069993	1.125648387	12.20820196	15.09736292	13.91577596
1.499098966	-3.409753545	0.95630611	7.20571798	17.3208275	13.90059455
2.272924567	-2.995135432	-0.3496434	15.15695961	16.51625677	16.3541113
0.638457169	-4.776647546	5.068389553	8.488773223	16.39481545	23.18614878
1.23679086	-2.530838406	2.011936298	12.45583459	15.48041982	22.44699859
-0.043562307	-2.739137765	3.550727762	14.54043055	24.561025	30.36930823
2.107164996	-2.411905234	2.167503017	21.50435841	21.4200839	24.32302986
1.488065712	-3.154212331	3.355288101	20.17875316	29.29712771	19.43257026
0.248154037	1.03493286	0.827661548	11.29653058	10.37451149	12.26719196
1.600971918	0.705069993	1.125648387	12.20820196	15.09736292	13.91577596
1.499098966	3.409753545	0.95630611	7.20571798	17.3208275	13.90059455
2.272924567	2.995135432	0.3496434	15.15695961	16.51625677	16.3541113
0.638457169	4.776647546	5.068389553	8.488773223	16.39481545	23.18614878
1.23679086	2.530838406	2.011936298	12.45583459	15.48041982	22.44699859
0.043562307	2.739137765	3.550727762	14.54043055	24.561025	30.36930823
2.107164996	2.411905234	2.167503017	21.50435841	21.4200839	24.32302986
1.488065712	3.154212331	3.355288101	20.17875316	29.29712771	19.43257026
1.237243392	2.639737012	2.157011575	13.67061779	18.49582562	19.57730328

Hand/axis	REO_leap_x	REO_leap_y	REO_leap_z	REC_leap_x	REC_leap_y
	-3.235542599	4.097297606	-1.180811358	-17.18757	44.09920507
	-0.022898827	-2.432082964	1.054052464	0.847276818	-2.700477829
	1.633064421	2.250335472	0.091372998	-10.94413071	0.63063049
	0.124757559	0.497138837	-1.7589965	27.33892524	-27.11985782
	-5.68418949	2.018928866	0.84914154	-29.62752437	26.28384448
	1.285899194	3.256317132	-4.200659114	-4.509557689	14.91136181
	1.503104121	-4.355476697	1.092134043	0.580520476	-1.894676549
	0.811871355	1.387145908	-3.010761982	11.5161939	9.253600942
	-1.202358832	-1.482047506	2.132071548	-10.48447829	-20.39928387
	3.235542599	4.097297606	1.180811358	17.18757	44.09920507
	0.022898827	2.432082964	1.054052464	0.847276818	2.700477829
	1.633064421	2.250335472	0.091372998	10.94413071	0.63063049
	0.124757559	0.497138837	1.7589965	27.33892524	27.11985782
	5.68418949	2.018928866	0.84914154	29.62752437	26.28384448
	1.285899194	3.256317132	4.200659114	4.509557689	14.91136181
	1.503104121	4.355476697	1.092134043	0.580520476	1.894676549
	0.811871355	1.387145908	3.010761982	11.5161939	9.253600942
	1.202358832	1.482047506	2.132071548	10.48447829	20.39928387
**Mean**	1.722631822	2.419641221	1.70777795	12.55957528	16.3658821

REC_leap_z	LEO_leap_x	LEO_leap_y	LEO_leap_z	LEC_leap_x	LEC_leap_y
28.3571577	-16.48487897	-43.06298845	-10.43105364	49.14733822	-6.787338361
-5.072791999	10.57086534	45.92714711	12.94989225	-31.04096985	-0.30512992
-5.96649818	-2.410269885	-1.923721286	0.715514832	-7.44579599	4.775321935
-31.19865241	-3.19011222	3.762809573	1.957884189	-35.32957501	-49.55577836
25.97201921	7.533005605	-4.15949982	-0.924338696	-16.12528209	12.1314774
-2.427333072	-2.758351644	-1.420052055	-1.243634222	64.38498036	68.09430665
-5.175471356	-1.617634364	4.58425171	-0.88452188	8.685193731	1.066757361
-4.923343582	10.42198765	1.173065118	1.27918928	-11.51682644	4.742156821
-3.167989844	-7.809461991	4.655944837	3.662073015	1.459943498	11.85723134
28.3571577	16.48487897	43.06298845	10.43105364	49.14733822	6.787338361
5.072791999	10.57086534	45.92714711	12.94989225	31.04096985	0.30512992
5.96649818	2.410269885	1.923721286	0.715514832	7.44579599	4.775321935
31.19865241	3.19011222	3.762809573	1.957884189	35.32957501	49.55577836
25.97201921	7.533005605	4.15949982	0.924338696	16.12528209	12.1314774
2.427333072	2.758351644	1.420052055	1.243634222	64.38498036	68.09430665
5.175471356	1.617634364	4.58425171	0.88452188	8.685193731	1.066757361
4.923343582	10.42198765	1.173065118	1.27918928	11.51682644	4.742156821
3.167989844	7.809461991	4.655944837	3.662073015	1.459943498	11.85723134
12.47347304	6.977396407	12.29660888	3.783122445	25.01510058	17.70172202

LEC_leap_z	REO_qtm_x	REO_qtm_y	REO_qtm_z	REC_qtm_x	REC_qtm_y
-9.269855064	-2.189605106	-0.067551981	3.466590234	2.732440338	12.14202783
-3.218347917	-0.730388104	-0.196711029	1.139049404	9.583251227	15.89925766
-15.04859259	-1.164661153	-0.716384584	1.896796483	5.005832378	27.84589599
-54.92381288	-0.985007784	0.29597674	2.610378144	-0.696275715	26.08338194
1.495403367	-3.291864717	-0.608157266	4.545978578	0.436815447	32.42434169
53.74699585	-3.005571769	1.889963185	5.158344206	-2.745226155	39.31714093
3.090619062	-0.498230282	1.603432275	2.09374887	-4.890926534	40.62796565
-13.06320281	-1.874605053	2.911692831	3.592110193	4.628651558	44.04153393
2.861324361	-1.389977603	1.997500344	3.137162238	-3.748959664	49.07954389
9.269855064	2.189605106	0.067551981	3.466590234	2.732440338	12.14202783
3.218347917	0.730388104	0.196711029	1.139049404	9.583251227	15.89925766
15.04859259	1.164661153	0.716384584	1.896796483	5.005832378	27.84589599
54.92381288	0.985007784	0.29597674	2.610378144	0.696275715	26.08338194
1.495403367	3.291864717	0.608157266	4.545978578	0.436815447	32.42434169
53.74699585	3.005571769	1.889963185	5.158344206	2.745226155	39.31714093
3.090619062	0.498230282	1.603432275	2.09374887	4.890926534	40.62796565
13.06320281	1.874605053	2.911692831	3.592110193	4.628651558	44.04153393
2.861324361	1.389977603	1.997500344	3.137162238	3.748959664	49.07954389
17.41312821	1.681101286	1.143041137	3.071128705	3.829819891	31.94012106

REC_qtm_z	LEO_qtm_x	LEO_qtm_y	LEO_qtm_z	LEC_qtm_x	LEC_qtm_y
14.71924998	-0.225161177	-1.467218904	0.261310144	35.54211499	8.622762094
22.47512911	-1.213082977	-0.483854777	0.307535146	27.56167449	17.59892377
32.89128077	-1.202367776	-0.174947919	1.021360242	19.19327261	32.88895846
26.37263999	-1.075547061	-1.737714281	1.383862277	32.79362254	26.48270355
25.51304764	0.601922245	-2.282764451	1.035291946	26.59198602	34.50680228
47.61087852	-1.425541637	-0.288825896	-0.196310624	39.32368359	28.19163886
36.49932948	-1.555501669	-1.54687163	0.406630243	44.11023622	26.21695777
42.42578571	-1.384294749	-0.982862665	1.533930313	31.2477573	38.43933148
26.32048896	-1.818048907	-4.039339374	1.358916111	37.35091087	39.03993374
14.71924998	0.225161177	1.467218904	0.261310144	35.54211499	8.622762094
22.47512911	1.213082977	0.483854777	0.307535146	27.56167449	17.59892377
32.89128077	1.202367776	0.174947919	1.021360242	19.19327261	32.88895846
26.37263999	1.075547061	1.737714281	1.383862277	32.79362254	26.48270355
25.51304764	0.601922245	2.282764451	1.035291946	26.59198602	34.50680228
47.61087852	1.425541637	0.288825896	0.196310624	39.32368359	28.19163886
36.49932948	1.555501669	1.54687163	0.406630243	44.11023622	26.21695777
42.42578571	1.384294749	0.982862665	1.533930313	31.2477573	38.43933148
26.32048896	1.818048907	4.039339374	1.358916111	37.35091087	39.03993374
30.53642557	1.1668298	1.444933322	0.833905227	32.63502874	27.998668

LEC_qtm_z
-5.20148902
-3.276987672
0.127712373
9.22094929
21.3886696
26.35825479
32.41546549
33.44133078
47.17909984
5.20148902
3.276987672
0.127712373
9.22094929
21.3886696
26.35825479
32.41546549
33.44133078
47.17909984
19.84555098

Hand/axis	REO_leap_x	REO_leap_y	REO_leap_z	REC_leap_x	REC_leap_y	REC_leap_z
	0.720900077	-2.184852659	1.648230734	-1.275694912	5.755470322	1.043776783
	2.779241342	1.264831583	2.061533019	-2.799446229	12.45754252	2.392583055
	1.88698182	4.299354641	2.98400527	-7.552823126	12.51979082	-3.460703578
	2.54383253	1.420349161	2.884477297	0.698267061	11.84030931	-10.38984426
	-0.682126948	1.464645182	1.580880013	-7.002508706	14.83404553	-8.216458908
	-3.864577907	-0.831734118	0.714360507	-5.441097073	16.68179371	-11.00116166
	-6.499618774	-4.440719652	-0.671027062	11.84037111	31.4511264	-18.61140212
	4.637781728	3.676849253	3.913596931	7.76063625	10.17871854	-26.61601586
	-4.624836033	-4.387268011	0.157072017	-7.808227797	17.53365313	-17.45219763
	0.720900077	2.184852659	1.648230734	1.275694912	5.755470322	1.043776783
	2.779241342	1.264831583	2.061533019	2.799446229	12.45754252	2.392583055
	1.88698182	4.299354641	2.98400527	7.552823126	12.51979082	3.460703578
	2.54383253	1.420349161	2.884477297	0.698267061	11.84030931	10.38984426
	0.682126948	1.464645182	1.580880013	7.002508706	14.83404553	8.216458908
	3.864577907	0.831734118	0.714360507	5.441097073	16.68179371	11.00116166
	6.499618774	4.440719652	0.671027062	11.84037111	31.4511264	18.61140212
	4.637781728	3.676849253	3.913596931	7.76063625	10.17871854	26.61601586
	4.624836033	4.387268011	0.157072017	7.808227797	17.53365313	17.45219763
**Mean**	3.137766351	2.663400473	1.846131428	5.797674696	14.80582781	11.02046043

LEO_leap_x	LEO_leap_y	LEO_leap_z	LEC_leap_x	LEC_leap_y	LEC_leap_z
9.614359375	-8.255996558	-2.020016979	-0.021043779	-7.614456191	9.40039031
1.014135112	1.034721332	2.345547926	-7.647768664	-7.422043114	5.594860799
7.996093195	-5.6015618	0.893662364	-20.77554276	5.603157353	2.076369145
7.866335396	-3.421981855	3.997582714	-37.75776699	-3.76007838	-11.73764287
1.332770865	3.969139226	4.861005982	-13.87622461	-1.535575754	10.06857481
4.193865811	-5.705360396	2.740208576	-5.586754192	-1.286186001	10.67185305
4.034636812	2.772201452	4.667275266	-8.963411383	-7.409850673	7.26973512
7.286680035	-4.584480189	1.88903471	-3.292355318	-9.309547586	4.630067282
-1.75205082	3.748543026	2.283628953	-1.954775555	-0.211707767	7.900888917
9.614359375	8.255996558	2.020016979	0.021043779	7.614456191	9.40039031
1.014135112	1.034721332	2.345547926	7.647768664	7.422043114	5.594860799
7.996093195	5.6015618	0.893662364	20.77554276	5.603157353	2.076369145
7.866335396	3.421981855	3.997582714	37.75776699	3.76007838	11.73764287
1.332770865	3.969139226	4.861005982	13.87622461	1.535575754	10.06857481
4.193865811	5.705360396	2.740208576	5.586754192	1.286186001	10.67185305
4.034636812	2.772201452	4.667275266	8.963411383	7.409850673	7.26973512
7.286680035	4.584480189	1.88903471	3.292355318	9.309547586	4.630067282
1.752050823	3.748543026	2.283628953	1.954775555	0.211707767	7.900888917
5.010103047	4.343776204	2.855329274	11.0972937	4.905844758	7.705598033

REO_qtm_x	REO_qtm_y	REO_qtm_z	REC_qtm_x	REC_qtm_y	REC_qtm_z
-1.176112091	-1.516161879	-0.234727564	3.515157711	-5.915538636	10.43799075
-0.072305526	-0.649082339	0.233212479	-4.987424387	-5.787331559	12.91559326
1.748351915	0.019029958	-0.435219596	-7.110770272	3.597387732	9.865069065
-0.587896141	-1.614548248	-0.170550027	-0.568236256	8.88429363	10.52537326
-0.241959044	-0.407416627	0.242077254	-6.71014613	5.694590748	11.86522934
1.322382282	-0.734992299	-0.160408768	-4.700988303	10.90908994	13.7735407
-0.455099466	-0.245834376	0.215649666	-2.673293568	15.05891353	15.644125
1.012751698	-1.732102904	0.044623722	-0.021686811	20.29140137	13.40840203
0.357075327	-2.045643163	1.142673616	-8.191151454	15.43867996	12.15334915
1.176112091	1.516161879	0.234727564	3.515157711	5.915538636	10.43799075
0.072305526	0.649082339	0.233212479	4.987424387	5.787331559	12.91559326
1.748351915	0.019029958	0.435219596	7.110770272	3.597387732	9.865069065
0.587896141	1.614548248	0.170550027	0.568236256	8.88429363	10.52537326
0.241959044	0.407416627	0.242077254	6.71014613	5.694590748	11.86522934
1.322382282	0.734992299	0.160408768	4.700988303	10.90908994	13.7735407
0.455099466	0.245834376	0.215649666	2.673293568	15.05891353	15.644125
1.012751698	1.732102904	0.044623722	0.021686811	20.29140137	13.40840203
0.357075327	2.045643163	1.142673616	8.191151454	15.43867996	12.15334915
0.774881499	0.996090199	0.319904743	4.275428321	10.17524746	12.28763028

LEO_qtm_x	LEO_qtm_y	LEO_qtm_z	LEC_qtm_x	LEC_qtm_y	LEC_qtm_z
2.535722613	-0.897249335	1.576390138	5.436303327	-2.372384194	-3.56707178
0.212156081	0.731383342	0.160183282	-1.607173668	-2.572398798	-3.07035444
1.127479212	1.987226881	-0.203591735	-3.735347647	2.173922118	-2.362702727
1.89891927	-1.52151873	1.160200257	-14.69748546	8.244930739	-0.643497514
0.757432955	0.30805445	0.532576899	-2.227782808	-3.370924229	-6.628829394
-1.792450858	-0.364532358	1.484379784	0.14459727	-5.173079332	-1.051161766
1.822367413	0.961116013	-2.521047125	-5.856771341	-2.923024352	-2.980897905
-0.49564791	0.725301653	-0.756534927	3.756456167	-5.483271024	-4.201369701
1.011469136	0.543535413	-0.054185078	6.18063062	-4.218552628	-1.812935084
2.535722613	0.897249335	1.576390138	5.436303327	2.372384194	3.56707178
0.212156081	0.731383342	0.160183282	1.607173668	2.572398798	3.07035444
1.127479212	1.987226881	0.203591735	3.735347647	2.173922118	2.362702727
1.89891927	1.52151873	1.160200257	14.69748546	8.244930739	0.643497514
0.757432955	0.30805445	0.532576899	2.227782808	3.370924229	6.628829394
1.792450858	0.364532358	1.484379784	0.14459727	5.173079332	1.051161766
1.822367413	0.961116013	2.521047125	5.856771341	2.923024352	2.980897905
0.49564791	0.725301653	0.756534927	3.756456167	5.483271024	4.201369701
1.011469136	0.543535413	0.054185078	6.18063062	4.218552628	1.812935084
1.294849494	0.893324241	0.938787692	4.849172034	4.059165268	2.924313368

Hand/axis	REO_leap_x	REO_leap_y	REO_leap_z	REC_leap_x	REC_leap_y	REC_leap_z
	4.965433277	6.703659193	-3.638376817	-8.42151401	40.99644469	-22.9113817
	-3.228890286	1.811416789	0.820400101	-13.05025752	47.51921068	-28.7530518
	-0.398306722	0.87297806	-0.722395314	-4.318945001	73.06924474	-29.31425845
	2.497938246	5.463417105	1.277169855	-7.579802178	66.06117237	-36.01443585
	5.029310059	4.303871088	-0.526359787	5.894934252	67.01741797	-30.9503775
	0.93453074	4.172699257	0.718947932	-3.211261182	57.31883256	-45.91764347
	2.907036512	4.655601391	0.073572654	-0.971489684	45.82102205	-49.67544664
	2.516593151	5.657554619	-1.557894581	7.781081334	69.21672836	-52.97770005
	-0.421116885	3.755401587	-0.443223139	0.937624919	39.91328413	-48.04852019
	4.965433277	6.703659193	3.638376817	8.42151401	40.99644469	22.9113817
	3.228890286	1.811416789	0.820400101	13.05025752	47.51921068	28.7530518
	0.398306722	0.87297806	0.722395314	4.318945001	73.06924474	29.31425845
	2.497938246	5.463417105	1.277169855	7.579802178	66.06117237	36.01443585
	5.029310059	4.303871088	0.526359787	5.894934252	67.01741797	30.9503775
	0.93453074	4.172699257	0.718947932	3.211261182	57.31883256	45.91764347
	2.907036512	4.655601391	0.073572654	0.971489684	45.82102205	49.67544664
	2.516593151	5.657554619	1.557894581	7.781081334	69.21672836	52.97770005
	0.421116885	3.755401587	0.443223139	0.937624919	39.91328413	48.04852019
**Mean**	2.544350653	4.155177677	1.086482242	5.796323343	56.32592862	38.28475729

LEO_leap_x	LEO_leap_y	LEO_leap_z	LEC_leap_x	LEC_leap_y	LEC_leap_z
-6.161739419	2.909492357	-5.438960168	30.63204887	2.468498271	-3.435738676
-0.509612706	0.109459102	2.510107749	38.40366765	0.461631802	1.339321592
2.805182898	-1.186157409	0.120683195	42.37693217	15.49045563	-15.18725119
1.916569667	-2.471870382	-0.910163629	43.02167356	-4.845596346	-14.8251592
1.165177922	4.733268972	1.72777628	48.39461516	0.583503707	-23.88772592
-0.39487831	5.611778116	-0.330471533	52.96279766	27.38840467	-10.92682992
-3.933314092	-7.276481805	-2.449231128	53.48707005	17.80149849	-26.55435096
-0.434567913	-4.056960772	1.590890397	61.99227004	35.74555961	-22.79250158
4.082664956	2.310676343	1.745397466	69.96945606	45.62201681	-36.00436129
6.161739419	2.909492357	5.438960168	30.63204887	2.468498271	3.435738676
0.509612706	0.109459102	2.510107749	38.40366765	0.461631802	1.339321592
2.805182898	1.186157409	0.120683195	42.37693217	15.49045563	15.18725119
1.916569667	2.471870382	0.910163629	43.02167356	4.845596346	14.8251592
1.165177922	4.733268972	1.72777628	48.39461516	0.583503707	23.88772592
0.39487831	5.611778116	0.330471533	52.96279766	27.38840467	10.92682992
3.933314092	7.276481805	2.449231128	53.48707005	17.80149849	26.55435096
0.434567913	4.056960772	1.590890397	61.99227004	35.74555961	22.79250158
4.082664956	2.310676343	1.745397466	69.96945606	45.62201681	36.00436129
2.378189765	3.407349473	1.86929795	49.02672569	16.71190726	17.2170267

REO_qtm_x	REO_qtm_y	REO_qtm_z	REC_qtm_x	REC_qtm_y	REC_qtm_z
-0.642104529	2.350646093	-1.983080226	-1.34772028	17.60185899	47.71324372
-0.968020765	-0.484173941	-0.16949182	-15.22684798	28.91571375	47.54651739
0.115219736	0.101487132	-1.915510686	-4.909076025	27.65937177	71.06557596
-1.411677076	-0.161338398	0.082036496	-3.455553115	37.22978455	64.30297522
-0.937072245	0.034030641	-0.399645353	57.49537718	43.90399929	36.04841481
0.034425597	-0.315761763	-0.223472466	36.7565625	47.39431273	37.7011206
-0.395027641	0.55643899	-0.324526762	0.71506039	52.74963541	47.04817874
0.351625658	0.833022473	-1.907969422	9.604045541	55.31809171	51.53881109
-1.04686393	1.07975099	-2.913981945	3.021540272	52.12368082	42.54070204
0.642104529	2.350646093	1.983080226	1.34772028	17.60185899	47.71324372
0.968020765	0.484173941	0.16949182	15.22684798	28.91571375	47.54651739
0.115219736	0.101487132	1.915510686	4.909076025	27.65937177	71.06557596
1.411677076	0.161338398	0.082036496	3.455553115	37.22978455	64.30297522
0.937072245	0.034030641	0.399645353	57.49537718	43.90399929	36.04841481
0.034425597	0.315761763	0.223472466	36.7565625	47.39431273	37.7011206
0.395027641	0.55643899	0.324526762	0.71506039	52.74963541	47.04817874
0.351625658	0.833022473	1.907969422	9.604045541	55.31809171	51.53881109
1.04686393	1.07975099	2.913981945	3.021540272	52.12368082	42.54070204
0.655781909	0.657405602	1.102190575	14.7257537	40.32182767	49.50061551

LEO_qtm_x	LEO_qtm_y	LEO_qtm_z	LEC_qtm_x	LEC_qtm_y	LEC_qtm_z
1.127523872	4.177039384	2.14736879	33.43286738	4.992172287	4.916174199
-2.56949195	1.961573393	-1.153315843	43.5499248	4.039545395	3.228447205
0.675002667	1.17331469	-2.705483656	48.12008659	16.21120854	17.70265185
-0.897925386	3.147554564	-3.282888297	48.93475722	18.33702456	-0.842825066
0.75395867	1.816101861	-0.964565228	55.59145242	26.24130056	3.906541641
1.149486129	0.264226713	4.993667528	58.94781543	10.76402295	25.13162284
0.319830457	3.134169363	-1.505620246	59.71139277	29.48151665	20.993247
0.47951952	2.145014308	-0.558846974	69.64356044	26.11436996	33.65522271
-1.388522223	1.436905602	-0.513967022	79.36268098	42.34798817	44.95140451
1.127523872	4.177039384	2.14736879	33.43286738	4.992172287	4.916174199
2.56949195	1.961573393	1.153315843	43.5499248	4.039545395	3.228447205
0.675002667	1.17331469	2.705483656	48.12008659	16.21120854	17.70265185
0.897925386	3.147554564	3.282888297	48.93475722	18.33702456	0.842825066
0.75395867	1.816101861	0.964565228	55.59145242	26.24130056	3.906541641
1.149486129	0.264226713	4.993667528	58.94781543	10.76402295	25.13162284
0.319830457	3.134169363	1.505620246	59.71139277	29.48151665	20.993247
0.47951952	2.145014308	0.558846974	69.64356044	26.11436996	33.65522271
1.388522223	1.436905602	0.513967022	79.36268098	42.34798817	44.95140451
1.040140097	2.139544431	1.980635954	55.25494867	19.83657212	17.25868189

**Hand/axis**	REO_leap_x	REO_leap_y	REO_leap_z	REC_leap_x	REC_leap_y	REC_leap_z
	0.78470701	1.743667325	-2.296961662	-10.24945556	20.66352833	-30.95931016
	-2.499416482	-2.667244006	-3.234478018	-19.43818392	34.06399517	-45.19732145
	0.235275588	-2.125335691	-2.422646591	-17.10875416	43.51840964	-53.72629797
	-1.306559927	-0.640472411	-2.424514993	-29.34995561	42.48073569	-51.32609046
	-0.016713944	-1.978713779	0.371777848	-25.1155576	22.813026	-59.09191387
	2.182522847	1.17491117	-2.285352479	-26.89083784	20.46150968	-73.97488083
	-3.163499911	1.33677637	-1.904723822	-25.18684251	32.92883307	-76.94242132
	-2.052678097	-0.890991415	-1.898755044	-24.69487093	11.81285856	-79.115868
	-0.415141125	1.180289915	-2.72804505	-32.87075161	22.28835282	-75.89205998
	0.78470701	1.743667325	2.296961662	10.24945556	20.66352833	30.95931016
	2.499416482	2.667244006	3.234478018	19.43818392	34.06399517	45.19732145
	0.235275588	2.125335691	2.422646591	17.10875416	43.51840964	53.72629797
	1.306559927	0.640472411	2.424514993	29.34995561	42.48073569	51.32609046
	0.016713944	1.978713779	0.371777848	25.1155576	22.813026	59.09191387
	2.182522847	1.17491117	2.285352479	26.89083784	20.46150968	73.97488083
	3.163499911	1.33677637	1.904723822	25.18684251	32.92883307	76.94242132
	2.052678097	0.890991415	1.898755044	24.69487093	11.81285856	79.115868
	0.415141125	1.180289915	2.72804505	32.87075161	22.28835282	75.89205998
**Mean**	1.406279437	1.52648912	2.174139501	23.43391219	27.892361	60.691796

LEO_leap_x	LEO_leap_y	LEO_leap_z	LEC_leap_x	LEC_leap_y	LEC_leap_z
1.635984342	2.848854149	0.741593559	11.88154334	6.862251461	-13.63598741
3.437897152	1.083694572	-0.445199512	7.789606321	4.134376465	-31.02771249
0.908557409	0.681709369	0.297567164	26.55789562	14.85141775	-13.78396397
0.118314209	-1.975975132	0.595013709	28.87543296	5.31266804	-28.64318139
-0.613678643	-1.653457414	3.045527084	52.76188094	8.318924153	-18.08554274
-1.05642524	2.585411068	2.185568263	39.74948138	9.429786969	-47.9462162
1.178676258	2.559379595	-1.366243836	53.8644504	19.85160087	-60.08074721
-0.075098247	0.271812299	-2.764293311	54.51697325	19.3621674	-57.98928223
0.545186792	0.074257018	-1.109199411	49.30451758	6.371978947	-80.82947685
1.635984342	2.848854149	0.741593559	11.88154334	6.862251461	13.63598741
3.437897152	1.083694572	0.445199512	7.789606321	4.134376465	31.02771249
0.908557409	0.681709369	0.297567164	26.55789562	14.85141775	13.78396397
0.118314209	1.975975132	0.595013709	28.87543296	5.31266804	28.64318139
0.613678643	1.653457414	3.045527084	52.76188094	8.318924153	18.08554274
1.05642524	2.585411068	2.185568263	39.74948138	9.429786969	47.9462162
1.178676258	2.559379595	1.366243836	53.8644504	19.85160087	60.08074721
0.075098247	0.271812299	2.764293311	54.51697325	19.3621674	57.98928223
0.545186792	0.074257018	1.109199411	49.30451758	6.371978947	80.82947685
1.063313144	1.52606118	1.394467316	36.14464242	10.49946356	39.11356783

REO_qtm_x	REO_qtm_y	REO_qtm_z	REC_qtm_x	REC_qtm_y	REC_qtm_z
0.40810173	0.430176158	-0.25375653	-8.087312416	33.25309639	21.7166559
-0.598697613	-0.043038959	0.50584539	-20.59213282	47.60413553	34.91227451
1.415362114	2.217981118	-2.205063004	-17.60344318	57.80568395	42.53099408
0.207572648	1.341504794	-2.31783185	-29.06804879	54.56038031	41.3155089
0.444981872	-0.083089818	-0.006171514	-21.14325185	61.48324631	24.89770683
0.942140176	1.638913424	-1.891419201	-27.87525436	78.48248848	19.56153301
0.81881075	0.631661716	-1.495056435	-26.94601216	81.37044735	34.58314867
0.037807564	0.746116871	-2.42547142	-28.07297826	84.08271478	14.58353318
0.116115357	1.073320652	-1.463980597	-36.00732266	79.05292189	22.27640443
0.40810173	0.430176158	0.25375653	8.087312416	33.25309639	21.7166559
0.598697613	0.043038959	0.50584539	20.59213282	47.60413553	34.91227451
1.415362114	2.217981118	2.205063004	17.60344318	57.80568395	42.53099408
0.207572648	1.341504794	2.31783185	29.06804879	54.56038031	41.3155089
0.444981872	0.083089818	0.006171514	21.14325185	61.48324631	24.89770683
0.942140176	1.638913424	1.891419201	27.87525436	78.48248848	19.56153301
0.81881075	0.631661716	1.495056435	26.94601216	81.37044735	34.58314867
0.037807564	0.746116871	2.42547142	28.07297826	84.08271478	14.58353318
0.116115357	1.073320652	1.463980597	36.00732266	79.05292189	22.27640443
0.554398869	0.911755945	1.396066216	23.93286183	64.18834611	28.48641772

LEO_qtm_x	LEO_qtm_y	LEO_qtm_z	LEC_qtm_x	LEC_qtm_y	LEC_qtm_z
0.024403975	-1.369093496	-0.214667903	5.766096075	15.5466493	12.03325188
0.224802445	0.470234974	-1.211774724	3.349056925	34.70864657	0.39050005
-1.426592341	-0.621616997	-1.112261622	23.48152373	10.12638952	13.90775813
-1.785423695	-0.878887015	-1.875094158	26.577403	27.00154326	5.204437487
0.491321988	-0.390201903	-1.206432095	51.32076432	19.23578873	8.469593563
-0.715148314	-1.308401832	0.782876802	40.17114314	47.28004691	6.992350062
-0.238047305	0.764057216	-1.666025618	51.80460709	62.29217024	19.0576646
-1.310746571	1.633225204	-1.97207067	56.24650081	60.16956431	19.84610781
-0.371474863	-0.661833639	-2.083014719	56.24544094	82.93890586	9.536128478
0.024403975	1.369093496	0.214667903	5.766096075	15.5466493	12.03325188
0.224802445	0.470234974	1.211774724	3.349056925	34.70864657	0.39050005
1.426592341	0.621616997	1.112261622	23.48152373	10.12638952	13.90775813
1.785423695	0.878887015	1.875094158	26.577403	27.00154326	5.204437487
0.491321988	0.390201903	1.206432095	51.32076432	19.23578873	8.469593563
0.715148314	1.308401832	0.782876802	40.17114314	47.28004691	6.992350062
0.238047305	0.764057216	1.666025618	51.80460709	62.29217024	19.0576646
1.310746571	1.633225204	1.97207067	56.24650081	60.16956431	19.84610781
0.371474863	0.661833639	2.083014719	56.24544094	82.93890586	9.536128478
0.731995722	0.899728031	1.347135368	34.99583734	39.92218941	10.60419912

**Hand/axis**	REO_leap_x	REO_leap_y	REO_leap_z	REC_leap_x	REC_leap_y	REC_leap_z
	-0.959535409	3.322456368	-3.155739309	7.651910225	9.411155819	-8.552398113
	3.346777961	0.737091061	-2.116708739	6.837343459	29.9567032	-13.08782779
	2.499103783	-1.061630201	-0.02491329	17.76392091	36.45930476	-15.90054345
	2.60366898	0.141639641	-0.129592381	17.45569713	31.00273886	-20.53368615
	2.10807227	-0.25453945	-0.298603704	23.86937632	21.2922439	-26.0484603
	-2.587922185	1.860244584	-4.660476545	15.09220097	13.40410617	-26.60332782
	1.545653369	1.997238221	-1.450642854	26.21039513	30.39961975	-29.89549874
	2.296641411	0.268821772	-0.96329442	23.38321592	43.18901807	-21.57444884
	2.130292962	0.575180053	0.234087762	20.61348409	35.86033614	-27.28376127
	0.959535409	3.322456368	3.155739309	7.651910225	9.411155819	8.552398113
	3.346777961	0.737091061	2.116708739	6.837343459	29.9567032	13.08782779
	2.499103783	1.061630201	0.02491329	17.76392091	36.45930476	15.90054345
	2.60366898	0.141639641	0.129592381	17.45569713	31.00273886	20.53368615
	2.10807227	0.25453945	0.298603704	23.86937632	21.2922439	26.0484603
	2.587922185	1.860244584	4.660476545	15.09220097	13.40410617	26.60332782
	1.545653369	1.997238221	1.450642854	26.21039513	30.39961975	29.89549874
	2.296641411	0.268821772	0.96329442	23.38321592	43.18901807	21.57444884
	2.130292962	0.575180053	0.234087762	20.61348409	35.86033614	27.28376127
**Mean**	2.230852037	1.135426817	1.448228778	17.65306046	27.8861363	21.05332805

LEO_leap_x	LEO_leap_y	LEO_leap_z	LEC_leap_x	LEC_leap_y	LEC_leap_z
-1.233070461	1.397583025	-2.924706634	-1.620190366	10.7703857	-5.421333693
-0.956577913	-2.8002777	-1.90011121	-8.364114438	19.53707882	-3.698351699
0.082509861	2.162429828	-1.691870847	-10.85133848	22.45771807	-9.10522602
0.739723064	1.623573288	-3.231026705	-24.79547679	14.19376361	-13.19906553
3.054270444	-2.569877883	-3.359009817	-0.638626592	18.1502609	-7.350624711
-1.540508186	1.635864321	-3.801016139	-13.36022686	22.54434955	-14.5285637
-0.808749397	1.62284095	-3.005660907	-14.02673321	26.97521981	-9.541072765
-0.301248775	0.431915327	-2.269343704	-16.21885671	11.23158272	-16.73641777
0.143093162	-0.992759737	-3.381444829	-13.68603806	13.31785569	-21.08793002
1.233070461	1.397583025	2.924706634	1.620190366	10.7703857	5.421333693
0.956577913	2.8002777	1.90011121	8.364114438	19.53707882	3.698351699
0.082509861	2.162429828	1.691870847	10.85133848	22.45771807	9.10522602
0.739723064	1.623573288	3.231026705	24.79547679	14.19376361	13.19906553
3.054270444	2.569877883	3.359009817	0.638626592	18.1502609	7.350624711
1.540508186	1.635864321	3.801016139	13.36022686	22.54434955	14.5285637
0.808749397	1.62284095	3.005660907	14.02673321	26.97521981	9.541072765
0.301248775	0.431915327	2.269343704	16.21885671	11.23158272	16.73641777
0.143093162	0.992759737	3.381444829	13.68603806	13.31785569	21.08793002
0.984416807	1.693013562	2.840465644	11.50684461	17.68646832	11.18539843

REO_qtm_x	REO_qtm_y	REO_qtm_z	REC_qtm_x	REC_qtm_y	REC_qtm_z
0.002691082	0.571824126	1.120739499	11.16384163	9.864793516	10.029439
1.108985513	0.459912287	-0.329941598	10.61843339	13.16231663	23.32629621
1.499527108	0.299022026	-0.763932116	12.67598269	16.59314597	29.11660568
0.412947445	0.21399345	-0.675547154	19.92265878	23.42768336	22.80795046
1.533603131	0.218989317	-0.273997047	21.49568876	24.39779462	17.0754503
1.371029226	1.258200014	0.025326566	20.02919555	25.89872155	12.97973927
1.460232905	0.784658576	-0.523972964	22.11956149	26.26141663	33.7679642
1.339666659	0.126972115	-0.336543016	23.52710064	22.58896188	39.54246856
1.906291182	-0.164999283	-0.024002675	19.97618212	27.1542998	31.5326891
0.002691082	0.571824126	1.120739499	11.16384163	9.864793516	10.029439
1.108985513	0.459912287	0.329941598	10.61843339	13.16231663	23.32629621
1.499527108	0.299022026	0.763932116	12.67598269	16.59314597	29.11660568
0.412947445	0.21399345	0.675547154	19.92265878	23.42768336	22.80795046
1.533603131	0.218989317	0.273997047	21.49568876	24.39779462	17.0754503
1.371029226	1.258200014	0.025326566	20.02919555	25.89872155	12.97973927
1.460232905	0.784658576	0.523972964	22.11956149	26.26141663	33.7679642
1.339666659	0.126972115	0.336543016	23.52710064	22.58896188	39.54246856
1.906291182	0.164999283	0.024002675	19.97618212	27.1542998	31.5326891
1.181663806	0.455396799	0.45266696	17.94762723	21.03879266	24.4642892

LEO_qtm_x	LEO_qtm_y	LEO_qtm_z	LEC_qtm_x	LEC_qtm_y	LEC_qtm_z
-1.145881348	1.861926613	1.716542525	1.216954608	7.521557027	7.320623697
-1.695755728	0.866669222	-1.63352628	-3.814122509	7.336543103	17.17364281
-0.216250587	1.555477888	2.708384658	-6.891178381	11.46385165	17.16881342
-0.821310935	2.713629362	2.491165653	-7.462810972	14.34760676	5.670706456
-1.594550252	-0.738010004	2.697924512	0.28141082	14.10037215	16.5960852
-1.690602959	0.591777903	2.553379532	-10.22106071	18.05221243	21.37446153
-2.267226742	0.894557073	2.895622448	-9.43792875	14.4168822	22.22120423
-1.326598445	-1.560405118	2.49102082	-12.35847016	19.8890824	13.87097466
-0.882213807	0.236212392	3.179766886	-13.63259001	26.22868376	10.98324206
1.145881348	1.861926613	1.716542525	1.216954608	7.521557027	7.320623697
1.695755728	0.866669222	1.63352628	3.814122509	7.336543103	17.17364281
0.216250587	1.555477888	2.708384658	6.891178381	11.46385165	17.16881342
0.821310935	2.713629362	2.491165653	7.462810972	14.34760676	5.670706456
1.594550252	0.738010004	2.697924512	0.28141082	14.10037215	16.5960852
1.690602959	0.591777903	2.553379532	10.22106071	18.05221243	21.37446153
2.267226742	0.894557073	2.895622448	9.43792875	14.4168822	22.22120423
1.326598445	1.560405118	2.49102082	12.35847016	19.8890824	13.87097466
0.882213807	0.236212392	3.179766886	13.63259001	26.22868376	10.98324206
1.293376756	1.224296175	2.485259257	7.257391879	14.81742127	14.70886156

**Hand/axis**	REO_leap_x	REO_leap_y	REO_leap_z	REC_leap_x	REC_leap_y	REC_leap_z
	-1.425850018	-1.184799073	-0.609476823	-9.069868166	8.404899642	0.846522075
	-2.094695217	-1.739700176	-2.610227531	-8.836160356	11.59165196	-2.718847895
	-1.377496985	1.048736443	-1.089223858	-21.01340385	12.39928438	5.530042159
	-1.885080757	-1.460281359	-1.582946426	-11.51719188	2.189979658	-5.749333121
	-1.728873252	-0.368621815	-1.95546254	-8.205365457	18.52014164	-1.881160298
	-2.007605767	1.796791017	-2.221009611	-13.00185666	19.05801396	-2.815636989
	-0.95689311	-1.149246256	-0.395860733	-3.66713714	10.99890131	-9.88834203
	-1.098684881	-1.144744907	-0.966104666	-8.486024819	11.46952827	-7.141610397
	-1.496207543	-1.04737856	-0.14268383	-9.748914475	15.23526008	-13.01097621
	1.425850018	1.184799073	0.609476823	9.069868166	8.404899642	0.846522075
	2.094695217	1.739700176	2.610227531	8.836160356	11.59165196	2.718847895
	1.377496985	1.048736443	1.089223858	21.01340385	12.39928438	5.530042159
	1.885080757	1.460281359	1.582946426	11.51719188	2.189979658	5.749333121
	1.728873252	0.368621815	1.95546254	8.205365457	18.52014164	1.881160298
	2.007605767	1.796791017	2.221009611	13.00185666	19.05801396	2.815636989
	0.95689311	1.149246256	0.395860733	3.66713714	10.99890131	9.88834203
	1.098684881	1.144744907	0.966104666	8.486024819	11.46952827	7.141610397
	1.496207543	1.04737856	0.14268383	9.748914475	15.23526008	13.01097621
**Mean**	1.563487503	1.215588845	1.285888447	10.39399142	12.20751788	5.509163464

LEO_leap_x	LEO_leap_y	LEO_leap_z	LEC_leap_x	LEC_leap_y	LEC_leap_z
0.249517233	-1.616683967	0.284835116	-17.32804007	-0.236213573	-79.60536421
0.628596688	-2.780609237	-0.000197689	10.59054892	-0.706924273	-12.34679603
0.823123202	1.923072786	-1.691736018	14.28370864	-9.908927832	-9.81899455
1.080718025	-2.23246767	-1.072977674	-12.86141277	-21.26388541	-102.4438733
-1.115110969	-2.45286559	0.184348924	7.707031654	-4.864112673	-11.88547003
2.402441634	-1.742355298	-1.63992926	11.47879942	-21.51781461	-17.38874594
-1.194124517	-2.304283037	2.775587873	-0.151381196	-2.76815783	-6.240152412
1.493981331	-0.154571714	0.138646543	-2.388667259	-17.69090263	-26.50860451
-0.124341561	-0.90232837	-1.467119177	3.947550736	-14.77500904	-14.90622572
0.249517233	1.616683967	0.284835116	17.32804007	0.236213573	79.60536421
0.628596688	2.780609237	0.000197689	10.59054892	0.706924273	12.34679603
0.823123202	1.923072786	1.691736018	14.28370864	9.908927832	9.81899455
1.080718025	2.23246767	1.072977674	12.86141277	21.26388541	102.4438733
1.115110969	2.45286559	0.184348924	7.707031654	4.864112673	11.88547003
2.402441634	1.742355298	1.63992926	11.47879942	21.51781461	17.38874594
1.194124517	2.304283037	2.775587873	0.151381196	2.76815783	6.240152412
1.493981331	0.154571714	0.138646543	2.388667259	17.69090263	26.50860451
0.124341561	0.90232837	1.467119177	3.947550736	14.77500904	14.90622572
1.012439462	1.789915297	1.028375364	8.970793406	10.41466087	31.23824741

REO_qtm_x	REO_qtm_y	REO_qtm_z	REC_qtm_x	REC_qtm_y	REC_qtm_z
-1.132895919	-0.39658733	-1.11379683	-9.496127048	-0.518673557	10.05946705
-0.509662833	2.046592838	-2.605793149	-9.674333604	3.541665881	9.927127049
-1.527807809	-0.615769336	0.631029117	-23.05442534	-5.338195949	14.45729658
0.483351566	1.908577972	-3.479582345	-11.73059752	7.098899962	3.566065747
0.073577615	2.255540295	-1.241470347	-9.043261045	1.10434216	21.21905126
0.170241459	1.152077226	-0.713124643	-13.62580598	3.745777755	20.96450494
-0.779705495	0.988120822	-2.956261196	-4.606780288	10.85870734	12.76024566
-1.566007539	0.925782608	-1.1920678	-9.125233302	8.419040618	13.6957958
-1.059075736	-0.160937397	-1.743783167	-9.71235395	15.2718128	16.75568239
1.132895919	0.39658733	1.11379683	9.496127048	0.518673557	10.05946705
0.509662833	2.046592838	2.605793149	9.674333604	3.541665881	9.927127049
1.527807809	0.615769336	0.631029117	23.05442534	5.338195949	14.45729658
0.483351566	1.908577972	3.479582345	11.73059752	7.098899962	3.566065747
0.073577615	2.255540295	1.241470347	9.043261045	1.10434216	21.21905126
0.170241459	1.152077226	0.713124643	13.62580598	3.745777755	20.96450494
0.779705495	0.988120822	2.956261196	4.606780288	10.85870734	12.76024566
1.566007539	0.925782608	1.1920678	9.125233302	8.419040618	13.6957958
1.059075736	0.160937397	1.743783167	9.71235395	15.2718128	16.75568239
0.811369552	1.161109536	1.741878733	11.11876868	6.210790669	13.71169294

LEO_qtm_x	LEO_qtm_y	LEO_qtm_z	LEC_qtm_x	LEC_qtm_y	LEC_qtm_z
1.311900374	0.334921604	0.903958032	10.34110351	5.417934309	-0.467839103
-0.55007329	-0.663822592	1.474728358	15.69921505	12.61433539	1.987345255
0.27910653	0.039361109	2.631367777	17.10818879	13.73080459	-5.569190508
0.482547042	-0.729902661	-0.178471274	20.13111374	15.52712215	-5.957974809
-0.132232534	-0.404895646	2.61178661	13.0441262	16.62442489	-3.030491711
1.94628151	0.257204509	-1.182647054	17.2767223	19.30229242	-17.5572185
1.153781967	-0.370983026	-0.149843797	4.195493459	8.96443505	-0.961959929
-0.223951995	-1.21314846	0.890110562	1.223207409	31.8566105	-16.98182828
-0.40465614	-0.673335857	1.570514916	5.93418667	21.23636277	-10.52391609
1.311900374	0.334921604	0.903958032	10.34110351	5.417934309	0.467839103
0.55007329	0.663822592	1.474728358	15.69921505	12.61433539	1.987345255
0.27910653	0.039361109	2.631367777	17.10818879	13.73080459	5.569190508
0.482547042	0.729902661	0.178471274	20.13111374	15.52712215	5.957974809
0.132232534	0.404895646	2.61178661	13.0441262	16.62442489	3.030491711
1.94628151	0.257204509	1.182647054	17.2767223	19.30229242	17.5572185
1.153781967	0.370983026	0.149843797	4.195493459	8.96443505	0.961959929
0.223951995	1.21314846	0.890110562	1.223207409	31.8566105	16.98182828
0.40465614	0.673335857	1.570514916	5.93418667	21.23636277	10.52391609
0.720503487	0.520841718	1.288158709	11.66148412	16.14159134	7.004196021

Hand/axis	REO_leap_x	REO_leap_y	REO_leap_z	REC_leap_x	REC_leap_y	REC_leap_z
	0.422226955	3.894592334	0.182758297	4.25990192	-9.92328646	-15.06396017
	-0.525408773	2.873352072	0.347067289	4.848398383	-3.00795008	-15.63851087
	2.93913276	2.603378366	0.268962092	11.13525457	-0.695816409	-21.79009832
	2.234010933	7.658134479	0.243481695	13.922888	-4.131592148	-28.21539137
	0.839898945	7.324989304	0.365573504	20.55413163	-7.568619024	-23.51880181
	0.774357267	4.180557187	0.632038153	10.46902301	0.377479541	-18.53078178
	-0.23965386	7.009666481	2.253134023	6.796133902	-1.94084944	-21.86057098
	3.396647963	7.610542287	1.179358015	23.25039926	-2.645958112	-34.01903921
	2.114270953	4.9010925	1.795255187	28.63029862	-4.498108049	-37.83522036
	0.422226955	3.894592334	0.182758297	4.25990192	9.92328646	15.06396017
	0.525408773	2.873352072	0.347067289	4.848398383	3.00795008	15.63851087
	2.93913276	2.603378366	0.268962092	11.13525457	0.695816409	21.79009832
	2.234010933	7.658134479	0.243481695	13.922888	4.131592148	28.21539137
	0.839898945	7.324989304	0.365573504	20.55413163	7.568619024	23.51880181
	0.774357267	4.180557187	0.632038153	10.46902301	0.377479541	18.53078178
	0.23965386	7.009666481	2.253134023	6.796133902	1.94084944	21.86057098
	3.396647963	7.610542287	1.179358015	23.25039926	2.645958112	34.01903921
	2.114270953	4.9010925	1.795255187	28.63029862	4.498108049	37.83522036
**Mean**	1.498400934	5.339589446	0.807514251	13.76293659	3.865517696	24.0524861

LEO_leap_x	LEO_leap_y	LEO_leap_z	LEC_leap_x	LEC_leap_y	LEC_leap_z
-4.098082798	2.265205483	-0.11693092	19.84135688	-0.820411504	11.84896004
-1.823294911	1.566062974	-0.36205464	17.52659297	4.16335328	3.202035534
-3.213469094	6.492492713	-0.551959192	14.7368493	11.55099498	9.314578179
3.418837852	-1.075943041	-0.530999264	20.31893044	13.9775086	2.4303236
-2.847718332	2.98741167	-1.034054079	21.6397939	12.01222995	2.938035847
-0.860157215	3.458229216	-1.018720607	27.31093074	5.872367694	-1.139538038
-1.897402924	0.701751557	-0.915069676	19.72660886	12.21189106	-18.28891457
-3.762456139	2.631309584	-0.669343362	20.29971399	10.87220012	-11.71951141
-2.166842613	1.364555485	-0.416436285	29.83438362	9.067436187	-26.57636914
4.098082798	2.265205483	0.11693092	19.84135688	0.820411504	11.84896004
1.823294911	1.566062974	0.36205464	17.52659297	4.16335328	3.202035534
3.213469094	6.492492713	0.551959192	14.7368493	11.55099498	9.314578179
3.418837852	1.075943041	0.530999264	20.31893044	13.9775086	2.4303236
2.847718332	2.98741167	1.034054079	21.6397939	12.01222995	2.938035847
0.860157215	3.458229216	1.018720607	27.31093074	5.872367694	1.139538038
1.897402924	0.701751557	0.915069676	19.72660886	12.21189106	18.28891457
3.762456139	2.631309584	0.669343362	20.29971399	10.87220012	11.71951141
2.166842613	1.364555485	0.416436285	29.83438362	9.067436187	26.57636914
2.676473542	2.504773525	0.623952003	21.24835119	8.949821487	9.717585151

REO_qtm_x	REO_qtm_y	REO_qtm_z	REC_qtm_x	REC_qtm_y	REC_qtm_z
1.08196049	-0.65254821	1.502660099	4.028228331	16.06169805	-11.25145697
-1.399918124	-1.097040725	1.657903934	1.559976543	18.06332199	-5.516808076
-1.098003288	-1.489029497	1.753848976	5.70520451	25.16509968	-4.255074299
-0.476134328	-0.530223824	2.175336085	10.76219287	30.48765265	-5.759143067
-0.291421455	-1.258415082	1.566758293	15.20989049	25.14957402	-11.10370894
-0.855752384	-1.99683946	2.763886119	4.087604679	20.48231757	-4.542573884
-1.302233749	-3.078673872	3.589795141	2.541971912	26.60476326	-8.735116084
-0.449635183	-1.823680873	3.478088484	13.75581334	37.29460636	-4.444475916
-1.684409495	-2.237906281	1.8441957	19.57475635	43.5742595	-6.90128108
1.08196049	0.65254821	1.502660099	4.028228331	16.06169805	11.25145697
1.399918124	1.097040725	1.657903934	1.559976543	18.06332199	5.516808076
1.098003288	1.489029497	1.753848976	5.70520451	25.16509968	4.255074299
0.476134328	0.530223824	2.175336085	10.76219287	30.48765265	5.759143067
0.291421455	1.258415082	1.566758293	15.20989049	25.14957402	11.10370894
0.855752384	1.99683946	2.763886119	4.087604679	20.48231757	4.542573884
1.302233749	3.078673872	3.589795141	2.541971912	26.60476326	8.735116084
0.449635183	1.823680873	3.478088484	13.75581334	37.29460636	4.444475916
1.684409495	2.237906281	1.8441957	19.57475635	43.5742595	6.90128108
0.959940944	1.573817536	2.259163648	8.580626559	26.98703256	6.945515369

LEO_qtm_x	LEO_qtm_y	LEO_qtm_z	LEC_qtm_x	LEC_qtm_y	LEC_qtm_z
-0.38748398	1.625624326	0.228112238	19.11027645	-17.06357783	-2.757207226
0.105355421	1.488015184	0.348083109	14.46979173	-4.408628537	10.6386357
0.339725529	-0.208079023	2.43813609	14.93179951	-10.62952706	11.21109848
0.984263591	-0.557729951	2.2606688	22.21416461	-6.94078288	11.88491459
0.70171246	0.650959831	2.378530187	25.06796444	-5.755293692	10.55316144
-0.542311876	1.467498413	1.07146484	24.05084047	-1.899104967	7.804786863
0.453265168	0.891866036	1.929195672	24.71677978	18.37345615	7.868236857
0.287834947	1.104768282	0.289056876	22.12619793	10.19595246	8.072367303
0.870080388	0.888982594	1.001408377	33.04061958	27.33053121	5.228701391
0.38748398	1.625624326	0.228112238	19.11027645	17.06357783	2.757207226
0.105355421	1.488015184	0.348083109	14.46979173	4.408628537	10.6386357
0.339725529	0.208079023	2.43813609	14.93179951	10.62952706	11.21109848
0.984263591	0.557729951	2.2606688	22.21416461	6.94078288	11.88491459
0.70171246	0.650959831	2.378530187	25.06796444	5.755293692	10.55316144
0.542311876	1.467498413	1.07146484	24.05084047	1.899104967	7.804786863
0.453265168	0.891866036	1.929195672	24.71677978	18.37345615	7.868236857
0.287834947	1.104768282	0.289056876	22.12619793	10.19595246	8.072367303
0.870080388	0.888982594	1.001408377	33.04061958	27.33053121	5.228701391
0.519114818	0.987058182	1.327184021	22.19204828	11.39965053	8.446567761

Hand/axis	REO_leap_x	REO_leap_y	REO_leap_z	REC_leap_x	REC_leap_y	REC_leap_z
	-0.333676091	8.975853036	2.721425122	-35.69985886	29.27755726	25.23679247
	1.925035907	5.484771859	1.445583053	-35.99414567	38.15711207	32.2335129
	1.239398627	2.905113338	0.149458536	-39.91538328	33.29213695	36.66285508
	0.151149203	2.428756649	-0.913607412	-38.91818732	27.17010488	39.99443429
	0.884440347	2.203277547	-0.097727439	-45.07177817	17.24559773	37.94045062
	2.464506375	4.684295735	1.488629154	-49.03447466	23.00761409	41.12155905
	-0.047173117	2.130783171	-1.381434134	-42.6839193	21.33663178	47.386446
	0.288641156	1.865028405	-0.272488954	-44.84535688	23.97611225	41.78219599
	-1.336845958	4.043785152	1.254277372	-37.23273391	29.9313431	41.26208295
	0.333676091	8.975853036	2.721425122	35.69985886	29.27755726	25.23679247
	1.925035907	5.484771859	1.445583053	35.99414567	38.15711207	32.2335129
	1.239398627	2.905113338	0.149458536	39.91538328	33.29213695	36.66285508
	0.151149203	2.428756649	0.913607412	38.91818732	27.17010488	39.99443429
	0.884440347	2.203277547	0.097727439	45.07177817	17.24559773	37.94045062
	2.464506375	4.684295735	1.488629154	49.03447466	23.00761409	41.12155905
	0.047173117	2.130783171	1.381434134	42.6839193	21.33663178	47.386446
	0.288641156	1.865028405	0.272488954	44.84535688	23.97611225	41.78219599
	1.336845958	4.043785152	1.254277372	37.23273391	29.9313431	41.26208295
**Mean**	0.963429642	3.857962766	1.080514575	41.043982	27.04380112	38.1800366

LEO_leap_x	LEO_leap_y	LEO_leap_z	LEC_leap_x	LEC_leap_y	LEC_leap_z	REO_qtm_x
-3.242305247	3.119651765	-0.582516785	21.75063876	31.1232376	20.11548469	-1.976469376
-1.930603607	2.690132154	0.987004278	23.41530231	44.48813624	17.98073913	-1.824257591
3.366624269	1.848495486	1.8705678	33.82504944	45.7832946	20.21138795	-2.334152489
-2.617337519	3.956955035	0.58081319	26.58023212	44.78009031	3.35435161	-3.355269448
-5.28858511	4.147529739	0.118153024	36.92024872	55.95278912	-8.649647239	-2.524813108
-2.154351184	4.501480098	3.546410739	32.39900482	51.44618983	-2.979132775	-1.925415298
-3.669443903	5.798133949	2.219469584	50.95265608	70.11411288	13.79389545	-2.401840248
-3.900731686	3.518905495	2.488031975	45.23475863	71.50047302	7.231182274	-1.667246627
-5.510392198	-2.47022241	-5.828091496	54.53504423	42.15847766	2.277950841	-2.085885997
3.242305247	3.119651765	0.582516785	21.75063876	31.1232376	20.11548469	1.976469376
1.930603607	2.690132154	0.987004278	23.41530231	44.48813624	17.98073913	1.824257591
3.366624269	1.848495486	1.8705678	33.82504944	45.7832946	20.21138795	2.334152489
2.617337519	3.956955035	0.58081319	26.58023212	44.78009031	3.35435161	3.355269448
5.28858511	4.147529739	0.118153024	36.92024872	55.95278912	8.649647239	2.524813108
2.154351184	4.501480098	3.546410739	32.39900482	51.44618983	2.979132775	1.925415298
3.669443903	5.798133949	2.219469584	50.95265608	70.11411288	13.79389545	2.401840248
3.900731686	3.518905495	2.488031975	45.23475863	71.50047302	7.231182274	1.667246627
5.510392198	2.470222414	5.828091496	54.53504423	42.15847766	2.277950841	2.085885997
3.520041636	3.561278459	2.024562097	36.17921501	50.81631125	10.73264133	2.232816687

REO_qtm_y	REO_qtm_z	REC_qtm_x	REC_qtm_y	REC_qtm_z	LEO_qtm_x
-3.494572939	8.316270769	-39.6163193	-25.29638422	27.26216488	-2.921328311
0.539268891	5.13837515	-41.26647623	-31.90405991	34.48925677	-1.099162436
2.121791739	1.190040191	-44.291962	-35.85298118	30.33282796	2.283156515
1.15581294	-1.262440409	-44.67203901	-41.36188475	18.74983815	-1.42037934
1.72674558	0.82271179	-49.72679334	-37.85613647	16.03299149	-3.663385218
1.857464959	1.366726675	-53.07633294	-41.97867019	24.7623587	-2.337134866
2.896565802	1.334927102	-44.39377467	-45.23421755	13.95046362	-2.699561293
1.317362	0.433295535	-47.45528946	-39.70429488	19.40884232	-3.555907302
-0.545461696	3.944772754	-36.04776624	-37.55656891	23.91632139	-3.104593807
3.494572939	8.316270769	39.6163193	25.29638422	27.26216488	2.921328311
0.539268891	5.13837515	41.26647623	31.90405991	34.48925677	1.099162436
2.121791739	1.190040191	44.291962	35.85298118	30.33282796	2.283156515
1.15581294	1.262440409	44.67203901	41.36188475	18.74983815	1.42037934
1.72674558	0.82271179	49.72679334	37.85613647	16.03299149	3.663385218
1.857464959	1.366726675	53.07633294	41.97867019	24.7623587	2.337134866
2.896565802	1.334927102	44.39377467	45.23421755	13.95046362	2.699561293
1.317362	0.433295535	47.45528946	39.70429488	19.40884232	3.555907302
0.545461696	3.944772754	36.04776624	37.55656891	23.91632139	3.104593807
1.739449616	2.645506708	44.5051948	37.41613312	23.21167392	2.564956565

LEO_qtm_y	LEO_qtm_z	LEC_qtm_x	LEC_qtm_y	LEC_qtm_z
2.330702384	2.035409091	19.60968133	-25.78017878	33.79816019
1.280931117	1.425062793	23.36182983	-23.77475386	43.34924552
-1.955271469	-1.180885727	32.29361329	-23.43316085	44.94302668
0.393596193	2.985547173	25.93347752	-5.472735188	41.63274776
-0.181311334	2.170912884	39.74374171	6.315018273	53.88316086
-3.325455439	3.404740473	35.46164073	-1.394889415	47.0673898
-0.462173799	3.169738592	53.39370212	-18.29832932	61.75120287
-2.201032378	2.171969915	43.83141687	-8.445854168	68.19028646
7.509299138	-3.818866636	53.37933251	-5.937776006	37.12896135
2.330702384	2.035409091	19.60968133	25.78017878	33.79816019
1.280931117	1.425062793	23.36182983	23.77475386	43.34924552
1.955271469	1.180885727	32.29361329	23.43316085	44.94302668
0.393596193	2.985547173	25.93347752	5.472735188	41.63274776
0.181311334	2.170912884	39.74374171	6.315018273	53.88316086
3.325455439	3.404740473	35.46164073	1.394889415	47.0673898
0.462173799	3.169738592	53.39370212	18.29832932	61.75120287
2.201032378	2.171969915	43.83141687	8.445854168	68.19028646
7.509299138	3.818866636	53.37933251	5.937776006	37.12896135
2.182197028	2.484792587	36.33427066	13.2058551	47.97157572

Hand/axis	REO_leap_x	REO_leap_y	REO_leap_z	REC_leap_x	REC_leap_y	REC_leap_z
	-1.036304329	0.435142368	0.241375693	-7.589045388	0.709518646	1.922376105
	3.99044335	0.568809372	-1.533056025	-23.86756249	12.04501343	10.88042548
	4.064062785	2.600059417	0.862292531	-24.63690645	3.965828036	5.326510215
	4.473913055	-1.177025011	-1.89196086	-33.94666229	28.48297086	14.56826737
	1.575076274	2.307441696	1.620266684	-32.10017652	47.93654637	26.90134054
	1.090156236	2.451789681	0.385395527	-16.90869629	35.21904003	4.624240211
	1.118239757	4.863822886	2.198124414	-29.91409183	48.33279425	16.39984421
	3.620046731	1.254333402	-1.493441354	-37.8712321	46.37366488	10.84691997
	0.280939984	-0.161781331	-3.144652122	-29.9052268	51.61303697	19.38407653
	1.036304329	0.435142368	0.241375693	7.589045388	0.709518646	1.922376105
	3.99044335	0.568809372	1.533056025	23.86756249	12.04501343	10.88042548
	4.064062785	2.600059417	0.862292531	24.63690645	3.965828036	5.326510215
	4.473913055	1.177025011	1.89196086	33.94666229	28.48297086	14.56826737
	1.575076274	2.307441696	1.620266684	32.10017652	47.93654637	26.90134054
	1.090156236	2.451789681	0.385395527	16.90869629	35.21904003	4.624240211
	1.118239757	4.863822886	2.198124414	29.91409183	48.33279425	16.39984421
	3.620046731	1.254333402	1.493441354	37.8712321	46.37366488	10.84691997
	0.280939984	0.161781331	3.144652122	29.9052268	51.61303697	19.38407653
**Mean**	2.361020278	1.757800574	1.485618357	26.30440002	30.51982372	12.31711118

LEO_leap_x	LEO_leap_y	LEO_leap_z	LEC_leap_x	LEC_leap_y	LEC_leap_z
-4.772988636	-2.788833521	-0.574451391	14.7893879	10.55468755	15.59392505
-4.789208435	0.377868613	-0.140898781	28.88474682	32.4781723	4.903435591
-4.849121762	-3.410911485	0.644773724	30.59944992	25.37950912	1.609756772
-2.120242501	0.639076236	-0.416104886	35.25726807	12.43482228	11.74715714
-3.262715321	-0.198715154	-0.11246091	38.38170168	24.51989747	13.81559279
-4.69553299	3.170745899	-0.111647846	42.56144055	7.779891975	33.49434807
-2.142276937	1.974693334	-1.444441861	34.24593227	11.12157435	17.95187812
-5.194074868	0.564048804	-1.239666263	44.63928122	17.00448611	27.49955991
-2.378038861	-1.735374557	0.201074298	36.43318604	46.80020167	18.14651446
4.772988636	2.788833521	0.574451391	14.7893879	10.55468755	15.59392505
4.789208435	0.377868613	0.140898781	28.88474682	32.4781723	4.903435591
4.849121762	3.410911485	0.644773724	30.59944992	25.37950912	1.609756772
2.120242501	0.639076236	0.416104886	35.25726807	12.43482228	11.74715714
3.262715321	0.198715154	0.11246091	38.38170168	24.51989747	13.81559279
4.69553299	3.170745899	0.111647846	42.56144055	7.779891975	33.49434807
2.142276937	1.974693334	1.444441861	34.24593227	11.12157435	17.95187812
5.194074868	0.564048804	1.239666263	44.63928122	17.00448611	27.49955991
2.378038861	1.735374557	0.201074298	36.43318604	46.80020167	18.14651446
3.800466701	1.651140845	0.542835551	33.97693272	20.89702698	16.08468532

REO_qtm_x	REO_qtm_y	REO_qtm_z	REC_qtm_x	REC_qtm_y	REC_qtm_z
-0.718104638	-0.705325707	0.265218576	-4.665568704	-4.690781166	-5.504858344
-0.280222581	2.340740411	-1.097960137	-23.30960818	-11.09837579	7.459278018
2.585221358	2.253666184	-2.240784705	-16.90928509	-8.730939069	8.413535885
1.380336586	1.447496926	-1.762202172	-39.95818705	-22.43443213	18.98554043
-2.195302781	-0.816600971	-1.296485694	-32.56105051	-30.58853682	40.96662197
-1.240933556	-0.81244581	0.097701384	-30.54875049	-19.59740873	26.91364457
-2.27033931	-1.936866714	-0.166504824	-38.45468604	-23.84298271	38.88597755
-0.07625928	2.422048013	-2.683701733	-45.51484925	-18.26292434	39.64678691
-2.614205601	4.207640396	-1.878839411	-35.75398362	-21.84617164	39.73550266
0.718104638	0.705325707	0.265218576	4.665568704	4.690781166	5.504858344
0.280222581	2.340740411	1.097960137	23.30960818	11.09837579	7.459278018
2.585221358	2.253666184	2.240784705	16.90928509	8.730939069	8.413535885
1.380336586	1.447496926	1.762202172	39.95818705	22.43443213	18.98554043
2.195302781	0.816600971	1.296485694	32.56105051	30.58853682	40.96662197
1.240933556	0.81244581	0.097701384	30.54875049	19.59740873	26.91364457
2.27033931	1.936866714	0.166504824	38.45468604	23.84298271	38.88597755
0.07625928	2.422048013	2.683701733	45.51484925	18.26292434	39.64678691
2.614205601	4.207640396	1.878839411	35.75398362	21.84617164	39.73550266
1.484547299	1.882536793	1.276599848	29.74177433	17.89917249	25.16797181

LEO_qtm_x	LEO_qtm_y	LEO_qtm_z	LEC_qtm_x	LEC_qtm_y	LEC_qtm_z
-1.715230889	-0.071749457	-0.896257475	17.70242183	-16.98210648	11.39606469
-2.777028637	-0.230715745	-3.010151848	28.32414828	-4.23288965	26.95906761
-1.577558	-0.186547521	-4.098831516	29.45940461	-2.353499569	23.30665024
-0.237755028	1.70169627	-1.525866243	39.56042484	-14.42840641	11.45383115
-0.477620914	1.525075009	-2.156781677	44.7103943	-15.47333494	10.81147871
-1.325764197	0.776684022	0.385506356	48.2822279	-35.4065653	5.582618957
-0.625018834	3.058408471	-1.419334656	36.78284441	-17.72076166	10.27169857
0.134536311	2.304142087	-3.011333259	54.26049974	-30.48021392	19.69266328
-0.700340631	1.581268194	-1.531060116	40.90674329	-21.79495522	43.33294914
1.715230889	0.071749457	0.896257475	17.70242183	16.98210648	11.39606469
2.777028637	0.230715745	3.010151848	28.32414828	4.23288965	26.95906761
1.577558	0.186547521	4.098831516	29.45940461	2.353499569	23.30665024
0.237755028	1.70169627	1.525866243	39.56042484	14.42840641	11.45383115
0.477620914	1.525075009	2.156781677	44.7103943	15.47333494	10.81147871
1.325764197	0.776684022	0.385506356	48.2822279	35.4065653	5.582618957
0.625018834	3.058408471	1.419334656	36.78284441	17.72076166	10.27169857
0.134536311	2.304142087	3.011333259	54.26049974	30.48021392	19.69266328
0.700340631	1.581268194	1.531060116	40.90674329	21.79495522	43.33294914
1.06342816	1.270698531	2.003902572	37.77656769	17.6525259	18.08966915

**Hand/axis**	REO_leap_x	REO_leap_y	REO_leap_z	REC_leap_x	REC_leap_y	REC_leap_z
	-1.423964358	8.143852135	-0.60575815	-22.36202237	14.12243644	-5.985272965
	-1.273904543	3.589591735	2.867258107	-12.56400509	9.044943996	-8.496778417
	1.950406717	5.369826809	2.200280766	-28.61113168	1.05045271	-1.60791371
	-2.979414607	1.00283051	-0.504980235	-18.65707213	3.928199215	-12.22805584
	-2.628884273	3.351455353	0.666451974	-14.43283102	2.775367002	-13.03810869
	-2.384295044	4.04279294	1.12046193	-15.54974212	7.437003507	-11.98457971
	-0.541659077	5.657058184	-0.668948844	4.783655184	2.125701564	-12.63336949
	0.04505686	4.569015069	-1.321665181	16.02873592	14.47988123	-18.76016508
	-3.244422863	3.63122505	-1.986301356	-5.232656675	19.50714813	-9.541107771
	1.423964358	8.143852135	0.60575815	22.36202237	14.12243644	5.985272965
	1.273904543	3.589591735	2.867258107	12.56400509	9.044943996	8.496778417
	1.950406717	5.369826809	2.200280766	28.61113168	1.05045271	1.60791371
	2.979414607	1.00283051	0.504980235	18.65707213	3.928199215	12.22805584
	2.628884273	3.351455353	0.666451974	14.43283102	2.775367002	13.03810869
	2.384295044	4.04279294	1.12046193	15.54974212	7.437003507	11.98457971
	0.541659077	5.657058184	0.668948844	4.783655184	2.125701564	12.63336949
	0.04505686	4.569015069	1.321665181	16.02873592	14.47988123	18.76016508
	3.244422863	3.63122505	1.986301356	5.232656675	19.50714813	9.541107771
**Mean**	1.830223149	4.373071976	1.326900727	15.35798358	8.274570422	10.47503907

LEO_leap_x	LEO_leap_y	LEO_leap_z	LEC_leap_x	LEC_leap_y	LEC_leap_z
-5.692312142	4.280464078	-0.541510117	4.776366497	4.874946818	4.693755035
-1.592119781	-2.641234986	1.182243694	20.39611124	6.620788226	-4.832553635
-2.586802625	4.200706548	4.985823247	4.205500738	9.626525962	-1.096077195
-1.516280822	4.601028473	3.122844722	6.700333599	16.41606889	0.603131334
-4.382202883	4.266601637	4.025575454	7.732876811	17.29676108	1.347122655
-2.308965882	3.058517343	4.180217313	3.340758026	2.135940318	2.410544264
-0.143247194	3.114143079	3.609957694	8.730549112	14.11843866	-9.547584655
3.004045913	1.998535015	3.106265599	9.007083374	13.43617222	-9.044240541
0.917820312	6.810149959	6.4227393	4.864773478	24.57617224	-12.01720315
5.692312142	4.280464078	0.541510117	4.776366497	4.874946818	4.693755035
1.592119781	2.641234986	1.182243694	20.39611124	6.620788226	4.832553635
2.586802625	4.200706548	4.985823247	4.205500738	9.626525962	1.096077195
1.516280822	4.601028473	3.122844722	6.700333599	16.41606889	0.603131334
4.382202883	4.266601637	4.025575454	7.732876811	17.29676108	1.347122655
2.308965882	3.058517343	4.180217313	3.340758026	2.135940318	2.410544264
0.143247194	3.114143079	3.609957694	8.730549112	14.11843866	9.547584655
3.004045913	1.998535015	3.106265599	9.007083374	13.43617222	9.044240541
0.917820312	6.810149959	6.4227393	4.864773478	24.57617224	12.01720315
2.46042195	3.885709013	3.464130794	7.750483653	12.12242382	5.065801385

REO_qtm_x	REO_qtm_y	REO_qtm_z	REC_qtm_x	REC_qtm_y	REC_qtm_z
0.448198056	0.744793369	-0.900773583	-21.72139647	6.609880783	13.04918312
1.542952504	-2.263710697	4.466490011	-21.96325018	6.622447979	2.808985176
1.429902547	0.207124984	1.23206803	-28.38262096	3.125044362	-0.200682024
0.587185213	-0.702387996	-0.268536666	-17.78275577	12.89526686	3.327325955
1.472174947	0.166004189	-0.789493615	-11.91972864	16.19040329	0.247255471
1.818146017	-1.388647787	0.885695266	-14.34779151	14.19473127	5.691099773
1.988680786	-1.283348993	1.027489259	-18.40512065	7.20437197	16.74195853
1.011336263	0.216266878	-1.00621275	-10.02960001	9.606826081	22.13677257
-0.932578664	1.758200041	-0.58226293	-20.83048905	11.18540887	14.68341339
0.448198056	0.744793369	0.900773583	21.72139647	6.609880783	13.04918312
1.542952504	2.263710697	4.466490011	21.96325018	6.622447979	2.808985176
1.429902547	0.207124984	1.23206803	28.38262096	3.125044362	0.200682024
0.587185213	0.702387996	0.268536666	17.78275577	12.89526686	3.327325955
1.472174947	0.166004189	0.789493615	11.91972864	16.19040329	0.247255471
1.818146017	1.388647787	0.885695266	14.34779151	14.19473127	5.691099773
1.988680786	1.283348993	1.027489259	18.40512065	7.20437197	16.74195853
1.011336263	0.216266878	1.00621275	10.02960001	9.606826081	22.13677257
0.932578664	1.758200041	0.58226293	20.83048905	11.18540887	14.68341339
1.247906111	0.970053882	1.239891345	18.37586147	9.737153496	8.765186223

LEO_qtm_x	LEO_qtm_y	LEO_qtm_z	LEC_qtm_x	LEC_qtm_y	LEC_qtm_z
-2.813199232	-0.3168556	3.614517382	1.366457748	-4.056633095	6.769472309
-1.347378264	1.915869951	-1.645855461	11.50411118	6.133528317	8.601014284
-0.813471549	-2.966881889	3.096556582	-1.393108482	0.725164959	8.401393069
-0.590519806	-1.734751782	1.311734516	1.750204507	-2.219250207	15.9797502
-1.524360167	-1.641021007	1.270219442	3.286763666	-2.629804016	17.08205091
-0.804974898	-3.002706665	2.468774312	-4.017650524	0.297550567	-0.072703202
0.217033873	-1.874881969	2.031868458	5.948425396	6.361207252	17.03155401
-0.511870643	-3.617949415	3.447515954	4.998840349	9.212886215	14.19717258
-4.252875163	-1.753229075	0.748939236	2.637688958	14.51856396	24.96262496
2.813199232	0.3168556	3.614517382	1.366457748	4.056633095	6.769472309
1.347378264	1.915869951	1.645855461	11.50411118	6.133528317	8.601014284
0.813471549	2.966881889	3.096556582	1.393108482	0.725164959	8.401393069
0.590519806	1.734751782	1.311734516	1.750204507	2.219250207	15.9797502
1.524360167	1.641021007	1.270219442	3.286763666	2.629804016	17.08205091
0.804974898	3.002706665	2.468774312	4.017650524	0.297550567	0.072703202
0.217033873	1.874881969	2.031868458	5.948425396	6.361207252	17.03155401
0.511870643	3.617949415	3.447515954	4.998840349	9.212886215	14.19717258
4.252875163	1.753229075	0.748939236	2.637688958	14.51856396	24.96262496
1.430631511	2.091571928	2.181775705	4.100361201	5.12828762	12.56641506

**Hand/axis**	REO_leap_x	REO_leap_y	REO_leap_z	REC_leap_x	REC_leap_y	REC_leap_z
	-0.568962719	2.150474609	-2.537809584	-13.74392739	3.904953059	-2.059150462
	-0.733959786	2.233497753	-1.472606646	-19.27179417	12.114067	-3.814158692
	-1.103685181	1.56292736	-2.154160727	-8.937792293	15.58915716	-5.253071674
	0.614219633	-0.159919793	-3.511493795	-13.395819	24.86601277	-7.653374577
	0.300597963	0.634079002	-3.298838105	-17.6513411	22.90074921	-10.29139175
	0.075905862	-2.166313142	-3.041029536	-9.86396099	20.27941883	-17.00394578
	1.289083164	-0.234458966	-3.098590394	0.854736493	18.3486252	-20.0440259
	0.747159173	-0.600242591	-3.070484569	-4.538974401	20.94575495	-25.17318009
	1.231522315	-0.356758196	-2.605216617	0.377109068	13.7804871	-23.52318513
	0.568962719	2.150474609	2.537809584	13.74392739	3.904953059	2.059150462
	0.733959786	2.233497753	1.472606646	19.27179417	12.114067	3.814158692
	1.103685181	1.56292736	2.154160727	8.937792293	15.58915716	5.253071674
	0.614219633	0.159919793	3.511493795	13.395819	24.86601277	7.653374577
	0.300597963	0.634079002	3.298838105	17.6513411	22.90074921	10.29139175
	0.075905862	2.166313142	3.041029536	9.86396099	20.27941883	17.00394578
	1.289083164	0.234458966	3.098590394	0.854736493	18.3486252	20.0440259
	0.747159173	0.600242591	3.070484569	4.538974401	20.94575495	25.17318009
	1.231522315	0.356758196	2.605216617	0.377109068	13.7804871	23.52318513
**Mean**	0.740566199	1.122074601	2.754469997	9.848383878	16.96991392	12.75727601

LEO_leap_x	LEO_leap_y	LEO_leap_z	LEC_leap_x	LEC_leap_y	LEC_leap_z
-0.345982975	0.180412218	-2.123477156	2.421502938	26.88170625	0.341060068
0.279146574	-0.916664011	-3.593825431	5.532701234	44.46868902	-3.281293552
0.136578904	-1.77632907	-2.518472139	4.692422106	62.15339664	-0.808197437
0.667321035	-1.339820962	-4.729052453	-0.96075008	48.27613072	-4.332362254
-0.476565488	-0.100242725	-1.741676943	-3.167994875	48.65888973	5.667530624
0.183315709	1.71542364	-2.401574139	-3.109262123	43.6171341	1.212843681
0.451972289	0.851181025	-2.899925228	-5.975235856	55.9096909	3.838249367
-0.664668711	-1.814651521	-3.022831347	-0.168120728	47.47328959	-6.500620003
0.003507999	0.49742901	-3.73165643	-5.16546575	57.33232879	-0.971522995
0.345982975	0.180412218	2.123477156	2.421502938	26.88170625	0.341060068
0.279146574	0.916664011	3.593825431	5.532701234	44.46868902	3.281293552
0.136578904	1.77632907	2.518472139	4.692422106	62.15339664	0.808197437
0.667321035	1.339820962	4.729052453	0.96075008	48.27613072	4.332362254
0.476565488	0.100242725	1.741676943	3.167994875	48.65888973	5.667530624
0.183315709	1.71542364	2.401574139	3.109262123	43.6171341	1.212843681
0.451972289	0.851181025	2.899925228	5.975235856	55.9096909	3.838249367
0.664668711	1.814651521	3.022831347	0.168120728	47.47328959	6.500620003
0.003507999	0.49742901	3.73165643	5.16546575	57.33232879	0.971522995
0.356562187	1.021350465	2.973610141	3.465939521	48.30791731	2.994853331

REO_qtm_x	REO_qtm_y	REO_qtm_z	REC_qtm_x	REC_qtm_y	REC_qtm_z
-0.212907031	-0.449218096	0.344174616	-1.305282267	0.286879885	5.584650424
-0.882905593	0.079313375	-0.018062112	5.776307385	3.966900226	7.645993297
-0.221079641	1.00585189	-0.883256479	1.132420667	5.666711795	16.7758315
0.615646293	1.10955154	-1.606267643	-3.177490833	7.96386862	13.59194602
-0.058591009	1.198451434	-1.873023724	10.78851468	14.32081734	10.5341072
1.497746748	0.591286794	-1.127997077	16.73884664	19.38411761	9.462128057
-0.601371678	0.294822371	-1.165062927	12.06105059	24.95930677	10.06967379
-0.537810049	0.636734538	-0.363865542	17.44454042	24.30757959	4.659125863
-0.451264498	0.690846417	-0.959519552	19.86326801	27.50756313	15.5530695
0.212907031	0.449218096	0.344174616	1.305282267	0.286879885	5.584650424
0.882905593	0.079313375	0.018062112	5.776307385	3.966900226	7.645993297
0.221079641	1.00585189	0.883256479	1.132420667	5.666711795	16.7758315
0.615646293	1.10955154	1.606267643	3.177490833	7.96386862	13.59194602
0.058591009	1.198451434	1.873023724	10.78851468	14.32081734	10.5341072
1.497746748	0.591286794	1.127997077	16.73884664	19.38411761	9.462128057
0.601371678	0.294822371	1.165062927	12.06105059	24.95930677	10.06967379
0.537810049	0.636734538	0.363865542	17.44454042	24.30757959	4.659125863
0.451264498	0.690846417	0.959519552	19.86326801	27.50756313	15.5530695
0.564369171	0.672897384	0.926803297	9.809746832	14.26263833	10.43072507

LEO_qtm_x	LEO_qtm_y	LEO_qtm_z	LEC_qtm_x	LEC_qtm_y	LEC_qtm_z
-0.903209205	1.98224532	-0.044430758	2.92561688	-1.747815242	25.3648683
-1.302962923	2.256920742	-1.728247259	1.826052683	0.287361325	40.80380814
0.111283378	2.302413436	-0.902498304	5.786839765	-2.040167261	58.98338862
-0.156422587	3.482815526	-0.887717851	-1.069680708	1.195090444	45.77051572
-2.080531553	0.906766041	-0.137441059	-4.153689593	-7.098356436	45.68492358
-2.393304783	1.182051758	0.127336274	-5.568192373	-3.610791673	39.1759196
-2.301036601	2.684521314	-1.713176838	-9.201925662	-8.003103682	50.60816057
-1.490840464	3.235295233	-2.003738264	-1.809689431	5.044012791	41.77085319
-2.455811555	3.114015851	-2.242315696	-6.523866937	-1.471952136	54.02407802
0.903209205	1.98224532	0.044430758	2.92561688	1.747815242	25.3648683
1.302962923	2.256920742	1.728247259	1.826052683	0.287361325	40.80380814
0.111283378	2.302413436	0.902498304	5.786839765	2.040167261	58.98338862
0.156422587	3.482815526	0.887717851	1.069680708	1.195090444	45.77051572
2.080531553	0.906766041	0.137441059	4.153689593	7.098356436	45.68492358
2.393304783	1.182051758	0.127336274	5.568192373	3.610791673	39.1759196
2.301036601	2.684521314	1.713176838	9.201925662	8.003103682	50.60816057
1.490840464	3.235295233	2.003738264	1.809689431	5.044012791	41.77085319
2.455811555	3.114015851	2.242315696	6.523866937	1.471952136	54.02407802
1.466155894	2.349671691	1.087433589	4.318394893	3.388738999	44.68739064

Hand/axis	REO_leap_x	REO_leap_y	REO_leap_z	REC_leap_x	REC_leap_y	REC_leap_z
	-1.758574493	-1.497108463	0.323374508	-6.703974114	29.18433369	-53.60573963
	0.216405611	-1.700798141	0.03822293	-21.40877542	18.80015561	-51.94166565
	-2.432430269	0.943962109	-1.036524779	-35.66915562	21.41487113	7.970086962
	-1.770646115	0.582695017	-0.291163376	-34.2235817	8.993957583	16.04844856
	-1.4827612	1.559524519	-1.77970705	-38.82122292	16.33838652	14.5490184
	2.459465565	2.092498712	0.995069897	-38.72864742	7.197395532	17.27486416
	0.189262925	-0.957893367	-0.528680283	-43.07001371	11.82610303	17.10161494
	-3.463410854	2.233894419	-1.650959551	-49.91142156	-0.767601004	14.25358826
	1.977031064	-0.36267088	-1.829718626	-40.66126846	8.992538285	11.1097913
	1.758574493	1.497108463	0.323374508	6.703974114	29.18433369	53.60573963
	0.216405611	1.700798141	0.03822293	21.40877542	18.80015561	51.94166565
	2.432430269	0.943962109	1.036524779	35.66915562	21.41487113	7.970086962
	1.770646115	0.582695017	0.291163376	34.2235817	8.993957583	16.04844856
	1.4827612	1.559524519	1.77970705	38.82122292	16.33838652	14.5490184
	2.459465565	2.092498712	0.995069897	38.72864742	7.197395532	17.27486416
	0.189262925	0.957893367	0.528680283	43.07001371	11.82610303	17.10161494
	3.463410854	2.233894419	1.650959551	49.91142156	0.767601004	14.25358826
	1.977031064	0.36267088	1.829718626	40.66126846	8.992538285	11.1097913
**Mean**	1.749998677	1.325671736	0.941491222	34.3553401	13.72392693	22.65053532

LEO_leap_x	LEO_leap_y	LEO_leap_z	LEC_leap_x	LEC_leap_y	LEC_leap_z
0.059465803	2.392585957	-2.711560245	-3.644394633	18.30505368	2.32647074
0.349626759	1.182685974	-2.379080794	-3.108487611	2.486648628	2.166829769
-2.42625606	2.854217903	-0.551816719	1.024756697	16.36437184	-0.822723925
-2.292549362	0.167053533	-1.226385343	-6.642894251	10.59667226	-3.036578278
-1.052209851	-2.891426032	-3.607964508	-8.116233428	9.984642183	-7.867095149
-3.051082439	2.406128148	1.552137776	-2.505785914	6.038909743	-6.495160095
0.220380641	1.240646461	-0.177649797	0.276690712	2.094215558	-7.120421086
-4.466396912	4.000480758	-0.46026997	-3.570361875	12.11077143	-13.75343837
-4.727950983	4.175270162	-0.122379825	-2.20619845	7.693061695	-15.56655204
0.059465803	2.392585957	2.711560245	3.644394633	18.30505368	2.32647074
0.349626759	1.182685974	2.379080794	3.108487611	2.486648628	2.166829769
2.42625606	2.854217903	0.551816719	1.024756697	16.36437184	0.822723925
2.292549362	0.167053533	1.226385343	6.642894251	10.59667226	3.036578278
1.052209851	2.891426032	3.607964508	8.116233428	9.984642183	7.867095149
3.051082439	2.406128148	1.552137776	2.505785914	6.038909743	6.495160095
0.220380641	1.240646461	0.177649797	0.276690712	2.094215558	7.120421086
4.466396912	4.000480758	0.46026997	3.570361875	12.11077143	13.75343837
4.727950983	4.175270162	0.122379825	2.20619845	7.693061695	15.56655204
2.071768757	2.36783277	1.42102722	3.455089286	9.519371891	6.572807717

REO_qtm_x	REO_qtm_y	REO_qtm_z	REC_qtm_x	REC_qtm_y	REC_qtm_z
-2.080834526	-0.384765006	-1.463240665	-24.92405144	-5.942290685	7.51942376
-0.891976259	-0.629938293	-0.915169205	-24.98529307	-10.05091005	5.338731481
-1.471443899	0.512041639	-0.557693307	-35.15008086	-9.700798343	12.6695079
-1.209313789	-0.148160049	-0.469852764	-35.33853238	-16.31988407	7.344494022
-1.426729589	1.431804593	-0.988493999	-44.47094005	-18.1377683	11.20092149
-1.583017488	-0.82756894	-0.365050413	-45.87884289	-19.95611314	2.593015359
-0.592563678	0.024942897	-0.558036824	-49.04411781	-20.64523299	8.893903968
-1.400179652	0.633681672	-0.612751445	-60.02127288	-15.40159933	-8.051941617
-1.257718808	1.569630753	-1.962284685	-52.57723641	-15.54323531	0.053054042
2.080834526	0.384765006	1.463240665	24.92405144	5.942290685	7.51942376
0.891976259	0.629938293	0.915169205	24.98529307	10.05091005	5.338731481
1.471443899	0.512041639	0.557693307	35.15008086	9.700798343	12.6695079
1.209313789	0.148160049	0.469852764	35.33853238	16.31988407	7.344494022
1.426729589	1.431804593	0.988493999	44.47094005	18.1377683	11.20092149
1.583017488	0.82756894	0.365050413	45.87884289	19.95611314	2.593015359
0.592563678	0.024942897	0.558036824	49.04411781	20.64523299	8.893903968
1.400179652	0.633681672	0.612751445	60.02127288	15.40159933	8.051941617
1.257718808	1.569630753	1.962284685	52.57723641	15.54323531	0.053054042
1.323753076	0.684725982	0.87695259	41.37670753	14.63309247	7.073888182

LEO_qtm_x	LEO_qtm_y	LEO_qtm_z	LEC_qtm_x	LEC_qtm_y	LEC_qtm_z
1.392754501	3.697102193	-1.35701391	-2.638870716	-5.399332301	21.62019643
2.030228825	4.129462809	-1.972725669	0.659106949	-4.670131749	7.84424799
0.137031862	1.513656191	0.047653546	4.104024037	-2.693102277	18.95930568
1.875404813	3.179735104	-1.311593303	-4.266820096	0.939197891	11.94745776
1.540256891	4.133232979	-2.399797171	-2.625990189	6.75590634	9.203526203
-0.069601474	2.422127198	1.275222175	1.947796357	5.443686507	6.819960897
0.942576322	1.213769331	0.349756576	1.401488189	4.407156283	8.268751811
1.4415082	2.302295683	1.000019857	-0.123551662	11.20372755	15.74313125
0.781687539	1.528421415	0.24645412	0.744118068	13.47218649	11.01856569
1.392754501	3.697102193	1.35701391	2.638870716	5.399332301	21.62019643
2.030228825	4.129462809	1.972725669	0.659106949	4.670131749	7.84424799
0.137031862	1.513656191	0.047653546	4.104024037	2.693102277	18.95930568
1.875404813	3.179735104	1.311593303	4.266820096	0.939197891	11.94745776
1.540256891	4.133232979	2.399797171	2.625990189	6.75590634	9.203526203
0.069601474	2.422127198	1.275222175	1.947796357	5.443686507	6.819960897
0.942576322	1.213769331	0.349756576	1.401488189	4.407156283	8.268751811
1.4415082	2.302295683	1.000019857	0.123551662	11.20372755	15.74313125
0.781687539	1.528421415	0.24645412	0.744118068	13.47218649	11.01856569
1.134561158	2.6799781	1.106692925	2.056862918	6.10938082	12.38057152

Hand/axis	REO_leap_x	REO_leap_y	REO_leap_z	REC_leap_x	REC_leap_y	REC_leap_z
	-1.159694546	-1.439300581	0.374472819	-15.41317559	10.1620256	-10.03615998
	3.237185313	3.119842382	0.647207666	-25.59500333	13.5104065	-51.04762696
	0.666547338	3.533127002	-1.075737461	-16.89564713	23.67854279	-9.950338329
	0.283247138	0.924758702	-0.865035376	-27.32529452	31.10306549	-18.39087438
	-3.006439599	-1.425018271	-0.736136053	-30.16598171	30.21872692	-18.52255203
	0.475307101	-1.350753782	1.341344312	-32.28298766	38.71565245	-20.7578464
	-2.572265488	3.273376456	-2.019199737	-18.8398211	42.57450107	-25.93598602
	2.970966066	-3.912208479	2.449639936	-19.69210429	43.17919912	-21.58641003
	0.958101474	0.798797738	-0.879841467	-5.570784391	34.51859305	-27.78368999
	1.159694546	1.439300581	0.374472819	15.41317559	10.1620256	10.03615998
	3.237185313	3.119842382	0.647207666	25.59500333	13.5104065	51.04762696
	0.666547338	3.533127002	1.075737461	16.89564713	23.67854279	9.950338329
	0.283247138	0.924758702	0.865035376	27.32529452	31.10306549	18.39087438
	3.006439599	1.425018271	0.736136053	30.16598171	30.21872692	18.52255203
	0.475307101	1.350753782	1.341344312	32.28298766	38.71565245	20.7578464
	2.572265488	3.273376456	2.019199737	18.8398211	42.57450107	25.93598602
	2.970966066	3.912208479	2.449639936	19.69210429	43.17919912	21.58641003
	0.958101474	0.798797738	0.879841467	5.570784391	34.51859305	27.78368999
**Mean**	1.703306007	2.197464822	1.154290536	21.30897775	29.74007922	22.66794268

LEO_leap_x	LEO_leap_y	LEO_leap_z	LEC_leap_x	LEC_leap_y	LEC_leap_z
-1.829501095	2.803192342	3.248788411	12.10417734	15.56143187	-9.220622582
1.317562898	2.230865414	0.051952609	-4.505364032	52.44806666	-19.03446926
0.510432694	-2.674087744	1.005011341	-136.2958484	-61.91582868	-61.58751175
-3.721338393	2.644424606	-1.56274454	-15.64828386	39.75695046	-23.21525049
3.195933176	1.729095455	0.941649544	7.03398521	27.55004886	-22.09629971
-2.664161707	0.645515384	-1.89239336	2.739292118	36.01094832	-19.30023343
0.156589214	-2.140586808	1.465057563	10.02440561	20.15718095	-16.88619094
3.093668407	8.726318278	0.252579072	8.024191049	43.07891864	-17.2088533
-2.605717665	-7.870468023	1.259945505	10.37702316	36.39756785	-12.36191277
1.829501095	2.803192342	3.248788411	12.10417734	15.56143187	9.220622582
1.317562898	2.230865414	0.051952609	4.505364032	52.44806666	19.03446926
0.510432694	2.674087744	1.005011341	136.2958484	61.91582868	61.58751175
3.721338393	2.644424606	1.56274454	15.64828386	39.75695046	23.21525049
3.195933176	1.729095455	0.941649544	7.03398521	27.55004886	22.09629971
2.664161707	0.645515384	1.89239336	2.739292118	36.01094832	19.30023343
0.156589214	2.140586808	1.465057563	10.02440561	20.15718095	16.88619094
3.093668407	8.726318278	0.252579072	8.024191049	43.07891864	17.2088533
2.605717665	7.870468023	1.259945505	10.37702316	36.39756785	12.36191277
2.121656139	3.496061562	1.297791327	22.97250787	36.98632692	22.32348269

REO_qtm_x	REO_qtm_y	REO_qtm_z	REC_qtm_x	REC_qtm_y	REC_qtm_z
-1.985288125	-0.650374228	0.741628438	-15.24826438	10.37299018	10.98359726
-1.267444251	-0.489746542	2.998991866	-27.31640451	8.948906737	16.84271685
0.742978997	-0.389816001	3.909815831	-18.45448812	13.75489174	19.55367413
0.613634515	0.7586658	4.281304281	-22.89467462	20.50457521	30.94646132
-0.461997563	2.023013578	4.634749443	-24.9069476	21.48937261	28.82350471
9.765806337	10.01280279	6.405797727	-24.48757807	20.13121649	40.98446546
-1.646944053	0.799191801	4.369728281	-17.20620947	25.07397613	43.03992304
-1.039173723	-1.152117207	1.859493121	-21.45967281	22.47738352	47.63144367
-0.975263794	-0.429670524	3.615238807	-2.108715871	31.12595847	36.08868829
1.985288125	0.650374228	0.741628438	15.24826438	10.37299018	10.98359726
1.267444251	0.489746542	2.998991866	27.31640451	8.948906737	16.84271685
0.742978997	0.389816001	3.909815831	18.45448812	13.75489174	19.55367413
0.613634515	0.7586658	4.281304281	22.89467462	20.50457521	30.94646132
0.461997563	2.023013578	4.634749443	24.9069476	21.48937261	28.82350471
9.765806337	10.01280279	6.405797727	24.48757807	20.13121649	40.98446546
1.646944053	0.799191801	4.369728281	17.20620947	25.07397613	43.03992304
1.039173723	1.152117207	1.859493121	21.45967281	22.47738352	47.63144367
0.975263794	0.429670524	3.615238807	2.108715871	31.12595847	36.08868829
2.055392373	1.856155385	3.646305311	19.34255061	19.31991901	30.54383053

LEO_qtm_x	LEO_qtm_y	LEO_qtm_z	LEC_qtm_x	LEC_qtm_y	LEC_qtm_z
0.313831164	-0.126150136	0.114813215	13.46349672	10.08703141	13.8884988
3.331270675	-3.2217238	1.857495445	7.331090385	14.75015611	40.78080206
0.238618652	0.74727179	0.339032722	0.103894847	21.58614198	29.285737
0.432762307	-3.56827755	2.39385394	17.80318108	22.62122359	16.55502311
3.939005494	-3.779461095	0.518895397	16.42723105	20.49271895	23.06845029
1.975066458	-3.301350305	2.267713793	14.85821706	18.9524225	13.71016967
3.125551679	-3.741360262	1.268993596	9.358828535	14.72870117	37.9202439
2.899338168	-3.03826845	1.029084991	18.7213467	10.00590504	34.14635875
3.253848276	-3.234017059	1.35126209	6.912173211	30.83550547	28.80657264
0.313831164	0.126150136	0.114813215	13.46349672	10.08703141	13.8884988
3.331270675	3.2217238	1.857495445	7.331090385	14.75015611	40.78080206
0.238618652	0.74727179	0.339032722	0.103894847	21.58614198	29.285737
0.432762307	3.56827755	2.39385394	17.80318108	22.62122359	16.55502311
3.939005494	3.779461095	0.518895397	16.42723105	20.49271895	23.06845029
1.975066458	3.301350305	2.267713793	14.85821706	18.9524225	13.71016967
3.125551679	3.741360262	1.268993596	9.358828535	14.72870117	37.9202439
2.899338168	3.03826845	1.029084991	18.7213467	10.00590504	34.14635875
3.253848276	3.234017059	1.35126209	6.912173211	30.83550547	28.80657264
2.167699208	2.750875605	1.237905021	11.6643844	18.22886736	26.46242847

**Hand/axis**	REO_leap_x	REO_leap_y	REO_leap_z	REC_leap_x	REC_leap_y	REC_leap_z
	1.498709611	1.747177276	-0.025817985	-14.20339399	10.54798891	2.79653609
	1.785531956	2.250839336	1.381781119	-16.25296742	0.353180391	-10.77785731
	1.429994699	-2.348701612	-0.955418081	2.740896188	17.58427435	-50.34386982
	1.531192806	3.02062987	1.758850016	-0.065356264	8.122329894	-19.32848914
	2.139331962	0.76743325	1.249210619	-4.931700257	8.408462793	-27.93414373
	2.283773867	3.418815671	1.972657858	-4.361772895	15.94469496	-16.89894367
	1.83068954	2.961479052	1.15775171	-17.62207375	14.20748899	-27.88164705
	0.913613506	3.588905468	-0.695718564	-13.47549452	14.59885429	-32.0502376
	0.901913845	1.412689195	-0.354562256	-17.7286182	6.068611329	-31.68510217
	1.498709611	1.747177276	0.025817985	14.20339399	10.54798891	2.79653609
	1.785531956	2.250839336	1.381781119	16.25296742	0.353180391	10.77785731
	1.429994699	2.348701612	0.955418081	2.740896188	17.58427435	50.34386982
	1.531192806	3.02062987	1.758850016	0.065356264	8.122329894	19.32848914
	2.139331962	0.76743325	1.249210619	4.931700257	8.408462793	27.93414373
	2.283773867	3.418815671	1.972657858	4.361772895	15.94469496	16.89894367
	1.83068954	2.961479052	1.15775171	17.62207375	14.20748899	27.88164705
	0.913613506	3.588905468	0.695718564	13.47549452	14.59885429	32.0502376
	0.901913845	1.412689195	0.354562256	17.7286182	6.068611329	31.68510217
**Mean**	1.590527977	2.390741192	1.061307579	10.15358594	10.64843177	24.41075851

LEO_leap_x	LEO_leap_y	LEO_leap_z	LEC_leap_x	LEC_leap_y	LEC_leap_z
-3.065877112	4.029029846	-0.266448408	10.6031313	11.28358443	-3.029200453
-1.604137939	-2.08889774	-2.815950382	18.42666426	14.47624218	-18.28453444
-2.075337629	-1.855537502	-1.935023539	-22.62709557	12.4001539	-95.27150902
-1.71769517	2.028724722	-3.133349957	15.60577614	-0.364914029	-12.09564294
-2.952071815	-0.697303775	-3.205159458	19.01681907	15.26486194	-14.77135987
0.020112207	5.141723625	-0.445980299	18.30739339	9.507820035	-7.696218437
-2.126938129	3.859733593	-2.017021229	20.43378026	21.42589551	-28.20883267
-2.026066269	2.604324245	-1.69370659	20.46833875	46.00681309	-22.69738287
-0.73933355	2.972503698	0.696931901	30.30882386	35.73332198	-39.40229218
3.065877112	4.029029846	0.266448408	10.6031313	11.28358443	3.029200453
1.604137939	2.08889774	2.815950382	18.42666426	14.47624218	18.28453444
2.075337629	1.855537502	1.935023539	22.62709557	12.4001539	95.27150902
1.71769517	2.028724722	3.133349957	15.60577614	0.364914029	12.09564294
2.952071815	0.697303775	3.205159458	19.01681907	15.26486194	14.77135987
0.020112207	5.141723625	0.445980299	18.30739339	9.507820035	7.696218437
2.126938129	3.859733593	2.017021229	20.43378026	21.42589551	28.20883267
2.026066269	2.604324245	1.69370659	20.46833875	46.00681309	22.69738287
0.73933355	2.972503698	0.696931901	30.30882386	35.73332198	39.40229218
1.814174424	2.808642083	1.801063529	19.5330914	18.49595634	26.82855254

REO_qtm_x	REO_qtm_y	REO_qtm_z	REC_qtm_x	REC_qtm_y	REC_qtm_z
4.239273518	-0.561624733	-0.607934651	-15.28156111	-1.807616187	12.95554972
1.493124192	-1.090338193	0.475438007	-9.965856409	11.0646657	3.33167572
3.045100367	0.315201288	-0.269732237	1.487338218	10.81635517	10.2896917
3.518282142	-0.108983865	2.098653557	3.10800139	21.36167698	7.168053595
2.571203621	-1.913276308	-1.767062143	-4.243393698	27.59750903	8.587122753
3.881539284	-0.397359325	-0.05237274	-2.717393141	16.81518816	11.7279107
2.598426687	-1.641281277	2.211436041	-14.27681199	27.94161661	13.07392862
3.608996652	-1.469886642	0.078311145	-10.4964825	32.08557429	12.91743485
3.938972977	-0.767432996	-0.523210302	-16.03415076	32.75818553	8.90390792
4.239273518	0.561624733	0.607934651	15.28156111	1.807616187	12.95554972
1.493124192	1.090338193	0.475438007	9.965856409	11.0646657	3.33167572
3.045100367	0.315201288	0.269732237	1.487338218	10.81635517	10.2896917
3.518282142	0.108983865	2.098653557	3.10800139	21.36167698	7.168053595
2.571203621	1.913276308	1.767062143	4.243393698	27.59750903	8.587122753
3.881539284	0.397359325	0.05237274	2.717393141	16.81518816	11.7279107
2.598426687	1.641281277	2.211436041	14.27681199	27.94161661	13.07392862
3.608996652	1.469886642	0.078311145	10.4964825	32.08557429	12.91743485
3.938972977	0.767432996	0.523210302	16.03415076	32.75818553	8.90390792
3.210546605	0.91837607	0.89823898	8.623443247	20.24982085	9.883919508

LEO_qtm_x	LEO_qtm_y	LEO_qtm_z	LEC_qtm_x	LEC_qtm_y	LEC_qtm_z
0.759442232	-1.079545833	1.519794254	11.94884706	1.368933028	5.490589572
-0.595611243	0.548953482	-1.880142911	20.07726122	19.29405895	9.339986442
-1.151554614	1.723491458	-1.021513376	14.18843022	24.24185478	5.247085524
0.335693708	0.119355412	1.279241428	19.13463855	13.52906732	-4.535964236
0.684465313	1.995548331	-1.936291042	15.80486727	13.9524593	5.926378609
-0.275111179	-0.528493099	0.684793294	21.24922059	7.442195514	4.562653592
1.155781903	-0.202963777	-0.854247867	21.33632716	27.87218938	16.52983375
0.672162789	1.330359069	-1.000340323	25.02453723	17.86908208	40.94955471
0.572773654	-0.953798774	0.047588054	34.05578061	38.94471021	35.0043268
0.759442232	1.079545833	1.519794254	11.94884706	1.368933028	5.490589572
0.595611243	0.548953482	1.880142911	20.07726122	19.29405895	9.339986442
1.151554614	1.723491458	1.021513376	14.18843022	24.24185478	5.247085524
0.335693708	0.119355412	1.279241428	19.13463855	13.52906732	4.535964236
0.684465313	1.995548331	1.936291042	15.80486727	13.9524593	5.926378609
0.275111179	0.528493099	0.684793294	21.24922059	7.442195514	4.562653592
1.155781903	0.202963777	0.854247867	21.33632716	27.87218938	16.52983375
0.672162789	1.330359069	1.000340323	25.02453723	17.86908208	40.94955471
0.572773654	0.953798774	0.047588054	34.05578061	38.94471021	35.0043268
0.689177404	0.942501026	1.135994728	20.31332332	18.27939451	14.17626369

Participant	AE_REO_leap	AE_REC_leap	AE_LEO_leap	AE_LEC_leap	AE_REO_qtm	AE_REC_qtm
1	3.634	41.226	4.255	14.010	2.214	42.573
2	4.700	13.621	19.798	14.553	3.547	25.270
3	20.069	32.990	6.286	18.566	5.199	50.034
4	5.495	19.542	4.853	19.936	2.097	57.627
5	5.075	12.564	3.245	13.895	4.783	26.008
6	7.265	34.804	37.222	13.397	6.211	27.970
7	3.910	25.602	15.770	38.777	3.796	44.884
8	4.870	20.302	7.685	16.396	1.461	17.158
9	5.210	69.375	5.156	56.187	1.682	69.476
10	3.242	72.080	2.717	56.146	1.872	75.380
11	3.274	40.085	3.602	25.278	1.501	37.601
12	2.444	18.040	2.559	36.467	2.459	19.883
13	5.764	28.527	3.935	26.261	2.999	29.682
14	4.211	62.827	5.701	64.340	4.298	63.193
15	3.781	43.846	4.343	45.150	2.941	43.573
16	5.249	22.374	6.128	16.408	2.175	24.557
17	3.243	25.772	3.233	48.602	1.400	21.680
18	2.576	48.030	3.912	13.392	1.812	44.783
19	3.166	45.210	4.702	53.359	5.032	42.380
20	3.169	30.604	4.188	41.314	3.633	25.633
Medel	5.0172	35.3711	7.4646	31.6218	3.0555	39.4674
SD	3.7465	17.5413	8.2264	17.4829	1.4403	17.0335

AE_LEO_qtm	AE_LEC_qtm	.EAP _AE_REO_:	.EAP _AE_REO_:	.EAP _AE_REO_:	.EAP _AE_REC_:	.EAP _AE_REC_\
5.929	34.078	1.177	1.576	2.278	29.182	24.301
1.827	37.293	2.509	2.920	2.064	8.614	7.614
3.279	43.680	15.145	8.586	7.773	24.010	11.246
3.193	46.202	4.219	1.988	2.261	10.657	10.056
3.240	38.922	1.990	3.908	1.590	4.651	7.742
3.855	30.594	4.635	4.512	1.943	23.257	13.488
2.234	49.651	1.723	2.420	1.708	12.560	16.366
1.976	7.647	3.138	2.663	1.846	5.798	14.806
3.452	62.517	2.544	4.155	1.086	5.796	56.326
1.926	56.580	1.406	1.526	2.174	23.434	27.892
3.206	22.996	2.231	1.135	1.448	17.653	27.886
1.812	22.645	1.563	1.216	1.286	10.394	12.208
1.958	27.298	1.498	5.340	0.808	13.763	3.866
4.496	63.067	0.963	3.858	1.081	41.044	27.044
2.948	47.461	2.361	1.758	1.486	26.304	30.520
3.675	15.089	1.830	4.373	1.327	15.358	8.275
3.211	45.144	0.741	1.122	2.754	9.848	16.970
3.176	14.530	1.750	1.326	0.941	34.355	13.724
3.846	35.863	1.703	2.197	1.154	21.309	29.740
1.759	32.487	1.591	2.391	1.061	10.154	10.648
3.0499	36.6873	2.7359	2.9485	1.9035	17.4070	18.5359
1.0605	15.5513	3.0815	1.8484	1.4782	10.1262	12.2069

.EAP _AE_REC_Z	.EAP _AE_LEO_X	.EAP _AE_LEO_Y	LEAP _AE_LEO_Z	LEAP _AE_LEC_X	.EAP _AE_LEC_Y	AP _AE_LEC
12.232	1.231	2.689	2.322	7.023	8.676	5.809
3.994	10.942	2.639	15.463	5.291	9.392	6.932
16.097	2.923	4.251	2.707	9.533	13.384	5.777
5.647	1.758	1.311	3.871	10.667	10.612	7.878
5.476	1.830	2.139	0.862	6.777	9.461	5.131
18.355	17.786	18.855	21.215	9.518	7.204	4.126
12.473	6.977	12.297	3.783	25.015	17.702	17.413
11.020	5.010	4.344	2.855	11.097	4.906	7.706
38.285	2.378	3.407	1.869	49.027	16.712	17.217
60.692	1.063	1.526	1.394	36.145	10.499	39.114
21.053	0.984	1.693	2.840	11.507	17.686	11.185
5.509	1.012	1.790	1.028	8.971	10.415	31.238
24.052	2.676	2.505	0.624	21.248	8.950	9.718
38.180	3.520	3.561	2.025	36.179	50.816	10.733
12.317	3.800	1.651	0.543	33.977	20.897	16.085
10.475	2.460	3.886	3.464	7.750	12.122	5.066
12.757	0.357	1.021	2.974	3.466	48.308	2.995
22.651	2.072	2.368	1.421	3.455	9.519	6.573
22.668	2.122	3.496	1.298	22.973	36.986	22.323
24.411	1.814	2.809	1.801	19.533	18.496	26.829
18.9172	3.6359	3.9118	3.7179	16.9576	17.1372	12.9923
13.8588	4.1232	4.2404	5.1848	13.0733	13.0819	9.9966

QTM_AE_REO_	QTM_AE_REO_	QTM_AE_REO_	QTM_AE_REC_	QTM_AE_REC_	QTM_AE_REC_	QTM_AE_LEO_
0.933	1.358	1.136	31.165	12.327	23.913	4.538
1.534	2.403	1.548	8.220	12.106	19.076	0.742
3.080	2.135	2.820	40.662	26.565	9.844	1.210
0.938	0.979	1.131	20.576	16.570	50.618	1.349
1.558	3.080	2.752	5.916	5.059	24.330	2.190
2.188	3.123	4.732	23.206	8.022	12.322	1.237
1.681	1.143	3.071	3.830	31.940	30.536	1.167
0.775	0.996	0.320	4.275	10.175	12.288	1.295
0.656	0.657	1.102	14.726	40.322	49.501	1.040
0.554	0.912	1.396	23.933	64.188	28.486	0.732
1.182	0.455	0.453	17.948	21.039	24.464	1.293
0.811	1.161	1.742	11.119	6.211	13.712	0.721
0.960	1.574	2.259	8.581	26.987	6.946	0.519
2.233	1.739	2.646	44.505	37.416	23.212	2.565
1.485	1.883	1.277	29.742	17.899	25.168	1.063
1.248	0.970	1.240	18.376	9.737	8.765	1.431
0.564	0.673	0.927	9.810	14.263	10.431	1.466
1.324	0.685	0.877	41.377	14.633	7.074	1.135
2.055	1.856	3.646	19.343	19.320	30.544	2.168
3.211	0.918	0.898	8.623	20.250	9.884	0.689
1.4485	1.4350	1.7986	19.2965	20.7514	21.0557	1.4275
0.7724	0.7782	1.1498	12.6567	14.2377	12.7533	0.9028

QTM_AE_LEO_	QTM_AE_LEO_	QTM_AE_LEC_	QTM_AE_LEC_	QTM_AE_LEC_Z	DIFF REO X
1.635	3.242	3.552	3.863	4.173	0.24
1.367	0.749	21.118	28.501	7.700	0.97
0.966	2.506	24.341	16.955	31.453	12.06
2.425	0.983	20.582	23.675	32.274	3.28
1.672	1.435	9.432	7.091	36.133	0.43
2.640	2.157	13.671	18.496	19.577	2.45
1.445	0.834	32.635	27.999	19.846	0.04
0.893	0.939	4.849	4.059	2.924	2.36
2.140	1.981	55.255	19.837	17.259	1.89
0.900	1.347	34.996	39.922	10.604	0.85
1.224	2.485	7.257	14.817	14.709	1.05
0.521	1.288	11.661	16.142	7.004	0.75
0.987	1.327	22.192	11.400	8.447	0.54
2.182	2.485	36.334	13.206	47.972	-1.27
1.271	2.004	37.777	17.653	18.090	0.88
2.092	2.182	4.100	5.128	12.566	0.58
2.350	1.087	4.318	3.389	44.687	0.18
2.680	1.107	2.057	6.109	12.381	0.43
2.751	1.238	11.664	18.229	26.462	-0.35
0.943	1.136	20.313	18.279	14.176	-1.62
1.6541	1.6256	18.9053	15.7375	19.4218	
0.7006	0.6986	14.4832	9.5684	13.0579	

DIFF REO y	DIFF REO z	DIFF REC X	DIFFREC y	DIFF REC z	DIFF LEO x	DIFF LEO y	DIFF LEO z	DIFF LEC x
0.22	1.14	-1.98	11.97	-11.68	-3.31	1.05	-0.92	3.47
0.52	0.52	0.39	-4.49	-15.08	10.2	1.27	14.71	-15.83
6.45	4.95	-16.65	-15.32	6.25	1.71	3.29	0.2	-14.81
1.01	1.13	-9.92	-6.51	-44.97	0.41	-1.11	2.89	-9.91
0.83	-1.16	-1.26	2.68	-18.85	-0.36	0.47	-0.57	-2.66
1.39	-2.79	0.05	5.47	6.03	16.55	16.21	19.06	-4.15
1.28	-1.36	8.73	-15.57	-18.06	5.81	10.85	2.95	-7.62
1.67	1.53	1.52	4.63	-1.27	3.72	3.45	1.92	6.25
3.5	-0.02	-8.93	16	-11.22	1.34	1.27	-0.11	-6.23
0.61	0.78	-0.5	-36.3	32.21	0.33	0.63	0.05	1.15
0.68	1	-0.29	6.85	-3.41	-0.31	0.47	0.36	4.25
0.05	-0.46	-0.72	6	-8.2	0.29	1.27	-0.26	-2.69
3.77	-1.45	5.18	-23.12	17.11	2.16	1.52	-0.7	-0.94
2.12	-1.56	-3.46	-10.37	14.97	0.96	1.38	-0.46	-0.16
-0.12	0.21	-3.44	12.62	-12.85	2.74	0.38	-1.46	-3.8
3.4	0.09	-3.02	-1.46	1.71	1.03	1.79	1.28	3.65
0.45	1.83	0.04	2.71	2.33	-1.11	-1.33	1.89	-0.85
0.64	0.06	-7.02	-0.91	15.58	0.94	-0.31	0.31	1.4
0.34	-2.49	1.97	10.42	-7.88	-0.05	0.75	0.06	11.31
1.47	0.16	1.53	-9.6	14.53	1.12	1.87	0.67	-0.78

DIFF LEC y	DIFF LEC z
4.81	1.64
-19.11	-0.77
-3.57	-25.68
-13.06	-24.4
2.37	-31
-11.29	-15.45
-10.3	-2.43
0.85	4.78
-3.12	-0.04
-29.42	28.51
2.87	-3.52
-5.73	24.23
-2.45	1.27
37.61	-37.24
3.24	-2
6.99	-7.5
44.92	-41.69
3.41	-5.81
18.76	-4.14
0.22	12.65
