# Supplementary material for: Evaluation of a new clinical test protocol for pointing acuity - An initial validity study
Source: PLoS One. 2026 Aug 19;21(8):e0356388. doi: 10.1371/journal.pone.0356388 (PMC13489416; doi:10.1371/journal.pone.0356388)
Supplement: Supplementary file 2 [file pone.0356388.s001.docx]

| Table S1. Comparison of mean AE measurements between eyes closed and eyes open | | | | | |
| --- | --- | --- | --- | --- | --- |
|  | Mean | Min | Max | z-value | P -value |
|  |  |  |  |  |  |
| Right hand Eyes closed | 35.9 (16.8) | 18.7 | 73.7 | -3.9 | <.001 |
| Right hand Eyes open | 4.0 (2.3) | 2.2 | 12.6 |  |  |
| Left hand Eyes closed | 34.2 (15.1) | 12.0 | 63.7 | -3.9 | <.001 |
| Left hand Eyes open | 5.3 (4.2) | 2.2 | 20.5 |  |  |
| Results are presented in mean, standard deviation (SD), minimum and maximum absolute error measurements (AE). Wilcoxon Signed rank test for comparison of mean between eyes open and closed by Qualisys Motion Capture (QMC) and Leap Motion Controller (LMC) measurements. P-value with a significance level of .05. | | | | | |
